# Theretofore Highest Efficiency in Vacuum‐Deposited Organic Solar Cells Originating From Triarylamine‐Based Small‐Molecule Donors Containing Fused Heterocycle Units

**DOI:** 10.1002/advs.76486

**Published:** 2026-07-13

**Authors:** Yun‐Fei Li, Le‐Shan Dai, Zuo‐Chang Chen, Shi‐Long Xiong, Bin‐Wen Chen, Ke‐Yue Yang, Qiu Xiong, Yi‐Dong Yan, Kun Cao, Da‐Qin Yun, Feng He, Lin‐Long Deng, Su‐Yuan Xie, Lan‐Sun Zheng

**Affiliations:** ^1^ State Key Laboratory for Physical Chemistry of Solid Surfaces Collaborative Innovation Center of Chemistry for Energy Materials Department of Chemistry College of Chemistry and Chemical Engineering Xiamen University Xiamen China; ^2^ Pen‐Tung Sah Institute of Micro‐Nano Science and Technology Xiamen University Xiamen China; ^3^ Shenzhen Grubbs Institute and Department of Chemistry Southern University of Science and Technology Shenzhen China; ^4^ College of Energy Xiamen University Xiamen China

**Keywords:** fused heterocycle, organic solar cells, small‐molecule donor, triarylamine, vacuum‐deposition

## Abstract

Vacuum‐deposited organic solar cells (v‐OSCs) are alternative candidates for photovoltaics technologies. Their advantages include well‐defined molecular structures, high purity of materials, excellent batch‐to‐batch reproducibility, and solution‐free fabrication process that enables good compatibility with underlying perovskite layer for tandem devices. Triarylamine‐based small‐molecule donors are commonly used in v‐OSCs, however, their power conversion efficiencies (PCEs) are constrained by low short‐circuit current density (*J*_sc_). Moreover, to match with the photocurrent of perovskite subcell, enhancing *J*_sc_ of v‐OSCs is highly demanded. Herein, four small‐molecule donors (named PT, PF, BT, and BF) based on triarylamine electron‐donating moieties are designed. Compared with linear analogues PT and PF, BT and BF with fused‐ring building blocks including benzothiophene and benzofuran exhibit smaller bandgaps, lower highest occupied molecular orbital (HOMO) energy levels, and higher hole mobilities. Consequently, v‐OSCs based on BT and BF exhibit surprisingly high *J*_sc_ values (17.91 and 18.13 mA cm^−2^, respectively), representing the highest reported *J*_sc_ for v‐OSCs. Moreover, the *J*_sc_ enhancement leads to improved PCEs of devices based on BT and BF (10.53% and 10.11%, respectively). Remarkably, the PCE of 10.53% for the BT‐based device represents the best PCE reported for v‐OSCs. This work opens a promising avenue to develop high‐performance small‐molecule donors for v‐OSCs.

## Graphical abstract

Fused‐ring engineering unlocks record efficiency in vacuum‐deposited organic solar cells. By replacing linear π‐bridges with fused‐ring analogues in triarylamine‐based donors, we achieve enhanced molecular planarity, red‐shift optical absorption, minimize energy loss and optimized phase separation. This design overcomes the short‐circuit current bottleneck, delivering a record efficiency of 10.53% for vacuum‐deposited organic solar cells.

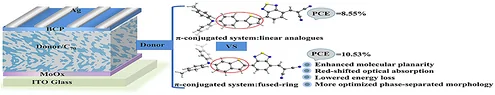

## 1 Introduction

Vacuum‐deposited organic solar cells (v‐OSCs) have emerged as alternative candidates for the future commercialization of photovoltaics technologies owing to their merits including well‐defined molecular structures, high purity of materials, excellent batch‐to‐batch reproducibility, pre‐eminent device stability, and solution‐free fabrication process that ensures excellent compatibility with the underlying perovskite layer for tandem devices [1, 2, 3, 4, 5, 6, 7]. However, the relatively low short‐circuit current density (*J*_sc_) of v‐OSCs has impeded the development of v‐OSCs as well as their integration into tandem devices with perovskite subcells [7, 8, 9, 10, 11]. Therefore, it is imperative to boost the *J*_sc_ of v‐OSCs.

As a critical component of v‐OSCs, small‐molecule donor materials play a pivotal role in determining the performance of v‐OSCs. The state‐of‐the‐art small‐molecule donors are based on the donor‐acceptor‐acceptor (D‐A‐A) molecular architecture, which exhibit strong absorption and well‐ordered molecular packing [7, 12]. Among various donor moieties, triarylamine stands out because of its merits including good electron‐donating ability, excellent hole transport capability, and convenience in chemical modification [13, 14, 15]. Thus, much effort has been dedicated to developing D‐A‐A type small‐molecule donors that incorporate triarylamines as the electron‐donating units. For example, Lin et al. synthesized a D‐A‐A configured small‐molecule donor DTDCTB, in which the ditolylaminothienyl D group is end‐capped with a dicyanovinylene terminal A unit through a benzothiadiazole central A moiety [12]. Owing to the strong electron‐donating nature of the ditolylaminothienyl unit, v‐OSCs based on DTDCTB: C_70_ exhibited a power conversion efficiency (PCE) of 5.81% with a *J*_sc_ of 14.68 mA cm^−2^. Modification of the triarylamine moiety can fine‐tune the molecular energy levels and optical properties of small‐molecule donors, which greatly affect their photovoltaic performance. For instance, Wong and co‐workers reported three donors DPDCTB, DPDCPB, and DTDCPB with different triarylamine D units including diphenylaminothiophene, triphenylamine, and ditolylaminophenylene, respectively [16]. Devices based on DTDCPB: C_70_ achieved a PCE of 6.8%, higher than that of DPDCTB: C_70_ and DPDCPB: C_70_. Forrest et al. further enhanced the PCE of DTDCPB: C_70_ to 9.6% with a *J*_sc_ of 15.8 mA cm^−2^ by device optimization [11]. Chou et al. incorporated indeno[1,2‐b]thiophene into the triarylamine D moieties, forming two donors MIDTP, and TIDTP, respectively [17]. The MIDTP based devices exhibited higher PCE of 4.2% than that of TIDTP based devices, which was ascribed to better hole mobility and higher crystallinity of MIDTP. In addition to tailoring the triarylamine D moiety, manipulation of the central A group such as pyrimidine [18], benzochalcogenodiazole [19], benzotriazole [20], thieno[3,4‐b]pyrazine [21], as well as the terminal A unit including nitro [22], 1,3‐indandione, and rhodamine [23] can effectively regulate the photovoltaic performance of these triarylamine based small‐molecule donors. Regrettably, all the reported triarylamine based D‐A‐A configured small‐molecule donors suffer from relatively small *J*_sc_, (<16 mA cm^−2^) which leaves much room for improvement.

Extending the π‐conjugated system of triarylamine is an effective approach to enhance its light‐harvesting ability and photocurrent. Therefore, in this study, fused‐ring segments such as benzothiophene and benzofuran were incorporated into the triarylamine moiety to construct D‐A‐A type small‐molecule donors and offered systematic comparisons with their linear counterparts. Four novel small‐molecule donors were designed and synthesized, including 2‐((7‐(5‐(4‐(di‐p‐tolylamino)phenyl)thiophen‐2‐yl)benzo[c][1,2,5]thiadiazol‐4‐yl)methylene) malononitrile (PT), 2‐((7‐(5‐(4‐(di‐p‐tolylamino)phenyl)furan‐2‐yl)benzo[c][1,2,5] thiadiazol‐4‐yl)methylene)malononitrile (PF), 2‐((7‐(6‐(di‐p‐tolylamino)benzo[b] thiophen‐2‐yl)benzo[c] [1,2,5]thiadiazol‐4‐yl)methylene)malononitrile (BT), and 2‐((7‐(6‐(di‐p‐tolylamino)benzo furan‐2‐yl) benzo[c][1,2,5]thiadiazol‐4‐yl)methylene)malononitrile (BF). Compared with their linear analogues, small‐molecule donors with fused‐ring building blocks demonstrated smaller bandgaps, lower highest occupied molecular orbital (HOMO) energy levels, and higher hole mobilities. Consequently, v‐OSCs based on BT and BF exhibited surprisingly high *J*_sc_ values (17.91 and 18.13 mA cm^−2^, respectively), which are the highest reported *J*_sc_ values for v‐OSCs. Moreover, the *J*_sc_ enhancement improved the PCEs of devices based on BT and BF (10.53% and 10.11%, respectively), which outclassed those of devices based on PT and PF (8.55% and 8.12%, respectively). Remarkably, the PCE of 10.53% represents the best PCE reported for v‐OSCs. This work demonstrates that incorporation of fused heterocycle units including benzothiophene and benzofuran into triarylamine moieties is a promising tactic to construct high‐performance small‐molecule donors for v‐OSCs.

## 2 Results and Discussion

The molecular structures of PT, PF, BT, and BF are illustrated in Figure 1a, while their synthetic routes are depicted in Scheme S1. Detailed synthesis, purification, and characterization procedures are described in Figures S1–S26. To gain insights into their natures, density functional theory (DFT) calculations were performed. Their HOMO and lowest unoccupied molecular orbital (LUMO) distributions and electrostatic potential are presented in Figure S27.Their HOMOs are largely delocalized on the electron‐donating groups and partially extended to the electron‐withdrawing units, whereas their LUMOs are mainly distributed on the electron‐withdrawing units and partially extended to the electron‐donating group. The HOMO and LUMO overlaps are anticipated to be favorable for efficient light harvesting. We also calculated the dipole moments of these molecules. As displayed in Figure S28, the molecular dipole moments of PT, PF, BT, and BF are 13.93, 12.83, 12.77, and 12.05 Debye, respectively. Such large dipole moments could facilitate the self‐assembly of molecules into antiparallel dimers, achieving more ordered molecular stacking and contributing to higher charge mobility.

**FIGURE 1:**
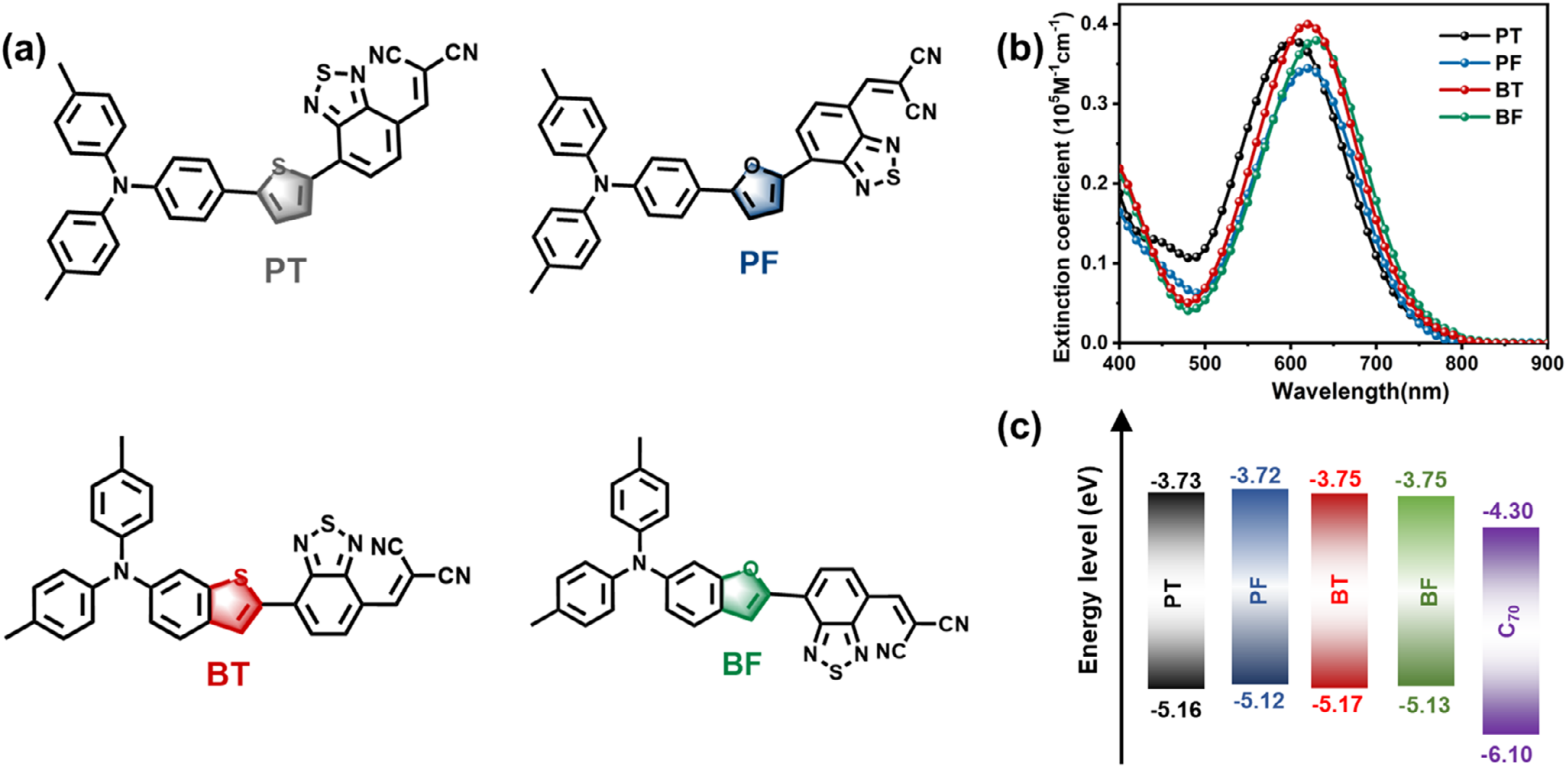
(a) Molecular structures of PT, PF, BT, and BF. (b) UV–vis absorption spectra of PT, PF, BT, and BF in dichloromethane solution. (c) Energy level alignment of PT, PF, BT, BF, and C_70_.

The optical properties of PT, PF, BT, and BF were investigated by ultraviolet‐visible (UV–vis) absorption spectra. As depicted in Figure 1b, all the four molecules display strong absorptions in the 500–800 nm range. The molar extinction coefficients (ε) of PT, PF, BT, and BF are 3.80 × 10^4^, 3.45 × 10^4^, 4.00 × 10^4^, and 3.80 × 10^4^ L mol^−1^ cm^−1^, with maximum absorption peaks at 604, 620, 621, and 628 nm, respectively. Compared with PT and PF, BT and BF with fused‐ring building blocks demonstrated higher molar extinction coefficients, which is in line with their higher oscillator strengths of the S_0_ to S_1_ transition from TD‐DFT calculations (Tables S1–S4). From solution to film (Figure S29), the maximum absorption peaks of PT, PF, BT, and BF are red‐shifted to 645, 641, 655, and 665 nm, respectively, implying the enhanced intermolecular π‐π interactions in the solid state [12]. The optical bandgaps calculated from the onset absorption of thin films for PT, PF, BT, and BF are 1.48, 1.50, 1.46, and 1.46 eV, respectively. Compared with the linear PT and PF, BT and BF with fused‐ring building moieties exhibited smaller bandgaps, which is expected to attain higher *J*_sc_ for the corresponding devices. The HOMO and LUMO energy levels of PT, PF, BT, and BF were measured by cyclic voltammetry (CV). The oxidation and reduction curves are depicted in Figure S31. The energy levels of these small‐molecule donors and C_70_ are shown in Figure 1c. The HOMO/LUMO energy levels of PT, PF, BT, and BF were calculated as −5.16/−3.73, −5.12/−3.72, −5.17/−3.75, and −5.13/−3.75 eV, respectively. Compared with the linear PT and PF, BT and BF exhibited slightly lower HOMO energy levels, which is beneficial for obtaining higher *V*_oc_. Thermogravimetric analysis (TGA) was conducted to assess the thermal properties of PT, PF, BT, and BF (Figure S32). PT, PF, BT, and BF exhibited excellent thermal stability with 5% weight loss at temperatures of 358°C, 342°C, 348°C, and 349°C, respectively, which is suitable for vacuum deposition techniques.

To reveal the molecular conformation and crystal packing of PT, PF, BT, and BF, single‐crystal X‐ray diffraction analyses were conducted, and the detailed data were listed in Table S5. As illustrated in Figure 2a–d, different conformations were observed for these molecules. For the PT and BT molecules, the sulfur atoms in the thiophene or benzothiophene moiety and the 2,1,3‐benzothiadiazole (BT) unit are located on the same side along the molecular backbone. On the contrary, for the PF and BF molecules, the oxygen atom of the furan or benzofuran moiety and the sulfur atom in the BT unit are situated on the opposite side along the molecular backbone, due to the presence of intramolecular hydrogen bonding. Moreover, all the four molecules display coplanar conformations, as suggested by the small dihedral angles between the thiophene/furan/benzothiophene/benzofuran moiety and the BT unit. Due to the asymmetric D‐A‐A configuration, all these four molecules adopt an antiparallel arrangement to form centrosymmetric dimers (Figure 2e–h). For the PT dimers, the thiophene and BT units of adjacent PT molecules form offset stacking, with a π‐π interaction distance of 3.26–3.41 Å. In contrast, the benzene moiety of BT unit in adjacent BT molecules forms strong face‐to‐face stacking, with a π‐π interaction distance of 3.41 Å. For the PF dimers, the adjacent BT and furan units are misaligned, preventing effective π‐π interactions, despite the relatively short face‐to‐face distance of 3.31 Å. The intermolecular interactions in the BF dimers are analogous to those of the PT dimers, with adjacent furan and BT units forming offset stacking and a π‐π interaction distance of 3.37 Å. Compared with PT, PF, and BF, the crystal packing of BT is more compact along the *a*‐, *b*‐, and *c*‐axes (Figure S33). Particularly, the dimer spacing along the *a*‐axis in the crystals of PT, PF, and BF is larger, whereas the dimer spacing in BT is notably smaller. This closer packing arrangement could facilitate intermolecular charge transfer and enhance charge carrier mobility.

**FIGURE 2:**
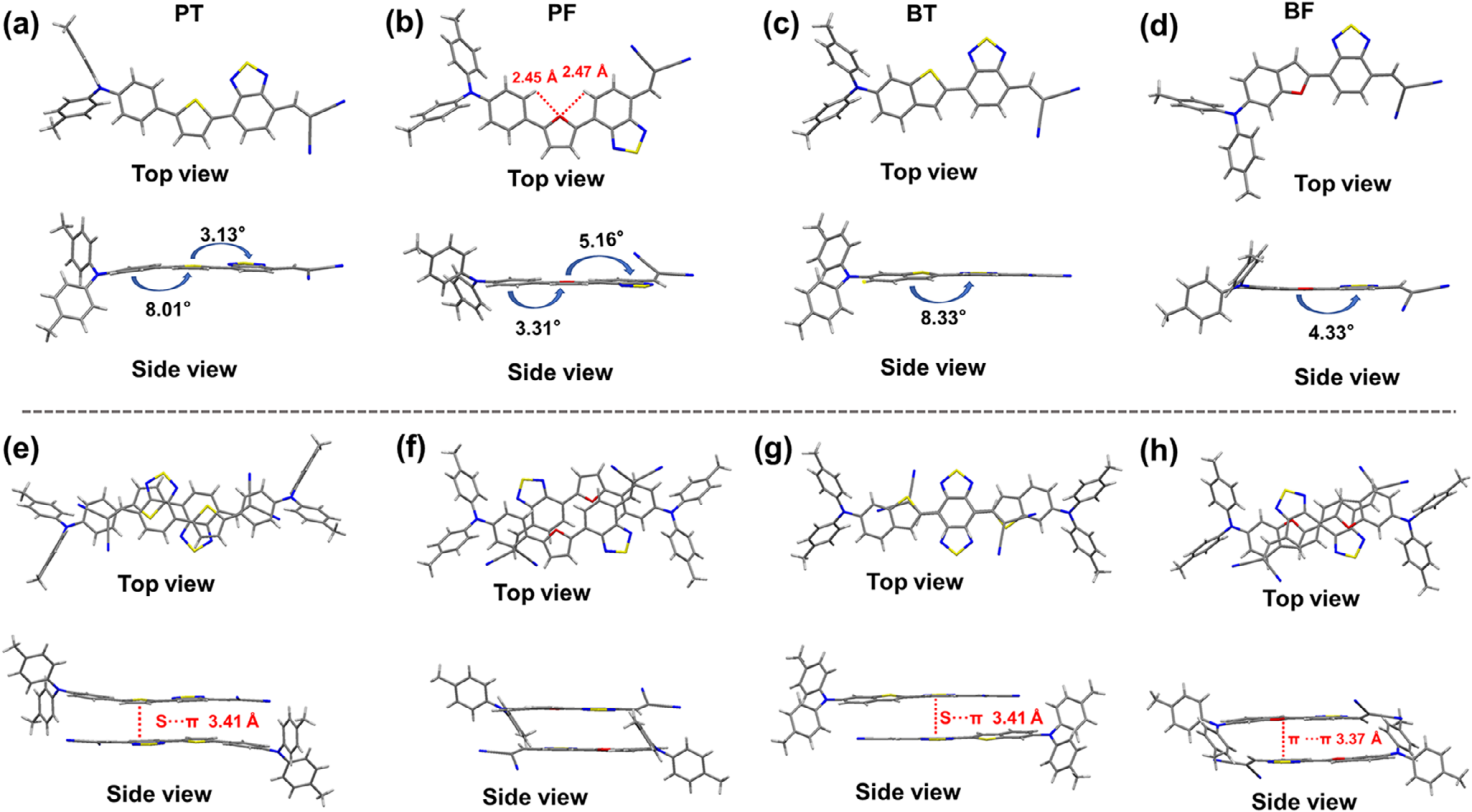
The monomer crystal structure and the dihedral angles for PT (a), PF (b), BT (c), and BF (d). The dimers and π‐π stacking distances for PT (e), PF (f), BT (g), and BF (h).

The v‐OSCs with the device structure of ITO/MoO_x_/donor: C_70_/BCP/Ag (Figure 3a) were fabricated to evaluate the photovoltaic performance of PT, PF, BT, and BF. To get the optimum photovoltaic performance, the donor:acceptor (D:A) ratio and active layer thickness were optimized and summarized in Tables S6–S9. The best PCE data were obtained with the D:A ratio of 1:2 and the active layer thickness at ∼80 nm. The optimal thickness of the active layer was verified by stylus profilometer and cross‐sectional SEM images (Figures S34 and S35). The current density–voltage (*J*–*V*) curves of the optimal devices are depicted in Figure 3b, and the relevant photovoltaic performance data are summarized in Table 1. The statistical PCE distributions for all devices are further shown in Figure 3c, reveling their excellent reproducibility. As displayed in Table 1, devices based on PT: C_70_ and PF: C_70_ exhibited relatively lower PCEs of 8.55% and 8.12%, respectively. In contrast, both BT: C_70_ and BF: C_70_ devices achieved enhanced PCEs of 10.53% and 10.11%, respectively. A certified PCE of 10.33% was obtained for the champion BT: C_70_ devices from the Test and Calibration Center of New Energy Device and Module, Shanghai Institute of Microsystem and Information Technology, Chinese Academy of Sciences (SIMIT) (Figure 3d and Figure S36). Remarkably, the PCE of 10.53% for the BT: C_70_ devices is the record efficiency for v‐OSCs (Figure 3e and Table S10). Moreover, both BT and BF devices exhibited surprisingly high *J*_sc_ values (17.91 and 18.13 mA cm^−2^, respectively), which are the highest *J*_sc_ values for v‐OSCs to date. The superior performance of the BT and BF devices is related to the observed improvements in *V*_oc_, *J*_sc_, and FF. Considering that the HOMO energy level differences are relatively small, the observed *V*_oc_ enhancement of BT and BF devices is mainly originated from the reduced energy loss, especially the reduced non‐radiative energy loss (details are discussed in the energy loss analysis). The enhanced *J*_sc_ in BT and BF devices is also verified by the external quantum efficiency (EQE) spectra (Figure 3f). At wavelengths of 550–900 nm, both BT and BF devices exhibited higher photocurrent response compared to that of PT and PF devices. To clearly distinguish the relative contributions of donor absorption versus C_70_ absorption in the blend, the absorption spectra of four donors and C_70_ in thin films were measured in Figures S29 and S30. A comparison with the absorption spectra of the donor and C_70_ reveals that the absorption of the blend at shorter wavelength region (300–550 nm) is dominated by C_70_ and the absorption at longer wavelength region (550–900 nm) mainly originates from the donor. Thus, the higher photocurrent response in the longer wavelength region for BT and BF devices is most likely originated from the donor.

**FIGURE 3:**
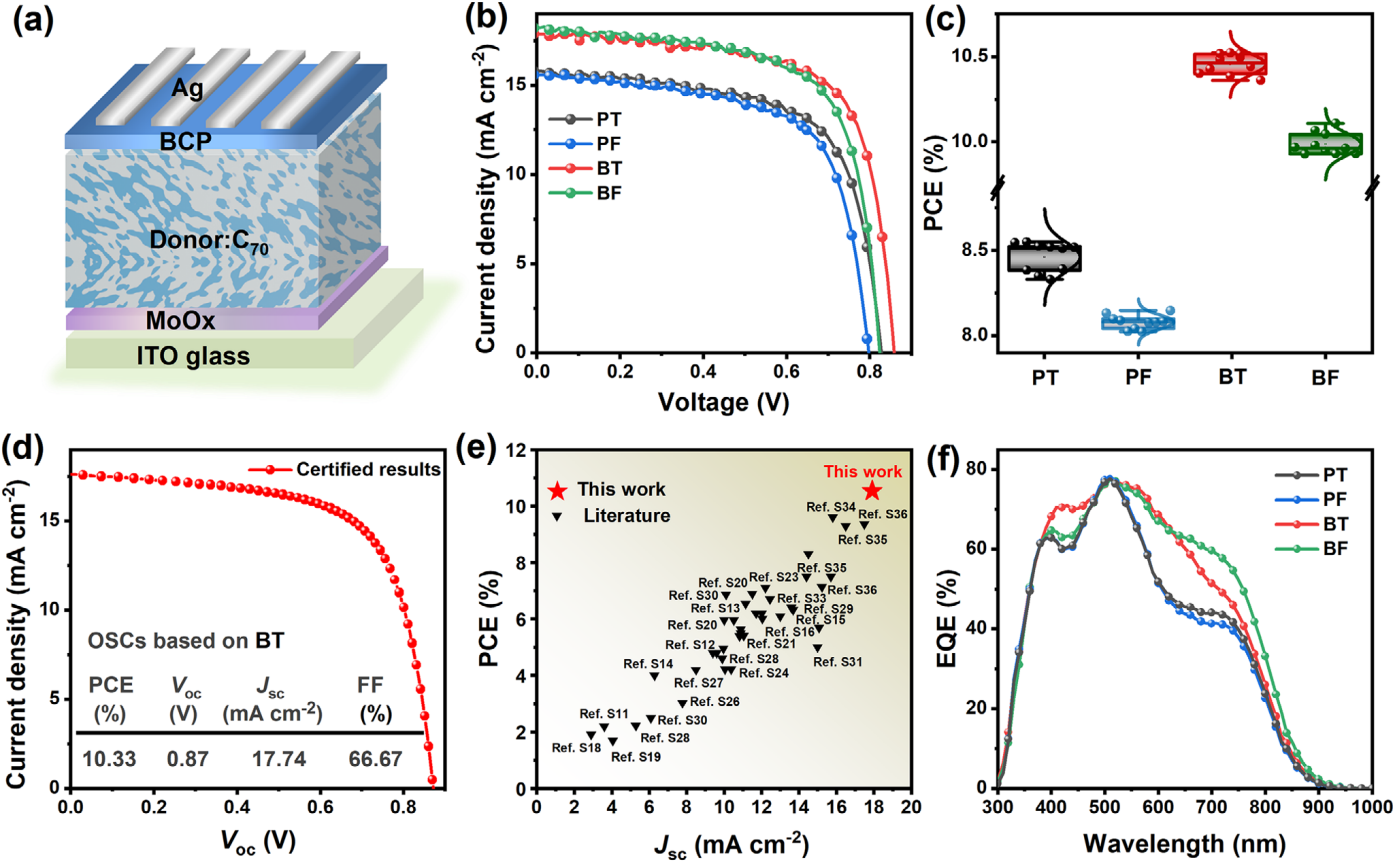
(a) Device structure of v‐OSCs. (b) *J‐* *V* curves of PT, PF, BT, and BF based devices. (c) Statistics of PCE for PT, PF, BT, and BF based devices. (d) Certified results of BT based devices. (e) Summary of PCE and *J*_sc_ parameters for previously reported v‐OSCs. (f) EQE spectra of PT, PF, BT, and BF based devices.

**TABLE 1:** Device characteristics of v‐OSCs under simulated AM 1.5G illumination.

Device	*V*_oc_ (V)	*J*_sc_ (mA cm^−2^)	FF (%)	PCE (%)
PT: C_70_	0.83 ± 0.01 (0.83)	15.52 ± 0.14 (15.73)	65.80 ± 0.25 (65.62)	8.46 ± 0.08 (8.55)
PF: C_70_	0.80 ± 0.01 (0.80)	15.47 ± 0.26 (15.59)	65.23 ± 0.55 (65.33)	8.08 ± 0.04 (8.12)
BT: C_70_	0.86 ± 0.01 (0.86)	17.83 ± 0.09 (17.91)	68.28 ± 0.30 (68.48)	10.45 ± 0.06 (10.53)
BF: C_70_	0.82 ± 0.01 (0.82)	17.97 ± 0.09 (18.13)	67.38 ± 0.23 (67.68)	9.99 ± 0.06 (10.11)

The photovoltaic parameters were statistically derived from 10 devices fabricated across two independent batches.

To elucidate the reasons responsible for the superior *J*_sc_ and FF of devices based on BT and BF, the charge generation, transport and recombination behaviors of these devices were investigated. First, the hole mobilities (*µ*_h_) and electron mobilities (*µ*_e_) of the blend films were achieved by the space‐charge‐limited current (SCLC) method. As shown in Figure 4a and Figure S37, the devices based on PT: C_70_ and PF: C_70_ blend films presented *µ*_h_ of 5.92 and 4.40 × 10^−5^ cm^2^ V^−1^ s^−1^, and *µ*_e_ of 9.28 and 8.13 × 10^−5^ cm^2^ V^−1^ s^−1^, leading to *µ*_h_/*µ*_e_ ratios of 0.64 and 0.54, respectively. Notably, devices based on BT: C_70_ and BF: C_70_ showcased *µ*_h_ of 8.38 and 6.56 × 10^−5^ cm^2^ V^−1^ s^−1^, alongside *µ*_e_ of 11.2 and 9.83 × 10^−5^ cm^2^ V^−1^ s^−1^, corresponding to *µ*_h_/*µ*_e_ ratios of 0.75 and 0.67, respectively. The higher and more balanced charge transport of the BF: C_70_ and BT: C_70_ devices contribute to their higher *J*_sc_ and FF. Subsequently, the correlation between the photocurrent density (*J*_ph_) and the effective voltage (*V*_eff_) was analyzed to investigate the exciton dissociation and charge extraction within the active layers of devices. As shown in Figure 4b, the exciton dissociation efficiency (*η*_diss_) for devices based on PT: C_70_, PF: C_70_, BT: C_70_, and BF: C_70_ is 92.18%, 90.10%, 93.64%, and 93.02%, respectively. The slightly higher *η*_diss_ values suggest the more efficient exciton dissociation and charge extraction process for the BT: C_70_ and BF: C_70_ based devices. To further investigate the charge recombination mechanism, the relationship between *J*_sc_ and light intensity (*P*_light_) was probed (Figure 4c). The relationship between *J*_sc_ and *P*_light_ can be analyzed as *J*_sc_ ∝ *P*_light_ *^S^*, where *S* is defined as an exponential factor reflecting the degree of bimolecular recombination. The *S* values for the BT: C_70_ (96.73%) and BF: C_70_ (95.16%) devices are slightly higher than those of PT: C_70_ (94.41%) and PF: C_70_ (92.48%) devices, revealing suppressed bimolecular recombination in these devices. These results reveal that the higher and more balanced charge transport as well as more efficient exciton dissociation and charge extraction with suppressed bimolecular recombination, predominantly contributing to the *J*_sc_ improvement for the BF: C_70_ and BT: C_70_ devices. To explain the improvement in *V*_oc_, we measured the energy loss (*E*_loss_) of devices based on PT, PF, BT, and BF. The *E*_loss_ can be calculated according to the equation *E*_loss_ = *E*_g_‐q*V*_oc_, where q, *E*_g_, and *V*_oc_ are the elementary charge, optical bandgap of the active layer and photovoltaic *V*_oc_. The calculated *E*_loss_ values for the BT (0.749 eV) and BF (0.727 eV) devices are lower than those of the PT (0.785 eV) and PF (0.756 eV) devices, resulting in the increased *V*_oc_ for the BT and BF devices. Considering the relatively large non‐radiative recombination energy loss (Δ*E*_nr_) of OSCs that has the most significant impact on the *E*_loss_, we further investigated the Δ*E*_nr_ of devices based on PT, PF, BT, and BF by using the electroluminescence external quantum efficiency (EQE_EL_) measurements [24, 25, 26, 27]. As displayed in Figure 4d, the Δ*E*_nr_ values for the PT, PF, BT, and BF devices are 0.350, 0.343, 0.331, and 0.332 eV, respectively. The lower Δ*E*_nr_ values for the BT and BF devices indicate that BT and BF with fused‐ring building moieties have lower non‐radiative recombination energy loss, compared to their linear analogues PT and PF. The reduced energy loss, especially the reduced non‐radiative energy loss, could be the main reason for the increase of *V*_oc_ in the BT and BF devices.

**FIGURE 4:**
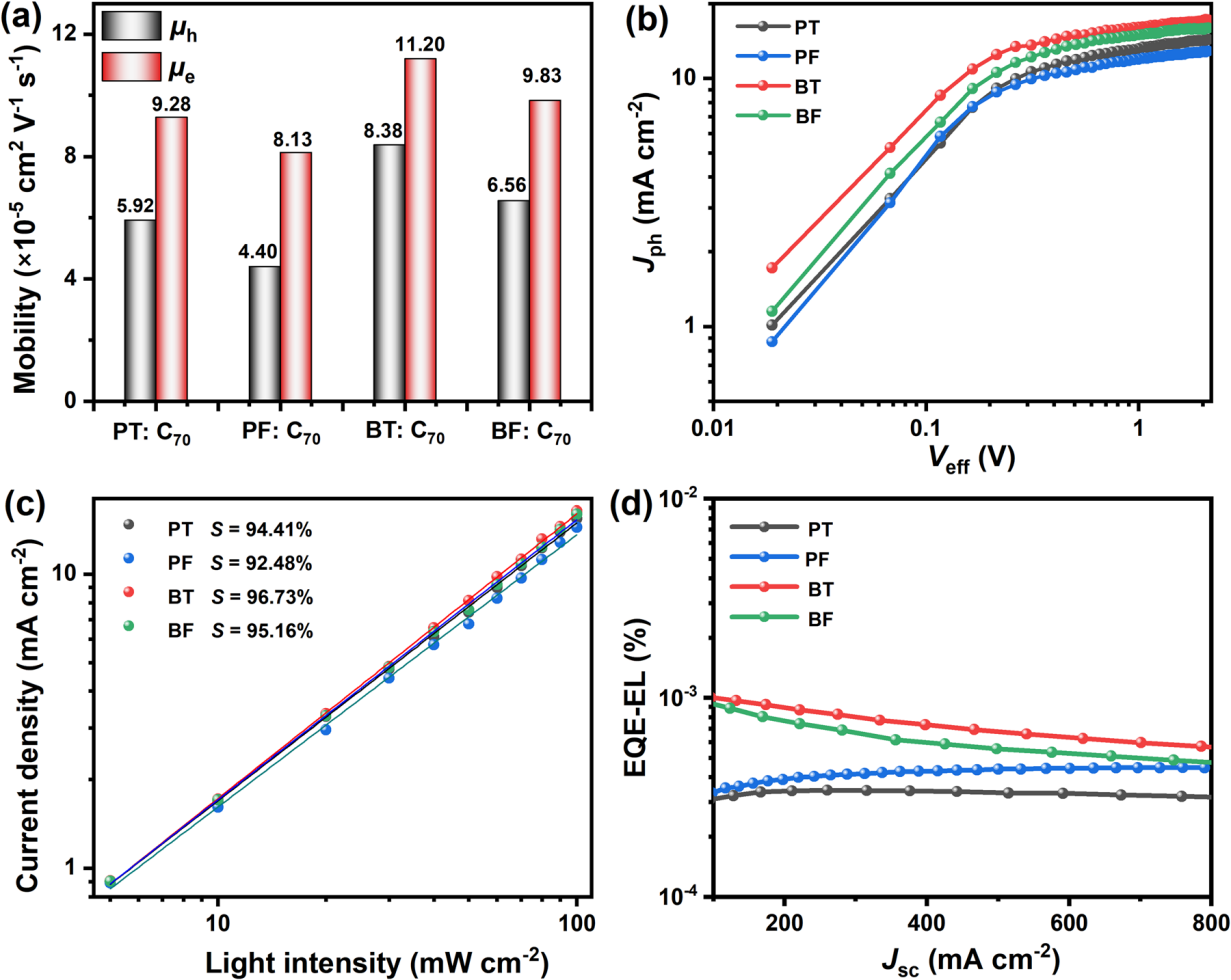
(a) Hole and electron mobilities, (b) *J*_ph_ versus *V*_eff_ curves, (c) *J*_sc_ versus light intensity curves, and (d) EQE_EL_ curves for devices based on PT, PF, BT, and BF.

Atomic force microscopy (AFM), transmission electron microscopy (TEM) and grazing‐incidence wide‐angle X‐ray scattering (GIWAXS) were applied to investigate the morphology of the blend films. As depicted in Figure S38, the PT: C_70_ and PF: C_70_ blend films displayed a slightly homogeneous surface morphology, with surface root mean square roughness (*R*_q_) values of 3.00 and 3.05 nm, respectively. In contrast, the BT: C_70_ and BF: C_70_ blend films exhibited relatively rougher surfaces with *R*_q_ values of 3.41 and 3.20 nm, respectively. Furthermore, the TEM images (Figure S39) revealed significant differences in the surface structures of the four blend films. Compared to the PT: C_70_ and PF: C_70_ blend films with uniform surface morphology, the BT: C_70_ and BF: C_70_ blend films exhibited a more pronounced fiber network structure, which facilitates exciton diffusion and charge transport [28]. The 2D GIWAXS patterns and line‐cut profiles of the PT, PF, BT and BF neat films in the in‐plane (IP) and out‐of‐plane (OOP) directions are shown in in Figure S40. As illustrated in Figure S40, all the four samples exhibited broad ring‐like scattering peaks, suggesting weak crystallinity with random orientations. Both PT and BT neat films exhibited a weak π‐π stacking peak at 1.80 Å^−1^ in both IP and OOP directions, corresponding to a π‐π stacking distance of 0.35 nm. Similarly, the BF neat films displayed a weak π‐π stacking peak at 1.82 Å^−1^ in both IP and OOP directions, corresponding to a π‐π stacking distance of 0.34 nm, which is consistent with the data of the single‐crystal X‐ray diffraction analysis. In contrast, the PF neat films exhibited a prominent lamellar stacking peak at 0.71 Å^−1^ with a lamellar stacking distance of 0.88 nm in both IP and OOP directions, and an apparent π‐π stacking peak at 1.86 Å^−1^ with a π‐π stacking distance of 0.34 nm in the IP direction. However, after being blended with C_70_, the diffraction patterns of all the blend films (as shown in Figure 5) are governed by C_70_ because of the much higher crystallinity of the C_70_ acceptor compared to small‐molecule donors [9]. The crystal coherence lengths (CCLs) of the BT: C_70_ and BF: C_70_ blend films, as calculated from the OOP (010) and IP (100) diffraction peaks, were determined to be 61.79 and 38.36 Å, and 55.27 and 35.47 Å, respectively. In contrast, the CCLs of the PT: C_70_ and PF: C_70_ blend films, were determined to be 52.46 and 33.63 Å, and 32.04 and 31.16 Å, respectively. Compared with PT: C_70_ and PF: C_70_ blend films, the BT: C_70_ and BF: C_70_ blend films exhibited higher CCL values, demonstrating their higher crystallinity. These results suggest that BT and BF with fused‐ring building blocks facilitate enhanced crystallization, as well as the formation of a more optimal phase‐separated morphology, thereby facilitating charge transport within the blend films [29, 30].

**FIGURE 5:**
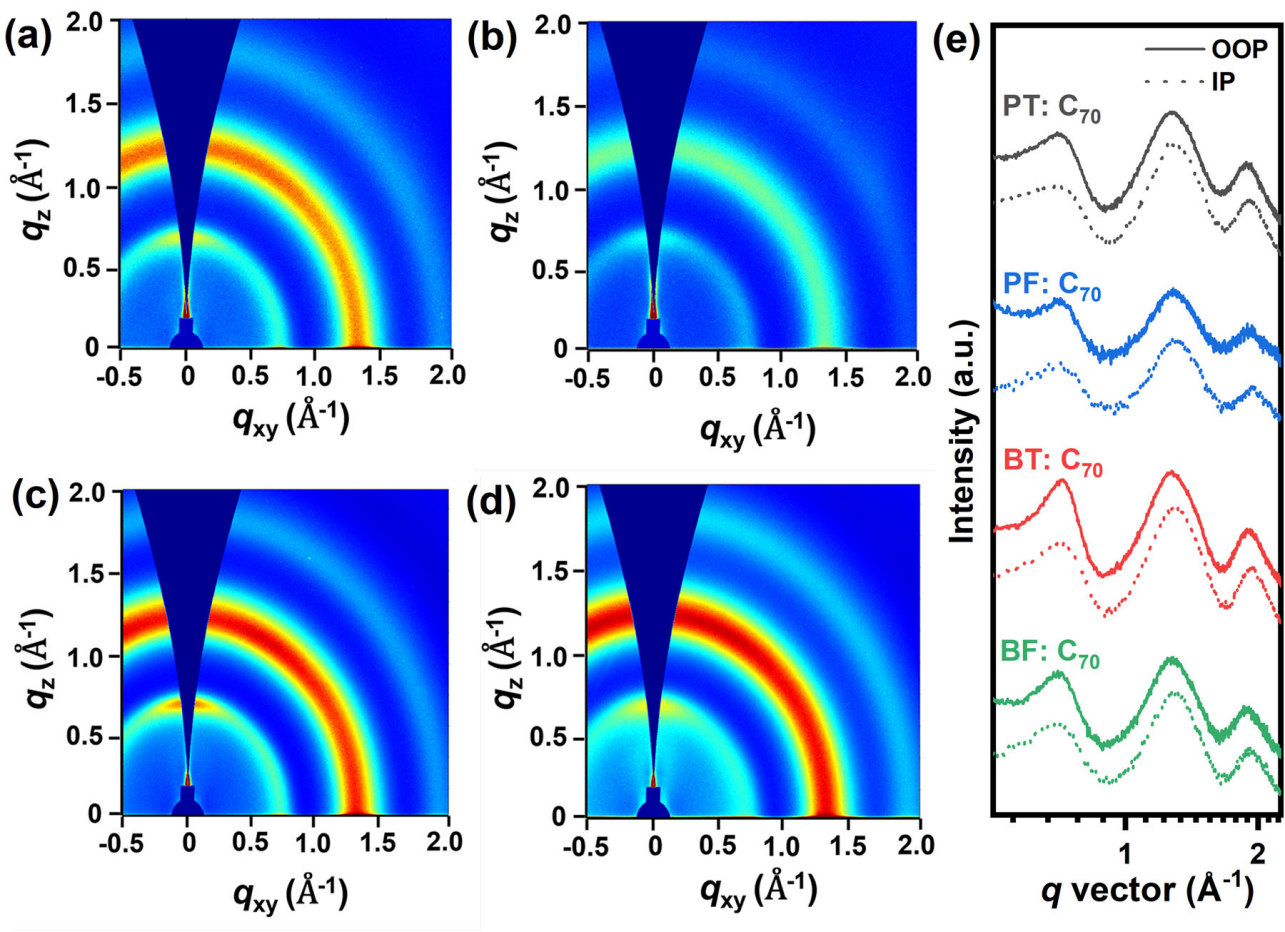
2D GIWAXS patterns of PT: C_70_ (a), PF: C_70_ (c), BT: C_70_ (b) and BF: C_70_ (d) blend films. (e) The corresponding line‐cut profiles along in‐plane and out‐of‐plane directions.

Considering the importance of device stability for v‐OSCs, the thermal stability and storage stability for BT: C_70_ and PT: C_70_ devices were measured. As shown in Figure S41, the BT: C_70_ devices maintained ∼96% of their initial PCEs after 360 days of storage in a nitrogen‐filled glove box, while the PT: C_70_ devices retained ∼92% of their initial PCEs. To further evaluate the thermal stability of v‐OSCs, both BT: C_70_ and PT: C_70_ devices were stressed at 85°C, as shown in Figure S42. The BT: C_70_ devices retained about 92% of their initial PCEs after 1008 h of thermal aging, surpassing that of the PT: C_70_ devices (∼88%). Our study demonstrates the synergistic effect of fused‐ring structure on both device efficiency and stability, and provides more practical application references for this material system.

## 3 Conclusions

In conclusion, four novel D‐A‐A configured small‐molecule donors based on triarylamine donor moieties were developed for v‐OSCs. Compared with the linear analogues PT and PF, BT and BF with fused‐ring building blocks such as benzothiophene and benzofuran exhibited smaller bandgaps, lower HOMO energy levels, and higher hole mobilities. As a result, v‐OSCs based on BT: C_70_ and BF: C_70_ demonstrated superior performance, achieving PCEs of 10.53% and 10.11%, respectively. Notably, both BT: C_70_ and BF: C_70_ devices exhibited surprisingly high *J*_sc_ values (17.91 and 18.13 mA cm^−2^, respectively), which are the highest reported *J*_sc_ values for v‐OSCs. Overall, this work indicates that incorporation of fused‐ring building blocks into triarylamine moieties of small‐molecule donors is an effective way to improve the photovoltaic performance and device stability of v‐OSCs.

## Author Contributions

**Kun Cao**: data curation, formal analysis, validation. **Su‐Yuan Xie**: project administration, funding acquisition, supervision. **Qiu Xiong**: data curation, formal analysis, methodology. **Zuo‐Chang Chen**: software, data curation, validation. **Ke‐Yue Yang**: data curation, validation, formal analysis. **Yi‐Dong Yan**: data curation, formal analysis, methodology. **Lin‐Long Deng**: writing – review and editing, conceptualization, supervision, funding acquisition, project administration. **Le‐Shan Dai**: methodology, data curation, formal analysis, writing – original draft. **Yun‐Fei Li**: writing – original draft, formal analysis, methodology, data curation. **Bin‐Wen Chen**: writing – review and editing, methodology, validation, supervision. **Da‐Qin Yun**: formal analysis, methodology, investigation. **Lan‐Sun Zheng**: project administration, supervision, funding acquisition. **Feng He**: writing – review and editing, investigation, supervision. **Shi‐Long Xiong**: data curation, methodology, formal analysis.

## Conflicts of Interest

The authors declare no conflicts of interest.

## Supporting information




**Supporting File**: advs76486‐sup‐0001‐SuppMat.docx.

## Data Availability

The data that support the findings of this study are available from the corresponding author upon reasonable request.

## References

[1] Q. Burlingame , X. Huang , X. Liu , C. Jeong , C. Coburn , and S. R. Forrest , “Intrinsically Stable Organic Solar Cells Under High‐Intensity Illumination,” Nature 573 (2019): 394–397, 10.1038/s41586-019-1544-1. 31501570

[2] Y. Lin , Y. Li , and X. Zhan , “Small Molecule Semiconductors for High‐Efficiency Organic Photovoltaics,” Chemical Society Reviews 41 (2012): 4245–4272, 10.1039/c2cs15313k. 22453295

[3] C. Duan and L. Ding , “The New Era for Organic Solar Cells: Small Molecular Donors,” Science Bulletin 65 (2020): 1597–1599, 10.1016/j.scib.2020.05.019. 36659033

[4] M. Kim , K. Lee , E.‐S. Kwon , et al., “Can Fully Vacuum‐Processed Perovskite Solar Cells Follow the Footprint of OLEDs?,” Advanced Energy Materials 16 (2026): 06169, 10.1002/aenm.202506169.

[5] I. Jeon , R. Sakai , T. Nakagawa , H. Setoguchi , and Y. Matsuo , “Stability of Diketopyrrolopyrrole Small‐Molecule Inverted Organic Solar Cells,” Organic Electronics 35 (2016): 193–198, 10.1016/j.orgel.2016.05.022.

[6] I. Jeon , R. Sakai , S. Seo , et al., “Engineering High‐Performance and Air‐Stable PBTZT‐stat‐BDTT‐8: PC_61_BM/PC_71_BM Organic Solar Cells,” Journal of Materials Chemistry A Materials for Energy & Sustainability 6 (2018): 5746–5751.

[7] A. Venkateswararao and K.‐T. Wong , “Small Molecules for Vacuum‐Processed Organic Photovoltaics: Past, Current Status, and Prospect,” Bulletin of the Chemical Society of Japan 94 (2021): 812–838, 10.1246/bcsj.20200330.

[8] B.‐W. Chen , M.‐W. An , K. Wang , et al., “Unprecedented Short‐Circuit Current Density and Efficiency of Vacuum‐Deposited Organic Solar Cells Based on 8H‐thieno[2′,3′:4,5]thieno[3,2‐b] thieno[2,3‐d]pyrrole[2′,3′:4,5]thieno[3,2‐b] thieno[2,3‐d]pyrrole,” Science Bulletin 70 (2025): 897–904, 10.1016/j.scib.2025.01.004. 39827030

[9] X. Che , C.‐L. Chung , C.‐C. Hsu , F. Liu , K.‐T. Wong , and S. R. Forrest , “Donor–Acceptor–Acceptor's Molecules for Vacuum‐Deposited Organic Photovoltaics With Efficiency Exceeding 9%,” Advanced Energy Materials 8, no. 19 (2018): 1703603, 10.1002/aenm.201703603.

[10] B.‐W. Chen , K. Cao , X. Wang , et al., “Design and Performance of Small‐Molecule Donors With Donor–π‐Acceptor Architecture Toward Vacuum‐Deposited Organic Photovoltaics Having Heretofore Highest Short‐Circuit Current Density,” Small 20, no. 43 (2024): 2403486, 10.1002/smll.202403486. 39031678

[11] O. L. Griffith , X. Liu , J. A. Amonoo , et al., “Charge Transport and Exciton Dissociation in Organic Solar Cells Consisting of Dipolar Donors Mixed With C_70_ ,” Physical Review B 92, no. 8 (2015): 085404, 10.1103/PhysRevB.92.085404.

[12] L.‐Y. Lin , Y.‐H. Chen , Z.‐Y. Huang , et al., “A Low‐Energy‐Gap Organic Dye for High‐Performance Small‐Molecule Organic Solar Cells,” Journal of the American Chemical Society 133 (2011): 15822–15825, 10.1021/ja205126t. 21905648

[13] Z. Ning and H. Tian , “Triarylamine: A Promising Core Unit for Efficient Photovoltaic Materials,” Chemical Communications 41 (2009): 5483–5495, 10.1039/b908802d. 19753339

[14] M. Liang and J. Chen , “Arylamine Organic Dyes for Dye‐Sensitized Solar Cells,” Chemical Society Reviews 42 (2013): 3453–3488, 10.1039/c3cs35372a. 23396530

[15] J. Wang , K. Liu , L. Ma , and X. Zhan , “Triarylamine: Versatile Platform for Organic, Dye‐Sensitized, and Perovskite Solar Cells,” Chemical Reviews 116 (2016): 14675–14725, 10.1021/acs.chemrev.6b00432. 27960267

[16] Y.‐H. Chen , L.‐Y. Lin , C.‐W. Lu , et al., “Vacuum‐Deposited Small‐Molecule Organic Solar Cells With High Power Conversion Efficiencies by Judicious Molecular Design and Device Optimization,” Journal of the American Chemical Society 134 (2012): 13616–13623, 10.1021/ja301872s. 22831172

[17] S.‐H. Chou , H.‐W. Kang , S.‐T. Chang , et al., “Cofacial Versus Coplanar Arrangement in Centrosymmetric Packing Dimers of Dipolar Small Molecules: Structural Effects on the Crystallization Behaviors and Optoelectronic Characteristics,” ACS Applied Materials & Interfaces 8 (2016): 18266–18276, 10.1021/acsami.6b03371. 27348150

[18] S.‐W. Chiu , L.‐Y. Lin , H.‐W. Lin , et al., “A Donor–Acceptor–Acceptor Molecule for Vacuum‐Processed Organic Solar Cells With a Power Conversion Efficiency of 6.4%,” Chemical Communications 48, no. 13 (2012): 1857–1859, 10.1039/c2cc16390j. 22167175

[19] H.‐C. Ting , Y.‐H. Chen , L.‐Y. Lin , et al., “Benzochalcogenodiazole‐Based Donor–Acceptor–Acceptor Molecular Donors for Organic Solar Cells,” Chemsuschem 7 (2014): 457–465, 10.1002/cssc.201301090. 24488678

[20] B.‐W. Chen , P.‐Y. Xu , S.‐H. Chen , et al., “Benzotriazole‐Based Donor–Acceptor–Acceptor Electron Donors for Vacuum‐Deposited Small Molecule Organic Solar Cells,” Solar RRL 7, no. 17 (2023): 2300276, 10.1002/solr.202300276.

[21] S.‐H. Chou , H.‐C. Chen , C.‐K. Wang , et al., “Synthesis and Characterization of New Asymmetric thieno[3,4‐b]pyrazine‐based D−π−A−A Type Small Molecular Donors With Near‐Infrared Absorption and Their Photovoltaic Applications,” Organic Electronics 68 (2019): 159–167, 10.1016/j.orgel.2019.02.013.

[22] H.‐C. Ting , Y.‐T. Yang , C.‐H. Chen , et al., “Easy Access to NO_2_‐Containing Donor–Acceptor–Acceptor Electron Donors for High Efficiency Small‐Molecule Organic Solar Cells,” Chemsuschem 9 (2016): 1433–1441, 10.1002/cssc.201600361. 27213296

[23] X. Qi , Y.‐C. Lo , Y. Zhao , et al., “Two Novel Small Molecule Donors and the Applications in Bulk‐Heterojunction Solar Cells,” Frontiers in Chemistry 6 (2018): 260, 10.3389/fchem.2018.00260. PMC6036481 30013968

[24] D. He , F. Zhao , C. Wang , and Y. Lin , “Non‐Radiative Recombination Energy Losses in Non‐Fullerene Organic Solar Cells,” Advanced Functional Materials 32, no. 19 (2022): 2111855, 10.1002/adfm.202111855.

[25] Y. Pan , L. Guo , M. H. Jee , et al., “Polymer Acceptor Copolymerized With Luminescent Unit for High‐Performance All‐Polymer Solar Cells With Low Non‐Radiative Energy Loss,” Advanced Energy Materials 16, no. 3 (2024): 2403747, 10.1002/aenm.202403747.

[26] R. Zhou , Z. Jiang , C. Yang , et al., “All‐Small‐Molecule Organic Solar Cells With Over 14% Efficiency by Optimizing Hierarchical Morphologies,” Nature Communications 10 (2019): 5393, 10.1038/s41467-019-13292-1. PMC6879588 31772169

[27] J. Fu , P. W. K. Fong , H. Liu , et al., “19.31% Binary Organic Solar Cell and Low Non‐Radiative Recombination Enabled by Non‐Monotonic Intermediate State Transition,” Nature Communications 14 (2023): 1760, 10.1038/s41467-023-37526-5. PMC10063688 36997533

[28] H. Lu , W. Liu , G. Ran , et al., “High‐Efficiency Binary and Ternary Organic Solar Cells Based on Novel Nonfused‐Ring Electron Acceptors,” Advanced Materials 36, no. 7 (2023): 2307292, 10.1002/adma.202307292. 37811717

[29] Y. Zhang , G. Cai , Y. Li , et al., “An Electron Acceptor Analogue for Lowering Trap Density in Organic Solar Cells,” Advanced Materials 33, no. 14 (2021): 2008134, 10.1002/adma.202008134. 33656774

[30] Y. Jiang , S. Sun , R. Xu , et al., “Non‐Fullerene Acceptor With Asymmetric Structure and Phenyl‐Substituted Alkyl Side Chain for 20.2% Efficiency Organic Solar Cells,” Nature Energy 9 (2024): 975–986, 10.1038/s41560-024-01557-z.

