# Supplementary material for: Theretofore Highest Efficiency in Vacuum‐Deposited Organic Solar Cells Originating From Triarylamine‐Based Small‐Molecule Donors Containing Fused Heterocycle Units
Source: Adv Sci (Weinh). 2026 Jul 13:e76486. Online ahead of print. doi: 10.1002/advs.76486 (PMC13360112; doi:10.1002/advs.76486)
Supplement: Supplementary file 1 — Supporting File: advs76486‐sup‐0001‐SuppMat.docx. [file ADVS-9999-e76486-s001.docx]

Supporting Information

Theretofore Highest Efficiency in Vacuum-Deposited Organic Solar Cells Originating from Triarylamine-Based Small-Molecule Donors Containing Fused Heterocycle Units

Yun-Fei Li, Le-Shan Dai, Zuo-Chang Chen, Shi-Long Xiong, Bin-Wen Chen*, Ke-Yue Yang , Qiu Xiong, Yi-Dong Yan, Kun Cao, Da-Qin Yun, Feng He*, Lin-Long Deng*, Su-Yuan Xie, and Lan-Sun Zheng

Y.F Li, L.S. Dai. Z.C. Chen, B.W. Chen, K.Y. Yang, Q. Xiong, K. Cao, S.Y. Xie, L.S. Zheng

State Key Laboratory for Physical Chemistry of Solid Surfaces, Collaborative Innovation Center of Chemistry for Energy Materials, Department of Chemistry, College of Chemistry and Chemical Engineering, Xiamen University, Xiamen 361005, China.

E-mail: bwchen@xmu.edu.cn

Y.D. Yan, L.L. Deng

Pen-Tung Sah Institute of Micro-Nano Science and Technology, Xiamen University, Xiamen 361005, China.

E-mail: denglinlong@xmu.edu.cn

S.L. Xiong, F. He

Shenzhen Grubbs Institute and Department of Chemistry, Southern University of Science and Technology, Shenzhen 518055, China.

E-mail: hef@sustech.edu.cn

D.Q. Yun

College of Energy, Xiamen University, Xiamen 361005, China.

Yun-Fei Li and Le-Shan Dai contributed equally to this work.

**Experimental procedures.**

**Materials.** Unless otherwise specified, all chemical compounds and reagents used in this work were purchased from J&K, Bide Pharm, Sigma-Aldrich, or Alfa-Aesar and were used as received without further purification. Transparent conducting glass substrates, coated with indium tin oxide (ITO, 15 Ω sq^-1^) were procured from Advanced Electrode Technology Limited. The small-molecule donor materials, including PF, PT, BF, and BT were synthesized in the laboratory and subsequently purified via vacuum sublimation. Fullerene C_70_ was obtained from Funano Co., Ltd. MoO_x_ and BCP were purchased from Xi'an Polymer Light Technology Corporation. Ag with high purity (>99.999%) was procured from ZhongNuo Advanced Material (Beijing) Technology Co., Ltd.

**Device fabrication.** Prior to deposition, the patterned ITO substrates were cleaned by sequential ultrasonic treatments in detergent, deionized water, acetone, and isopropyl alcohol for 15 min. Subsequently, the ITO substrates were treated in a UV ozone chamber for 10 min before transferring into a nitrogen-filled glove box. Vacuum-deposited organic solar cells were fabricated with the structure of ITO/MoO_x_ (10 nm)/Donor: C_70_ (1:2 volume ratio, 80 nm)/BCP (5 nm)/Ag (100 nm). The deposited rate and thickness of the devices were monitored using an in situ quartz crystal monitor, and calibrated by a surface profiler (Dektak XT). The hole extraction layer of MoO_x_ with the thickness of 10 nm was deposited on the ITO substrates at ~ 0.15 Å/s in a high vacuum chamber with a base pressure lower than 1.0×10^-6^ Torr. The active layer was prepared by co-deposition of the small molecule donors and C_70_ acceptor. The BCP exciton-blocking layer with the thickness of 5 nm was subsequently deposited at the rates of 0.1-0.20 Å/s. Finally, Ag cathode with the thickness 120 nm was deposited at the rates of 1.0-1.2 Å/s to complete the device.

**Characterization.** ^1^H NMR and ^13^C NMR spectra were carried out on a Bruker Biospin Advance III spectrometer at 500 or 850 MHz. Chemical shifts were reported in ppm using CDCl_3_ as internal reference for ^1^H and ^13^C NMR spectra. The high-resolution time-of-flight mass spectrometry measurements were performed on a Bruker autoflex maX MALDI-TOF mass spectrometer with TCNQ as the matrix. The absorption spectra of the small-molecule donors in dichloromethane solutions and in thin films were tested using a Varian Cary 5000 UV-Vis spectrophotometer. Cyclic voltammetry (CV) tests were conducted on a Shanghai Chenhua CHI-660E electrochemical workstation. Measurements were carried out in a one-compartment cell equipped with a platinum disc as the working electrode, a platinum wire as the counter electrode, and a Ag/Ag^+^ electrode (0.01 M AgNO_3_, 0.09 M tetrabutylammonium hexafluorophosphate (Bu_4_NPF_6_) in acetonitrile) as the reference electrode. The oxidation processes were measured in anhydrous CH_2_Cl_2_ containing 0.1 M tetrabutylammonium hexafluorophosphate (TBAPF_6)_ as the supporting electrolyte, while the reduction processes were studied in anhydrous tetrahydrofuran containing 0.1 M tetrabutylammonium perchlorate (TBAP) as the supporting electrolyte. All potentials were referenced to the ferrocene/ferrocenium (Fc/Fc^+^) couple. The current density and voltage (*J*-*V*) characteristics were measured by using a 2400 SourceMeter Keithley under the illumination of AM 1.5G solar light from a 300 W xenon solar simulator (Newport Oriel Solar Simulators). The incident light intensity was calibrated to 100 mW cm^-2^ by using a National Renewable Energy Laboratory (NREL) traceable reference Si photodiode with a KG5 filter. The external quantum efficiency (EQE) was measured by Merlin lock-in amplifier coupled with a CS260 monochromator and 300 W xenon lamp. The hole and electron mobilities were measured in the dark by the space charge limited current (SCLC) method on a computer-controlled Keithley 2400 source meter. The structure of electron-only devices was ITO/ZnO/Donor: C_70_/PNDIT-F3N/Al, while the hole-only devices were fabricated with the structure of ITO/MoO_x_/Donor: C_70_/MoO_x_/Ag. The thickness of Donor: C_70_ active layer is around 100 nm across all donor systems. Two-dimensional grazing incidence wide-angle X-ray scattering (2D-GIWAXS) measurements were performed with a Xeuss 2.0 SAXS/WAXS laboratory beamline using a Cu X-ray source (8.05 keV, 1.54 Å) and a Pilatus3R 300 K detector. The samples for GIWAXS measurements are fabricated on silicon substrates using the same recipe for the devices. The surface morphology of the active layers was obtained by atomic force microscope (AFM, Multimode 8, Veeco Instruments, Inc.). Transmission electron microscopy (TEM) images were acquired on a FEI Tecnai F20 instrument with an accelerating voltage of 200 kV. EQE_EL_ measurements were performed by applying external voltage/current sources through the devices (ELCT-3010, Enlitech).

**Synthesis and separation details.**

**
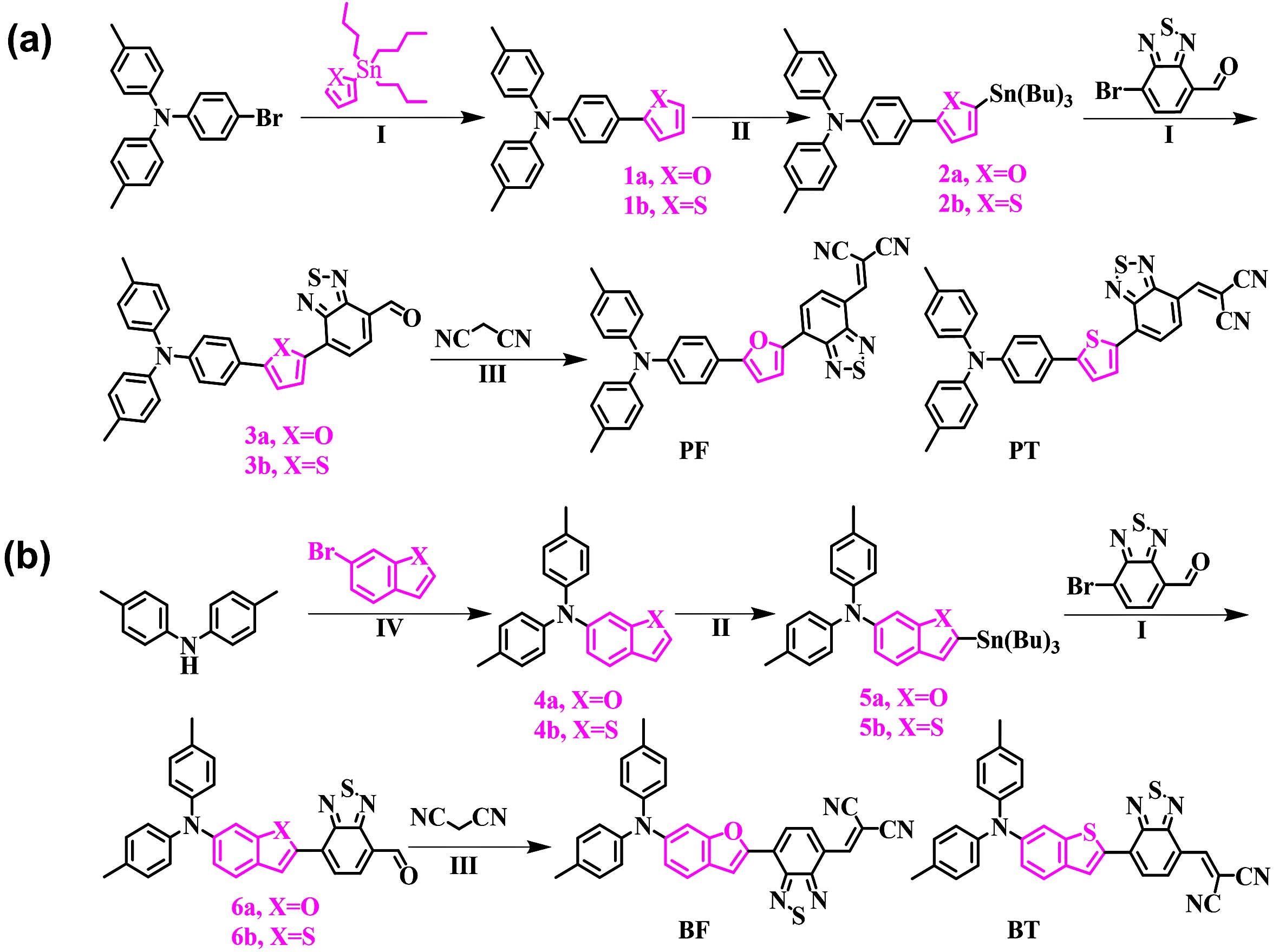
**

(Ⅰ) PdCl₂(PPh₃)₂, N_2_, PhMe, 110 °C, (Ⅱ) nBuLi, Sn(Bu)_3_Cl, THF, N_2_, -78 °C-rt, (Ⅲ) Al_2_O_3_, PhMe, 70 °C, (Ⅳ) Pd(OAc)_2_, RuPhos, NaOt-Bu, PhMe, N_2_, 110 °C.

**Scheme S1.** Synthetic routes of PF, PT, BF, and BT.

*Synthesis of 4-(furan-2-yl)-N,N-bis(p-tolyl)aniline* (**1a**) *and 4-Methyl-N-(4-methylphenyl)-N- [4-(2-thienyl)phenyl]benzenamine* (**1b**): (4-bromophenyl)-bis(p-tolyl)aniline (1.05 g, 6.00 mmol), 2-(tributyltin) furan (2.27 mL, 7.20 mmol) or 2-(tributyltin) thiophene (1.14 mL, 7.20 mmol) and PdCl₂(PPh₃)₂ (105 mg, 0.30 mmol) were dissolved in 80 mL degassed toluene. Subsequently, the reaction mixture was refluxed at 110 °C under a nitrogen atmosphere for 13-15 hours. Upon completion, the solvent was removed under vacuum. The crude product was subsequently purified by silica gel chromatography using petroleum ether as eluent to afford **1a** (1.61 g, 79%) and **1b** (1.81 g, 85%).

**1a**：^1^H NMR (500 MHz, CDCl_3_): δ 7.51-7.49 (m, 2H), 7.42 (br s, 1H), 7.08 (d, *J* = 5.0 Hz, 4H), 7.02 (t, *J* = 10.0 Hz, 6H), 6.52 (d, *J* = 5.0 Hz, 1H), 6.45 (d, *J* = 5.0 Hz, 1H), 2.32 (s, 6H). ^13^C NMR (125 MHz, CDCl_3_): δ 154.15, 147.47, 145.09, 141.37, 132.64, 130.17, 129.89, 124.66, 124.63, 122.48, 111.53, 103.50, 20.80.

**1b**：^1^H NMR (500 MHz, CDCl_3_): δ 7.48 (d, *J* = 10.0 Hz, 2H), 7.23 (t, *J* = 5.0 Hz, 2H), 7.13-7.10 (m, 4H), 7.08-7.04 (m, 7H), 2.36 (s, 6H). The 1b related structure has been reported in the literature^[1-2]^.

*Synthesis of 4-methyl-N-(p-tolyl)-N-(4-(5-(tributylstannyl)furan-2-yl)phenyl)aniline* (**2a**) *and* *4-methyl-N-(p-tolyl)-N-(4-(5-(tributylstannyl)thiophen-2-yl)phenyl)aniline* (**2b**): 1a (1.36 g, 4.00 mmol) or 1b (1.04 g, 2.70 mmol) was dissolved in THF (100 mL) and cooled to -78 °C under N_2_ atmosphere. Subsequently, n-BuLi (2.55 mL, 4.08 mmol or 1.72 mL, 2.75 mmol, 1.6M) was added dropwise, and the mixture was stirred at -78 °C for one hour. Tri-n-butyltin chloride (1.22 mL, 4.16 mmol or 0.76 mL, 2.81 mmol) was added, and the mixture was stirred at -78 °C for one hour. The reaction mixture was allowed to warm to room temperature and stirred overnight. The reaction mixture was quenched with water and extracted with petroleum ether, and the solvent was removed under reduced pressure. The crude product, **2a** or **2b**, was used directly in the subsequent step without further purification.

*Synthesis of 7-(5-(4-(di-p-tolylamino)phenyl)furan-2-yl)benzo[c][1,2,5]thiadiazole- 4- carbaldehyde* (**3a**) *and* *7-(5-(4-(di-p-tolylamino)phenyl)thiophen-2-yl)benzo[c][1,2,5] thiadiazole- 4-carbaldehyde* (**3b**): 2a (4.00 mmol) or 2b (2.70 mmol), 7-bromo-benzo[c][1,2,5]thiadiazole-4- carbaldehyde (1.40 g, 4.80 mmol or 950 mg, 3.28 mmol) and PdCl₂(PPh₃)₂ (141 mg, 0.20 mmol or 96 mg, 0.14 mmol) were dissolved in 80 mL degassed toluene. Subsequently, the reaction mixture was refluxed at 110 °C under a nitrogen atmosphere for 14 hours. Upon completion, the solvent was removed under vacuum. The crude product was subsequently purified by silica gel chromatography using dichloromethane/petroleum ether (v/v, 1:3) as eluent to afford **3a** (1.52 g, 76%) and **3b** (1.16 g, 83%).

**3a**：^1^H NMR (500 MHz, CDCl_3_): δ 10.67 (s, 1H), 8.24 (d, *J* = 5.0 Hz, 1H), 8.09 (br s, 1H), 8.00 (d, *J* = 5.0 Hz, 1H), 7.64 (d, *J* = 10.0 Hz, 2H), 7.12 (d, *J* = 10.0 Hz, 4H), 7.04 (br s, 6H), 6.74 (s, 1H), 2.37 (s, 6H). ^13^C NMR (212.5 MHz, CDCl_3_): δ 188.44, 153.62, 151.02, 133.51, 133.13, 129.92, 125.33, 124.81, 121.71, 120.86, 119.02, 122.48, 20.79 (29.67 ppm from impurity).

**3b**：^1^H NMR (500 MHz, CDCl_3_): δ 10.68 (s, 1H), 8.27 (d, *J* = 5.0 Hz, 1H), 8.18 (d, *J* = 5.0 Hz, 1H), 7.92 (d, *J* = 5.0 Hz, 1H), 7.51 (d, *J* = 10.0 Hz, 2H), 7.30 (br s, 1H), 7.11 (d, *J* = 10.0 Hz, 4H), 7.04 (t, *J* = 7.5 Hz, 6H), 2.35 (s, 6H). ^13^C NMR (125 MHz, CDCl_3_): δ 188.38, 153.77, 152.22, 133.30, 133.13, 133.11, 132.73, 131.66, 130.00, 126.60, 125.14, 125.00, 123.28, 123.14, 121.70, 20.83.

*Synthesis of 2-((7-(5-(4-(di-p-tolylamino)phenyl)furan-2-yl)benzo[c][1,2,5]thiadiazol-4 -yl) methylene)malononitrile* (**PF**): 3a (932 mg, 1.86 mmol), malononitrile (246 mg, 3.72 mmol), and Al_2_O_3_ (930 mg, 9.12 mmol) were dissolved in toluene (60 mL). Subsequently, the reaction mixture was stirred at 70 °C overnight. Upon completion, the solvent was removed under vacuum. The crude product was subsequently purified by silica gel chromatography using dichloromethane/ petroleum ether (v/v, 1:4) as eluent to afford **PF** (848 mg, 83%).

**PF**：^1^H NMR (500 MHz, CDCl_3_): δ 8.82-8.78 (m, 2H), 8.14 (d, *J* = 10.0 Hz, 1H), 8.06 (d, *J* = 5.0 Hz, 1H), 7.65 (d, *J* = 10.0 Hz, 2H), 7.13-7.04 (m, 10H), 2.34 (s, 6H). ^13^C NMR can^’^t be recorded due to the low solubility of **PF**. MALDI-TOF MS (m/z): calculated. for (C_34_H_23_N_5_OS): 549.16; Found: 548.86.

*Synthesis of 2-((7-(5-(4-(di-p-tolylamino)phenyl)thiophen-2-yl)benzo[c][1,2,5]thiadiazol-4-yl) methylene)malononitrile* (**PT**): 3b (962 mg, 1.86 mmol), malononitrile (246 mg, 3.72 mmol), and Al_2_O_3_ (930 mg, 9.12 mmol) were dissolved in toluene (60 mL). Subsequently, the reaction mixture was stirred at 70 °C overnight. Upon completion, the solvent was removed under vacuum. The crude product was purified by silica gel chromatography using dichloromethane/petroleum ether (v/v, 1:4) as eluent to afford **PT** (925 mg, 88%).

**PT**：^1^H NMR (500 MHz, CDCl_3_): δ 8.77-8.74 (m, 2H), 8.33 (d, *J* = 5.0 Hz, 1H), 7.96 (d, *J* = 10.0 Hz, 1H), 7.53 (d, *J* = 10.0 Hz, 2H), 7.36 (d, *J* = 5.0 Hz, 1H), 7.11 (d, *J* = 10.0 Hz, 4H), 7.06-7.04(m, 6H), 2.34 (s, 6H). ^13^C NMR (125 MHz, CDCl_3_): δ 154.49, 152.18, 151.11, 150.46, 148.97, 144.55, 136.16, 133.51, 133.46, 132.60, 130.83, 130.07, 126.76, 125.82, 125.25, 123.60, 123.50, 121.46, 121.03, 114.12, 113.33, 81.39, 20.87. MALDI-TOF MS (m/z): calculated. for (C_34_H_23_N_5_S_2_): 565.14; Found: 565.59.

*Synthesis of N,N-di-p-tolylbenzofuran-6-amine* (**4a**) *and N,N-di-p-tolylbenzo[b]thiophen-6-amine* (**4b**): 6-bromozofuran (0.68 mL, 5.48 mmol) or 6-bromobenzothiophene (1.17 g, 5.48 mmol), di-p-tolylamine (982 mg, 4.98 mmol), RuPhos (46 mg, 0.10 mmol), Pd(OAc)₂ (11.2 mg, 0.05 mmol) and NaOt-Bu (574 mg, 5.98 mmol) were dissolved in 80 mL degassed toluene. The reaction mixture was refluxed at 110 °C under a nitrogen atmosphere for 12 hours. Upon completion, the solvent was removed under vacuum. The crude product was purified by silica gel chromatography using petroleum ether as eluent to afford **4a** (1.10 g, 70%) and **4b** (1.20 g, 73%).

**4a**：^1^H NMR (500 MHz, CDCl_3_): δ 7.54 (d, *J* = 5.0 Hz, 1H), 7.41 (d, *J* = 5.0 Hz, 1H), 7.19 (br s, 1H), 7.07 (d, *J* = 5.0 Hz, 4H), 7.02-6.99 (m, 5H), 6.70 (d, *J* = 5.0 Hz, 1H), 2.32 (s, 6H). ^13^C NMR (125 MHz, CDCl_3_): δ 155.85, 145.76, 144.51, 132.09, 129.80, 124.11, 122.30, 120.97, 120.02, 106.78, 106.38, 20.76.

**4b**：^1^H NMR (500 MHz, CDCl_3_): δ 7.63 (d, *J* = 10.0 Hz, 1H), 7.49 (d, *J* = 5.0 Hz, 1H), 7.27 (d, 1H, overlapped with CDCl_3_), 7.23 (d, *J* = 5.0 Hz, 1H), 7.11 (d, *J* = 5.0 Hz, 1H), 7.06 (d, *J* = 10.0 Hz, 4H), 7.00 (d, *J* = 5.0 Hz, 4H), 2.31 (s, 6H). ^13^C NMR (125 MHz, CDCl_3_): δ 145.61, 145.40, 140.89, 134.74, 132.28, 129.85, 127.62, 126.94, 126.90, 124.96, 124.65, 124.52, 124.27, 123.73, 123.54, 123.45, 121.58, 116.47, 20.78.

*Synthesis of N,N-di-p-tolyl-2-(tributylstannyl)benzofuran-6-amine* (**5a**) *and* *N,N-di-p-tolyl-2-*

*(tributylstannyl)benzo[b]thiophen-6-amine* (**5b**): 4a (780 mg, 2.49 mmol) or 4b (820 mg, 2.49 mmol) was dissolved in THF (60 mL) and cooled to -78 °C. Subsequently, n-butyllithium (1.10 mL, 2.49 mmol, 1.6 M) was added dropwise, and the mixture was stirred at -78 °C for one hour. Tri-n-butyltin chloride (0.74 mL, 2.74 mmol) was added, and the mixture was stirred at -78 °C for one hour. The reaction mixture was allowed to warm to room temperature and stirred overnight. The reaction mixture was quenched with water and extracted with petroleum ether, and the solvent was removed under reduced pressure. The crude product, **5a** or **5b**, was used directly in the subsequent step without further purification.

*Synthesis of 7-(6-(di-p-tolylamino)benzofuran-2-yl)benzo[c][1,2,5]thiadiazole-4-carbaldehyde* (**6a**) *and* *7-(6-(di-p-tolylamino)benzo[b]thiophen-2-yl)benzo[c][1,2,5]thiadiazole-4-carbaldehyde* (**6b**): 5a (2.49 mmol) or 5b (2.49 mmol), 7-bromo-benzo[c][1,2,5]thiadiazole-4-carbaldehyde (727 mg, 2.99 mmol) and PdCl₂(PPh₃)₂ (88.4 mg, 0.12 mmol) were dissolved in 60 mL degassed toluene. Subsequently, the reaction mixture was refluxed at 110 °C under a nitrogen atmosphere for 13 hours. Upon completion, the solvent was removed under vacuum. The crude product was purified by silica gel chromatography using dichloromethane/petroleum ether (v/v, 1:3) as eluent to afford **6a** (958 mg, 81%) and **6b** (1.04 g, 85%).

**6a**：^1^H NMR (500 MHz, CDCl_3_): δ 10.71 (s, 1H), 8.26-8.24 (m, 2H), 8.19 (d, *J* = 10.0 Hz, 1H), 7.49 (d, *J* = 10.0 Hz, 1H), 7.16 (br s, 1H), 7.11 (d, *J* = 10.0 Hz, 4H),, 7.06 (d, *J* = 10.0 Hz, 4H), 7.02 (dd, *J* = 5.0, 10.0 Hz, 1H), 2.34 (s, 6H). ^13^C NMR (125 MHz, CDCl_3_): δ 188.57, 156.56, 153.67, 151.40, 150.08, 148.25, 145.10, 133.26, 132.92, 130.05, 128.51, 125.58, 125.11, 123.48, 122.48, 122.16, 119.37, 112.84, 104.13, 20.86.

**6b**：^1^H NMR (500 MHz, CDCl_3_): δ 10.73 (s, 1H), 8.68 (s, 1H), 8.22 (d, *J* = 10.0 Hz, 1H), 7.94 (d, *J* = 5.0 Hz, 1H), 7.70 (d, *J* = 10.0 Hz, 1H), 7.42 (s, 1H), 7.12-7.10 (m, 5H), 7.06 (d, *J* = 10.0 Hz, 4H), 2.35 (s, 6H). ^13^C NMR (125 MHz, CDCl_3_): δ 188.50, 153.86, 152.58, 144.94, 134.97, 133.42, 133.36, 132.61, 130.08, 128.36, 125.55, 125.15, 124.80, 121.00, 113.87, 20.86.

*Synthesis of 2-((7-(6-(di-p-tolylamino)benzofuran-2-yl)benzo[c][1,2,5]thiadiazol-4-yl) methylene)malononitrile* (**BF**): 6a (950 mg, 2.00 mmol), malononitrile (267 mg, 4.00 mmol), and Al_2_O_3_ (1.01 g, 9.98 mmol) were dissolved in toluene (60 mL). Subsequently, the reaction mixture was stirred at 70 °C overnight. Upon completion, the solvent was removed under vacuum. The crude product was purified by silica gel chromatography using dichloromethane/petroleum ether (v/v, 1:4) as eluent to afford **BF** (889 mg, 85%).

**BF**：^1^H NMR (500 MHz, CDCl_3_): δ 8.78-8.76 (m, 2H), 8.26 (s, 1H), 8.15 (d, *J* = 10.0 Hz, 1H), 7.51 (d, *J* = 10.0 Hz, 1H), 7.14-7.00 (m, 10H), 2.36 (s, 6H). ^13^C NMR (150 MHz, CDCl_3_): δ 156.94, 154.31, 152.17, 150.19, 144.85, 133.67, 130.87, 130.13, 128.46, 125.39, 123.36, 122.34, 121.66, 119.31, 114.08, 113.26, 103.52, 81.78, 21.04. MALDI-TOF MS (m/z): calculated. for (C_32_H_21_N_5_OS): 523.15; Found: 522.74.

*Synthesis of 2-((7-(6-(di-p-tolylamino)benzo[b]thiophen-2-yl)benzo[c][1,2,5]thiadiazol-4-yl) methylene)malononitrile* (**BT**): 6b (982 mg, 2.00 mmol), malononitrile (267 mg, 4.00 mmol), and Al_2_O_3_ (1.01 g, 9.98 mmol) were dissolved in toluene (60 mL). Subsequently, the reaction mixture was stirred at 70 °C overnight. Upon completion, the solvent was removed under vacuum. The crude product was purified by silica gel chromatography using dichloromethane/ petroleum ether (v/v, 1:3) as eluent to afford **BT** (938 mg, 87%).

**BT**：^1^H NMR (500 MHz, CDCl_3_): δ 8.77-8.70 (m, 3H), 7.90 (d, *J* = 10.0 Hz, 1H), 7.70 (d, *J* = 10.0 Hz, 1H), 7.37 (br s, 1H), 7.14-7.06 (m, 9H), 2.37 (s, 6H), (5.3 ppm from dichloromethane). ^13^C NMR (150 MHz, CDCl_3_): δ 154.38, 152.14, 151.32, 147.83, 144.66, 142.58, 135.57, 134.71, 133.72, 130.65, 130.14, 129.38, 125.45, 125.39, 124.95, 121.53, 120.73, 113.99, 113.19, 81.86, 20.89. MALDI-TOF MS (m/z): calculated. for (C_32_H_21_N_5_S_2_): 539.12; Found: 538.65.

**
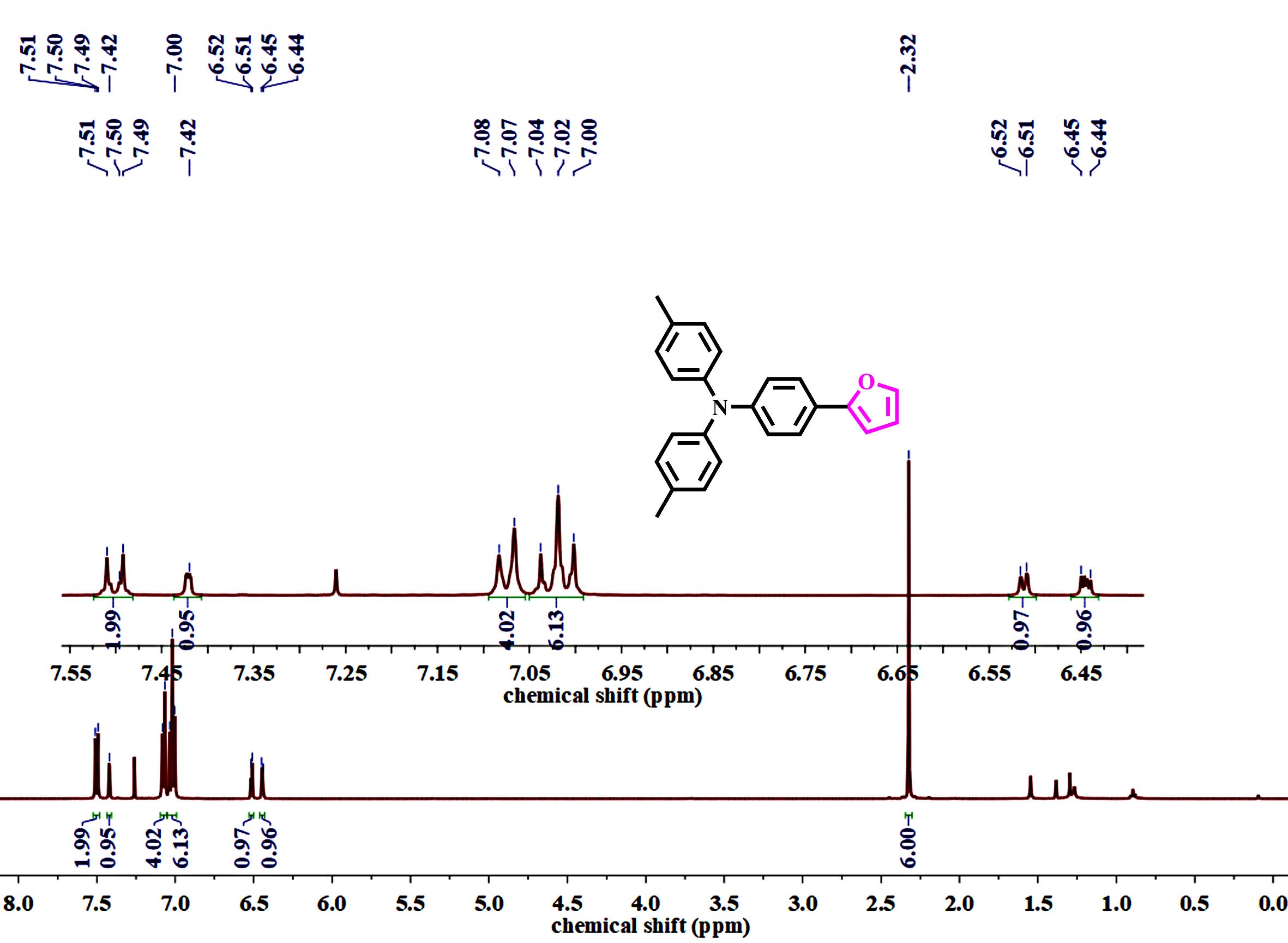
**

**Figure S1.** ^1^H NMR spectrum of **1a**.

**
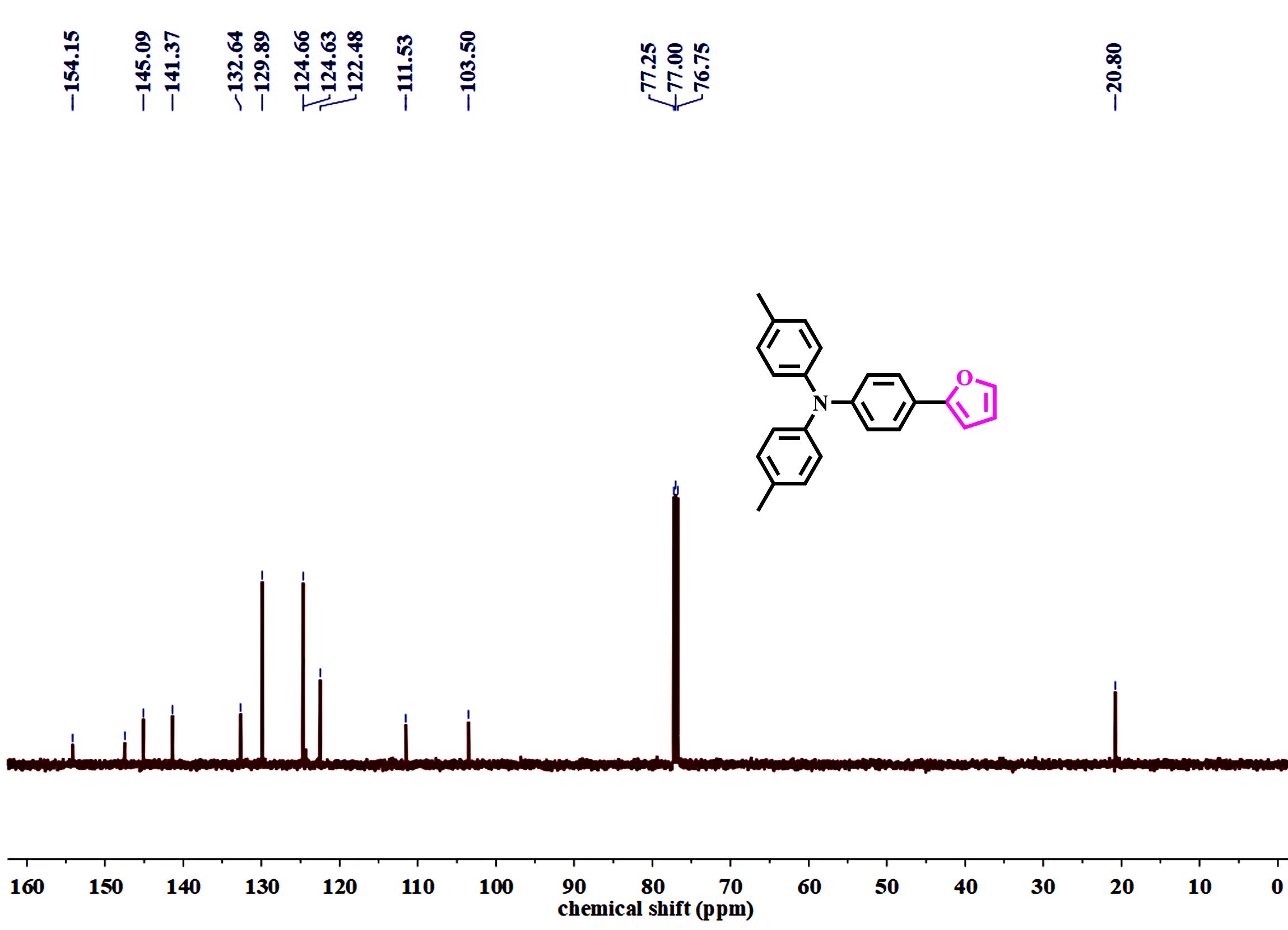
**

**Figure** **S2.** ^13^C NMR spectrum of **1a**.

**
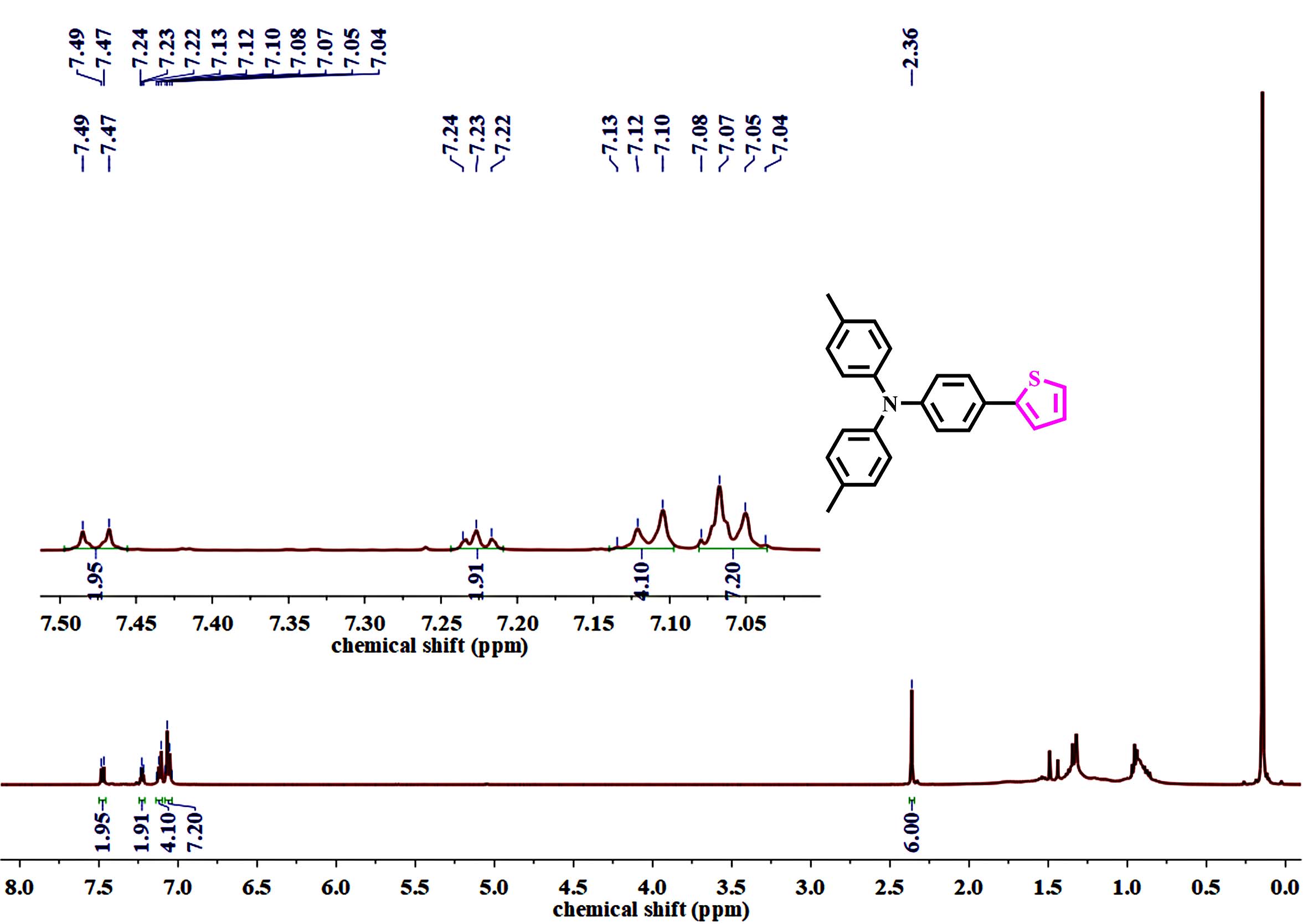
**

**Figure** **S3.** ^1^H NMR spectrum of **1b**.

**
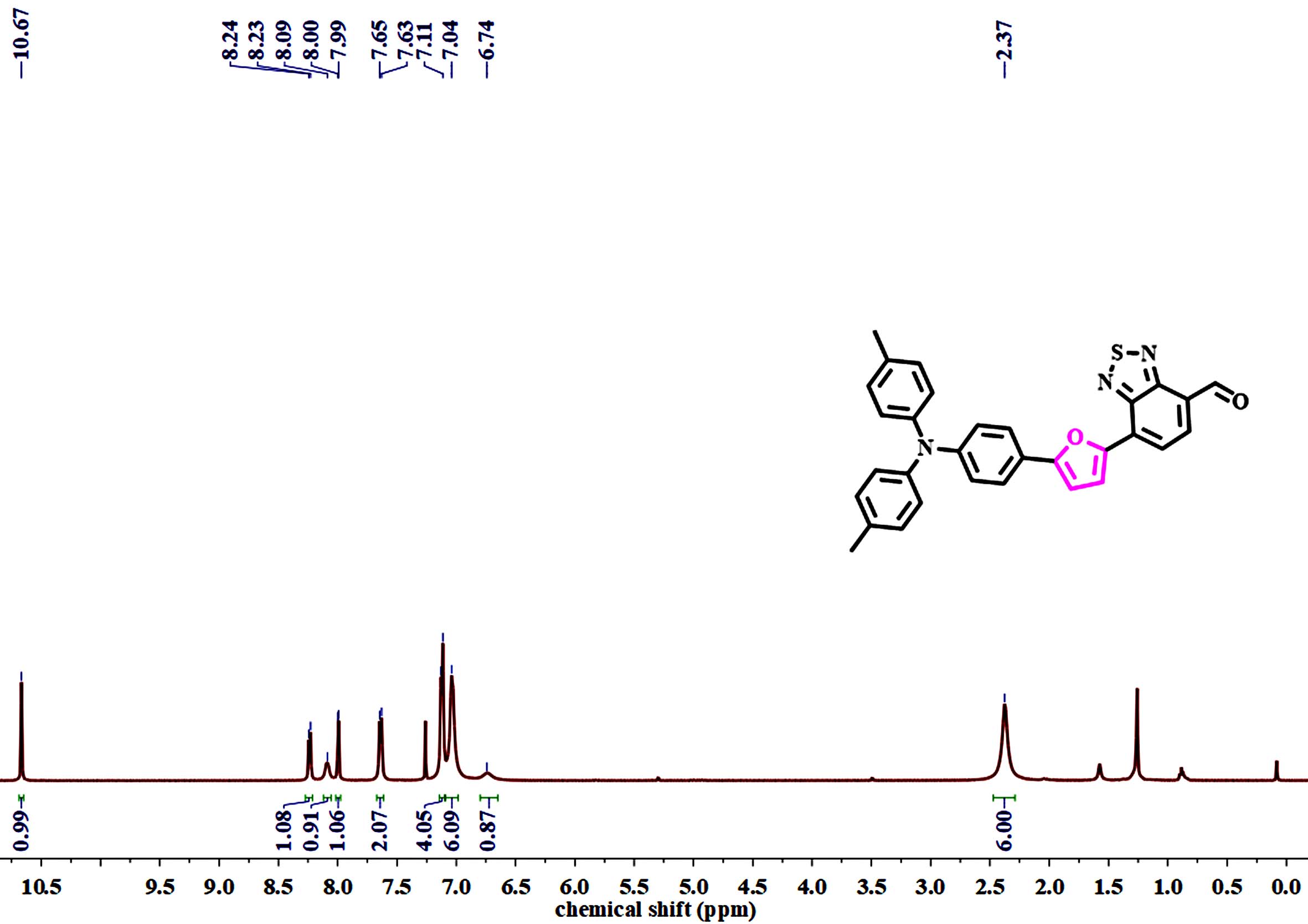
**

**Figure** **S4.** ^1^H NMR spectrum of **3a**.

**
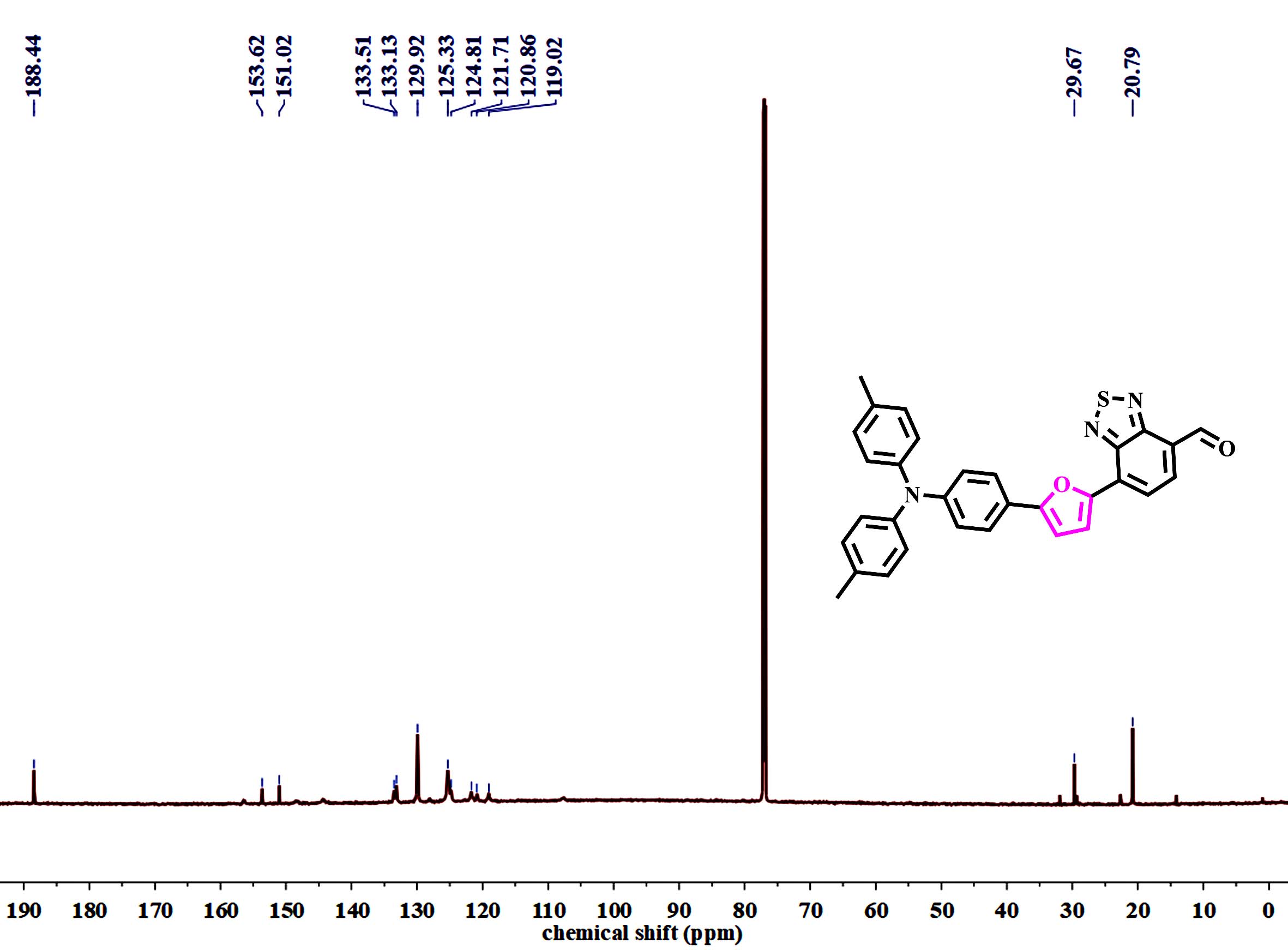
**

**Figure** **S5.** ^13^C NMR spectrum of **3a**.

**
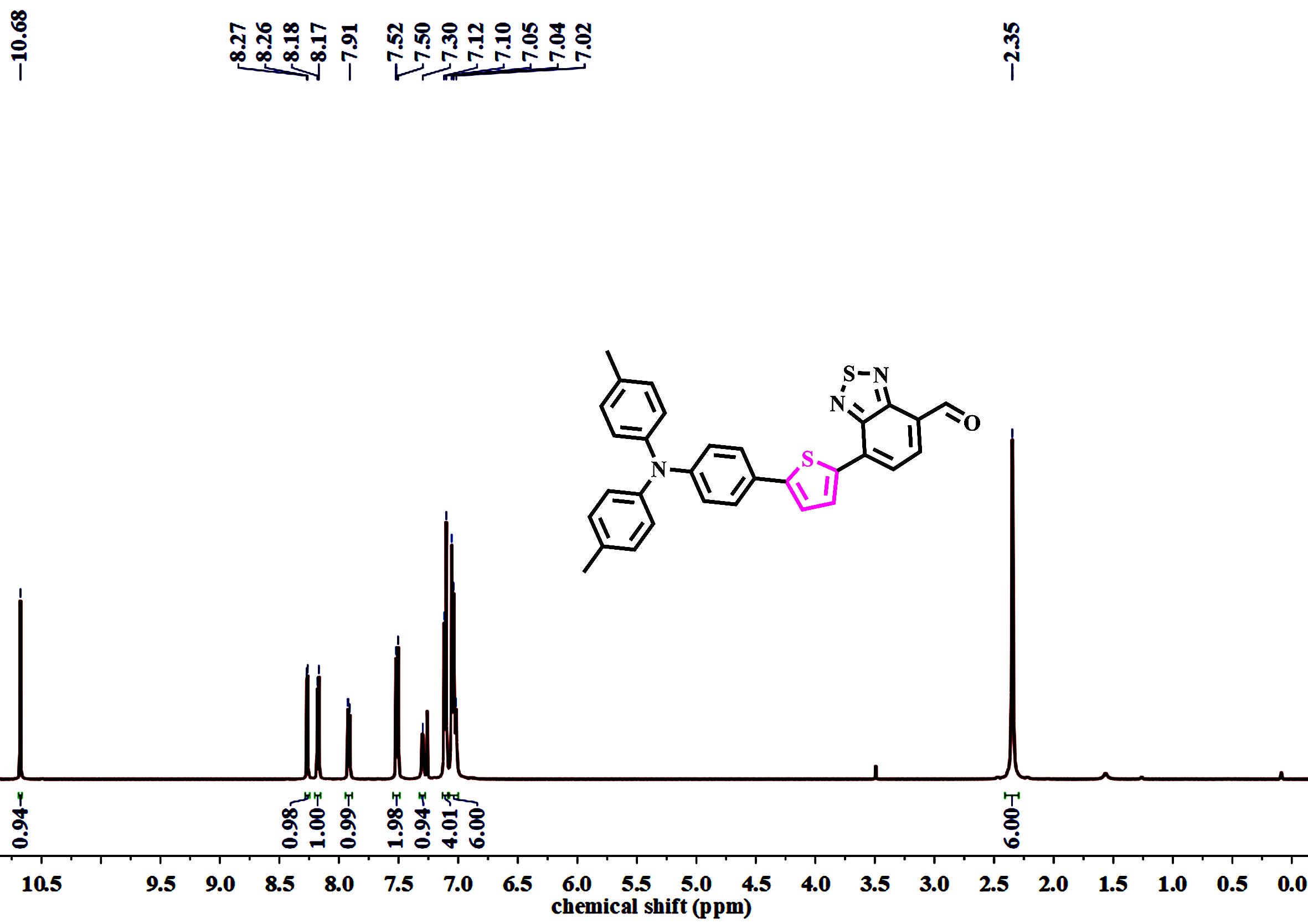
**

**Figure** **S6.** ^1^H NMR spectrum of **3b**.

**
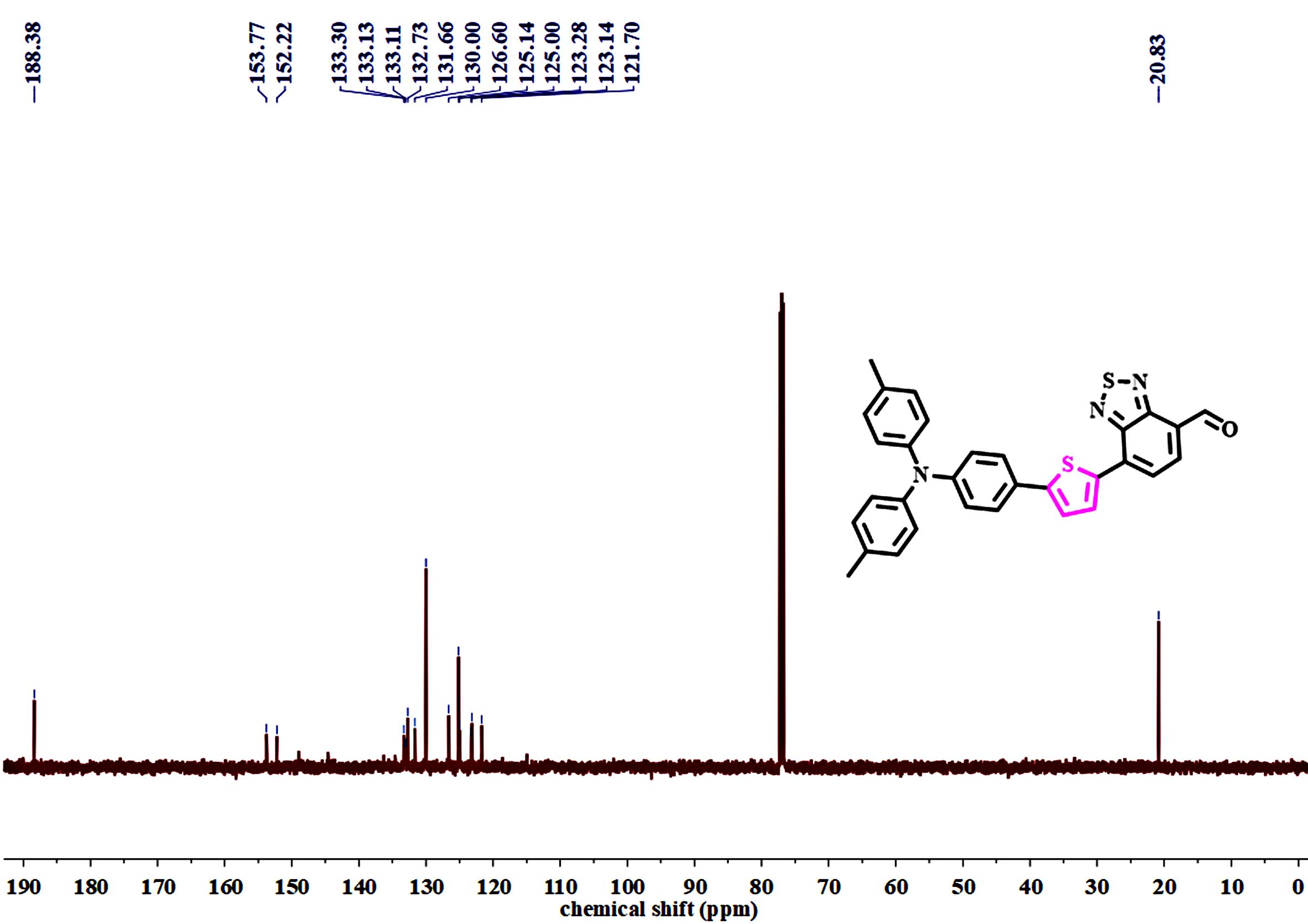
**

**Figure** **S7.** ^13^C NMR spectrum of **3b**.

**
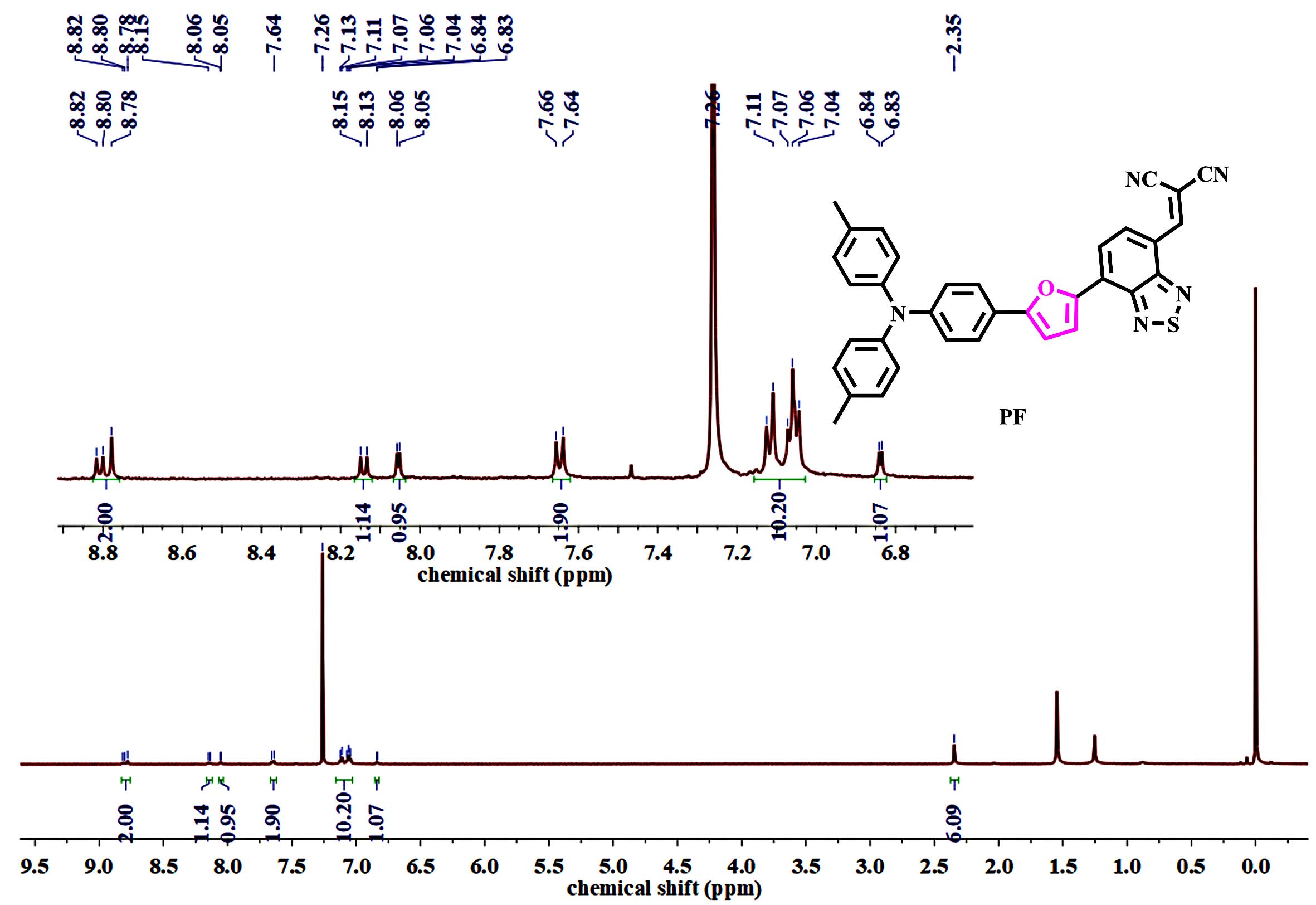
**

**Figure** **S8.** ^1^H NMR spectrum of **PF**.

**
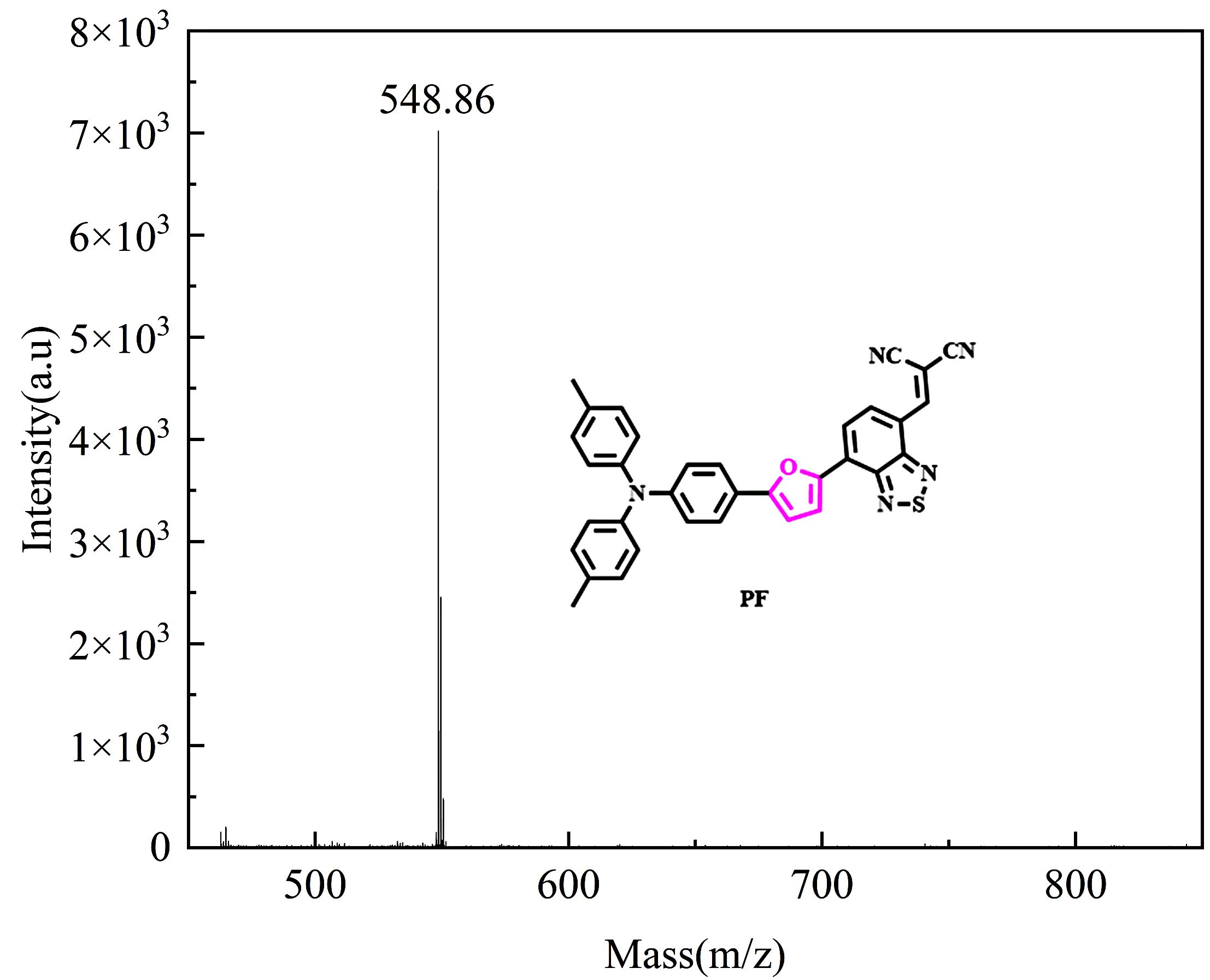
**

**Figure** **S9.** MALDI-TOF MS spectrum of **PF**.

**
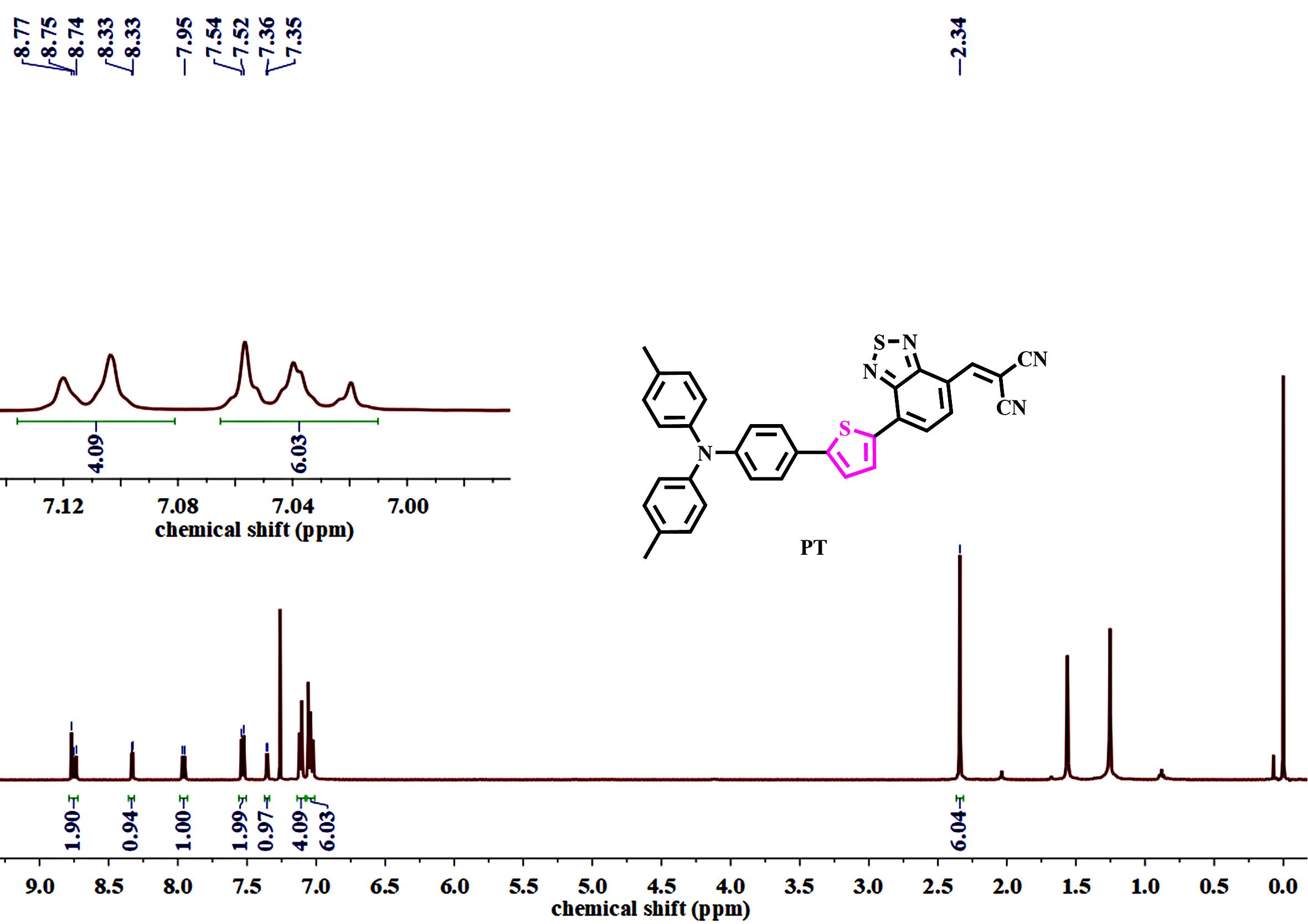
**

**Figure** **S10.** ^1^H NMR spectrum of **PT**.


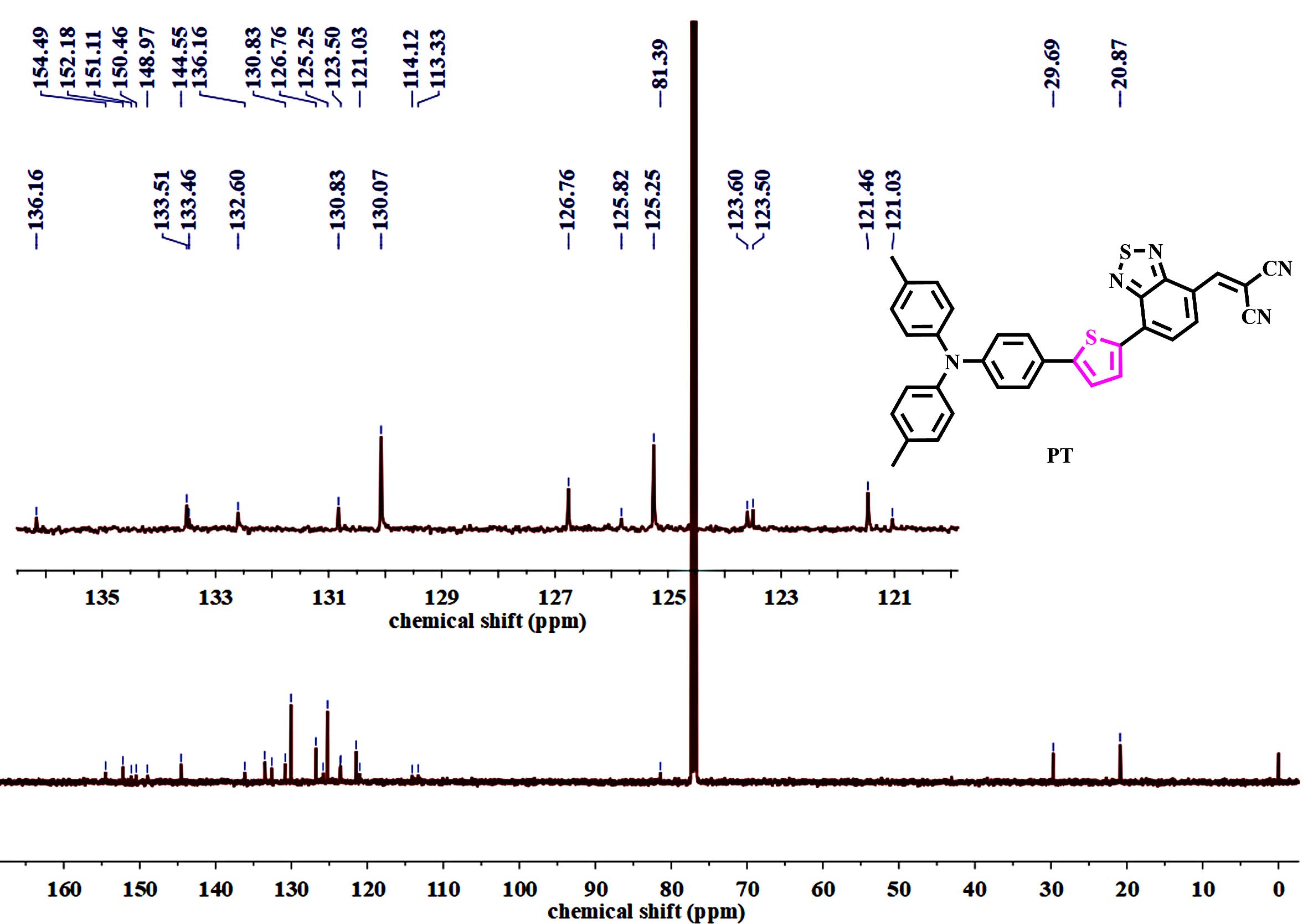


**Figure** **S11.** ^13^C NMR spectrum of **PT**.


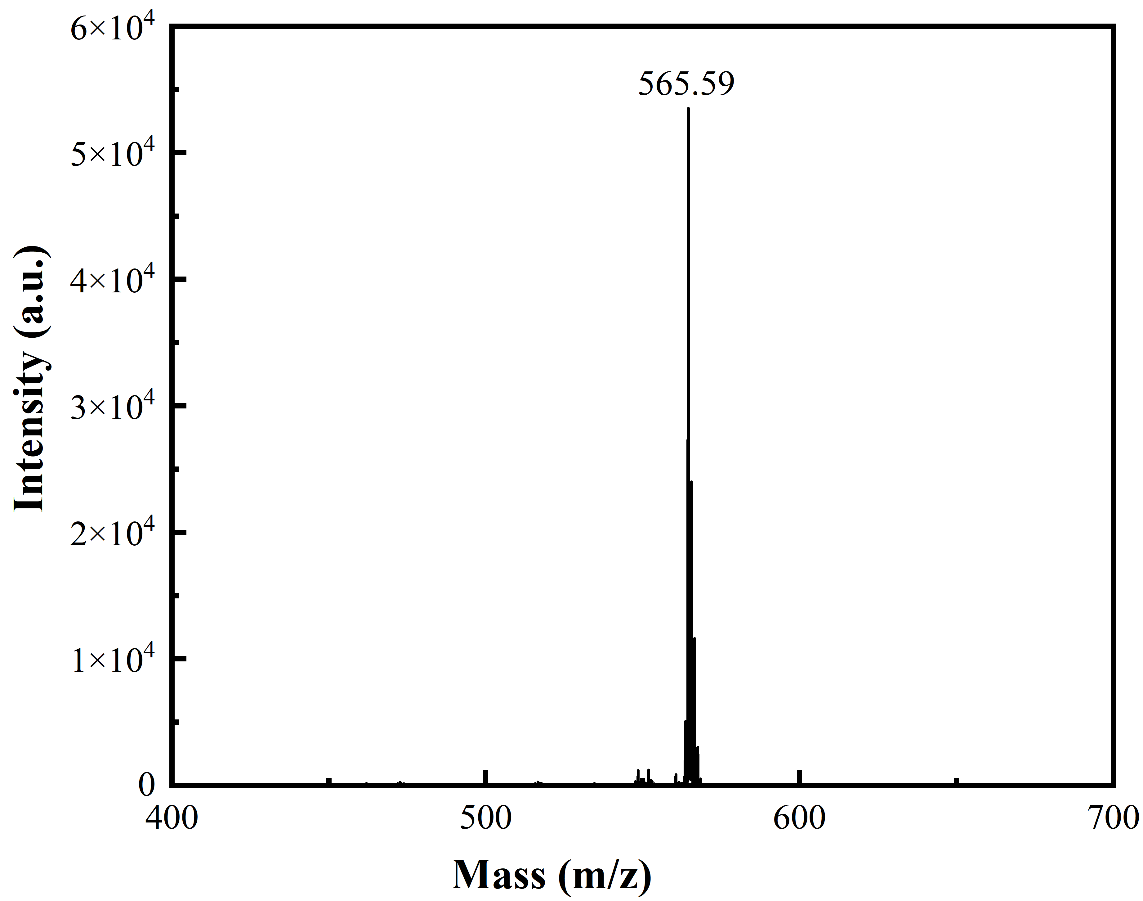


**Figure** **S12.** MALDI-TOF MS spectrum of **PT**.

**
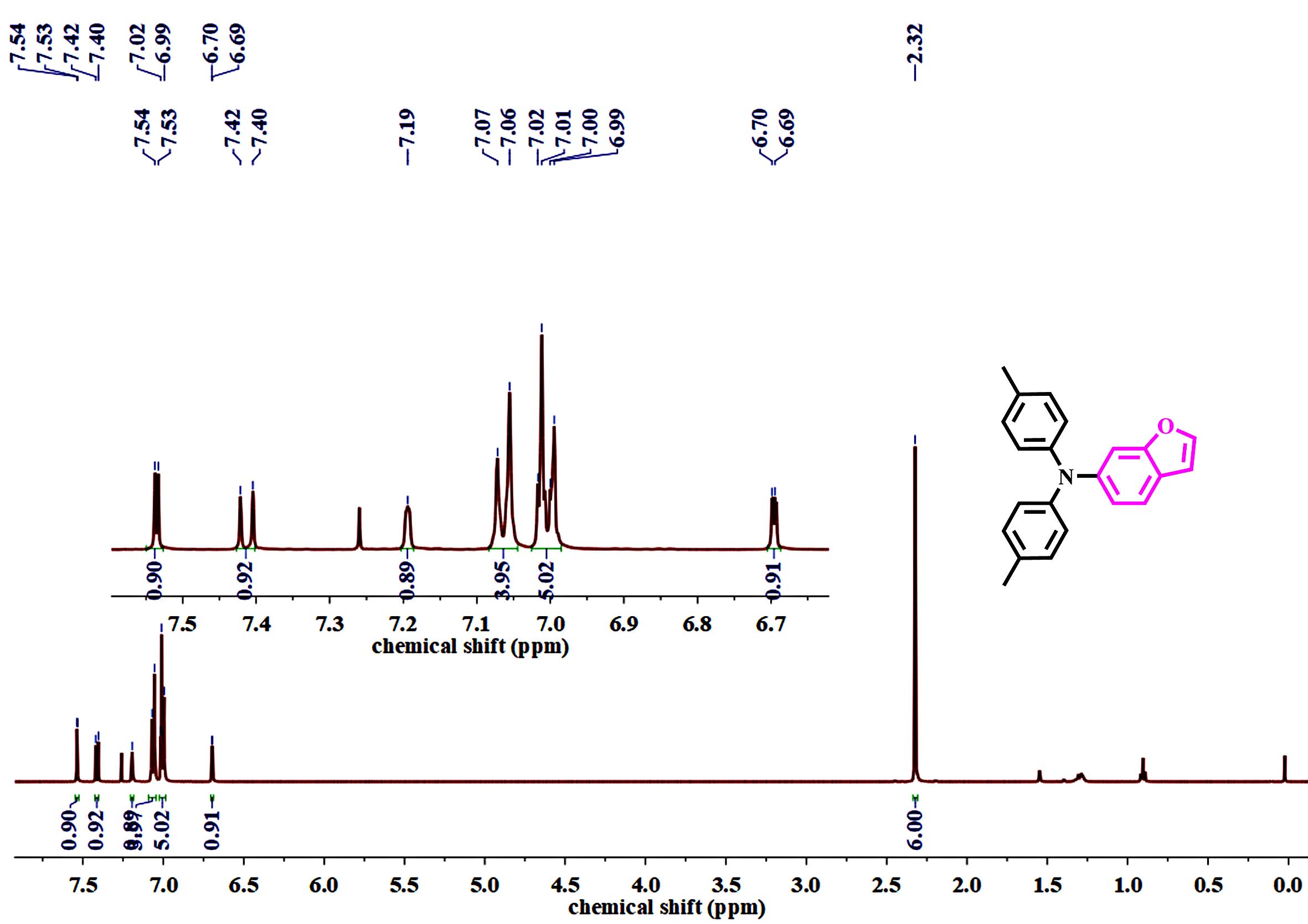
**

**Figure** **S13.** ^1^H NMR spectrum of **4a**.

**
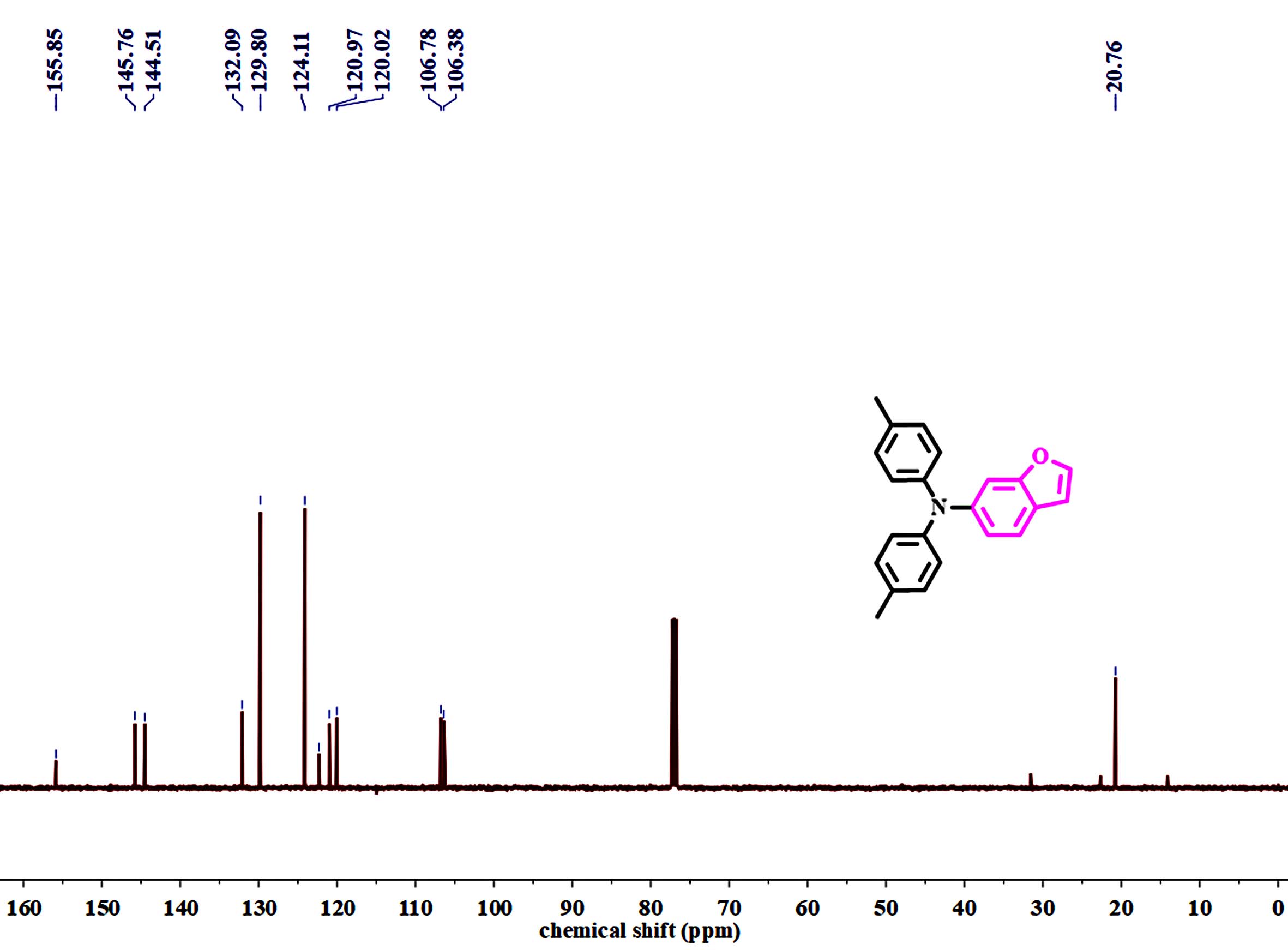
**

**Figure** **S14.** ^13^C NMR spectrum of **4a**.


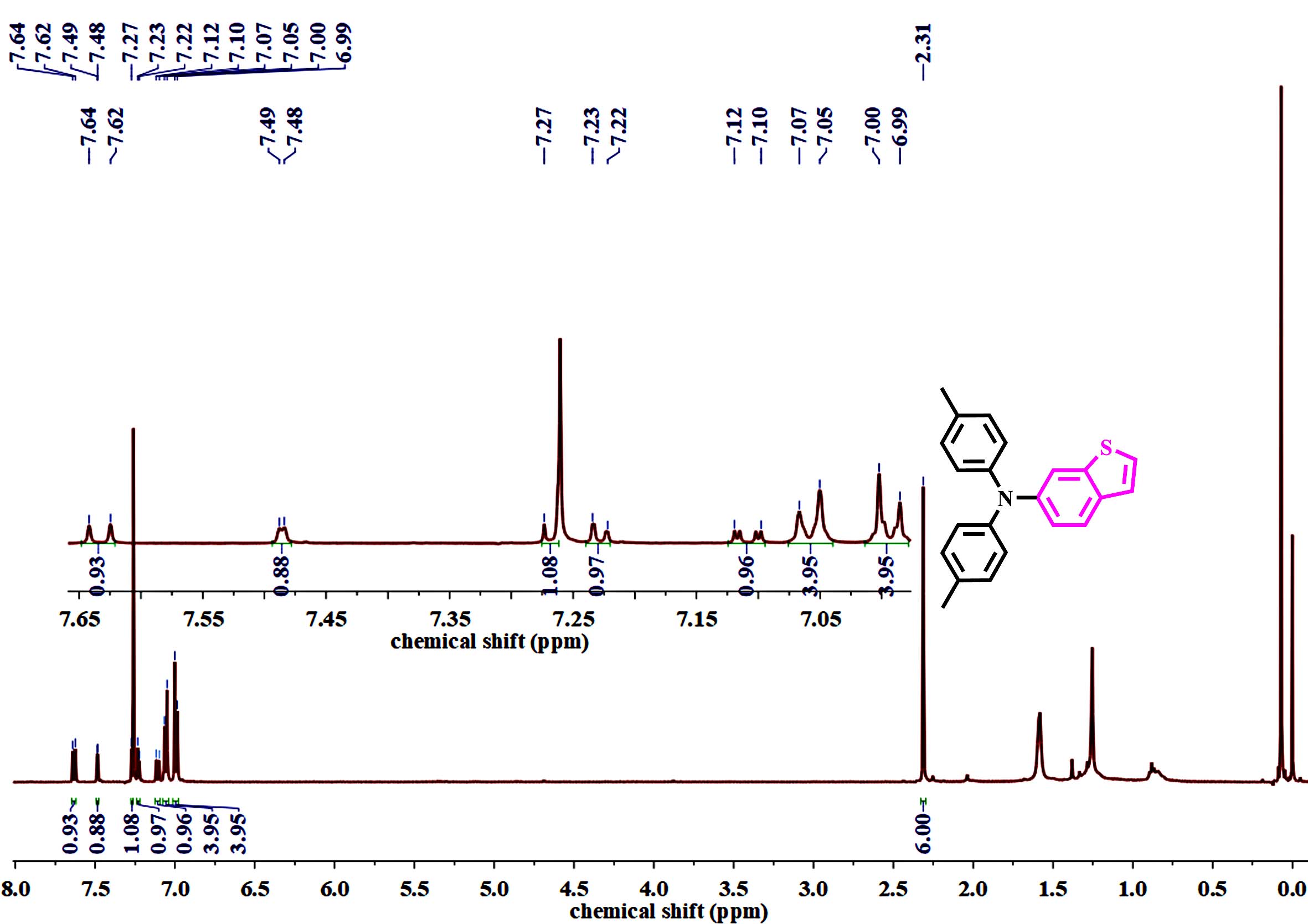


**Figure** **S15.** ^1^H NMR spectrum of **4b**.


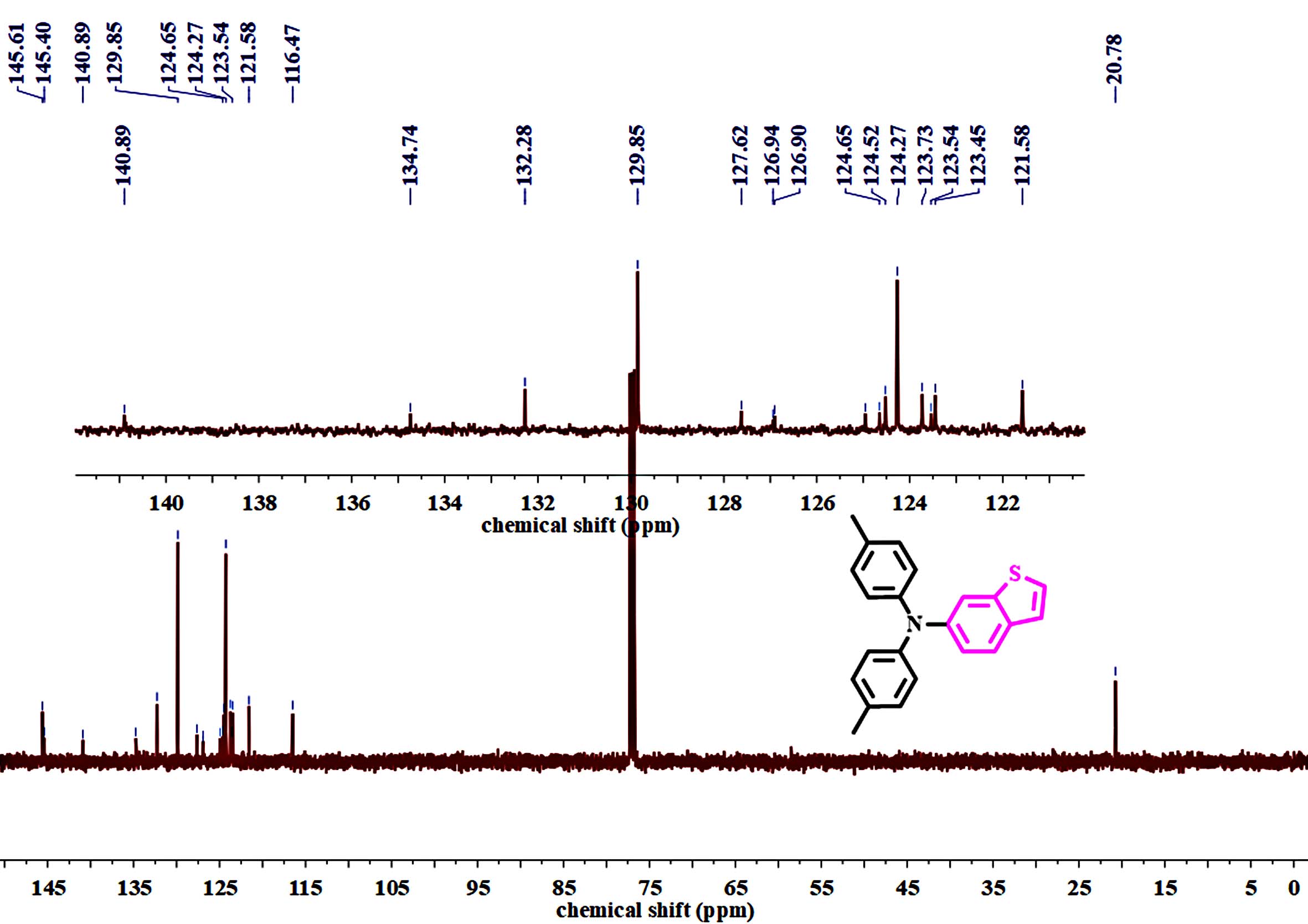


**Figure** **S16.** ^13^C NMR spectrum of **4b**.

**
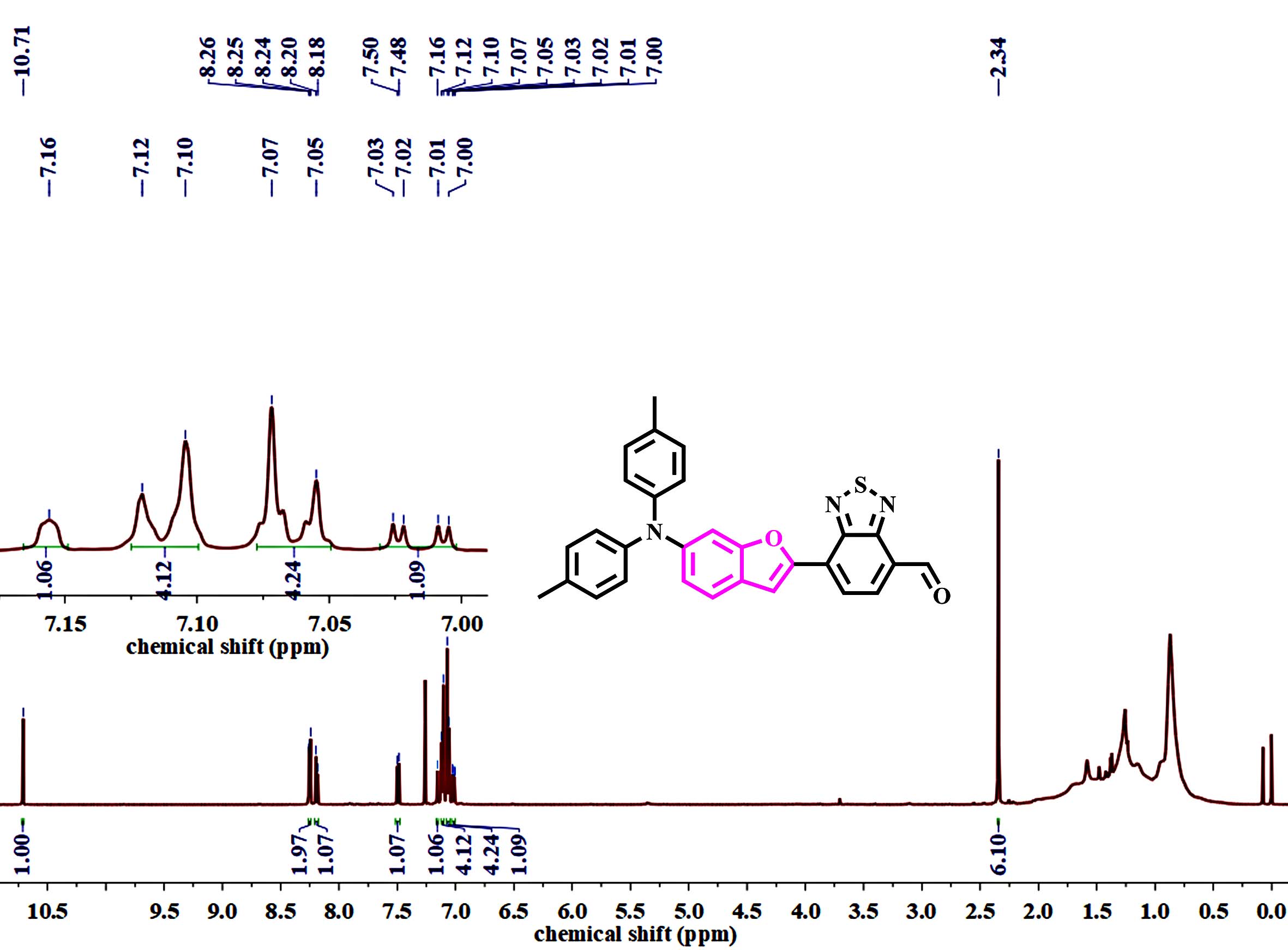
**

**Figure** **S17.** ^1^H NMR spectrum of **6a**.


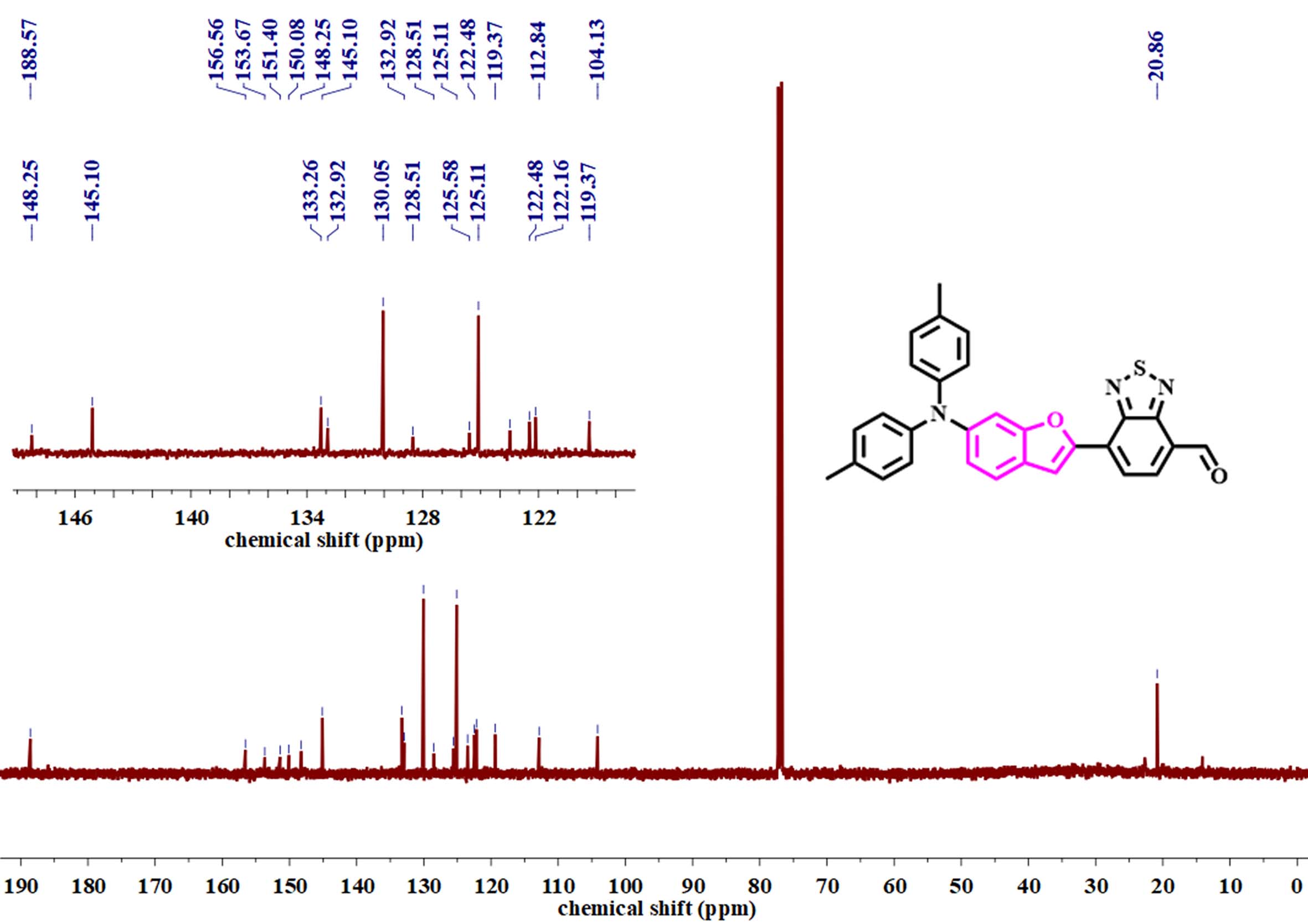


**Figure** **S18.** ^13^C NMR spectrum of **6a**.

**
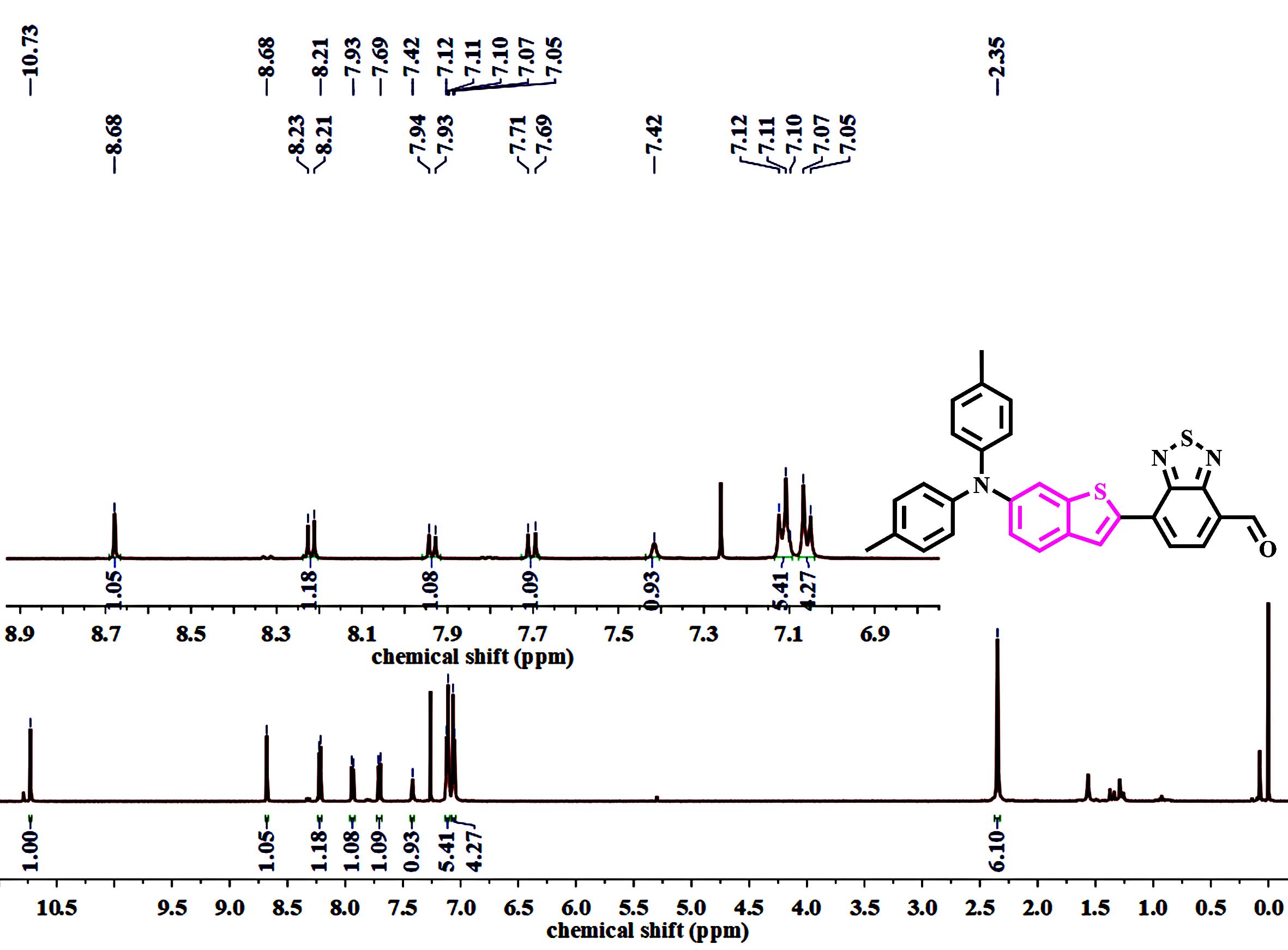
**

**Figure** **S19.** ^1^H NMR spectrum of **6b**.


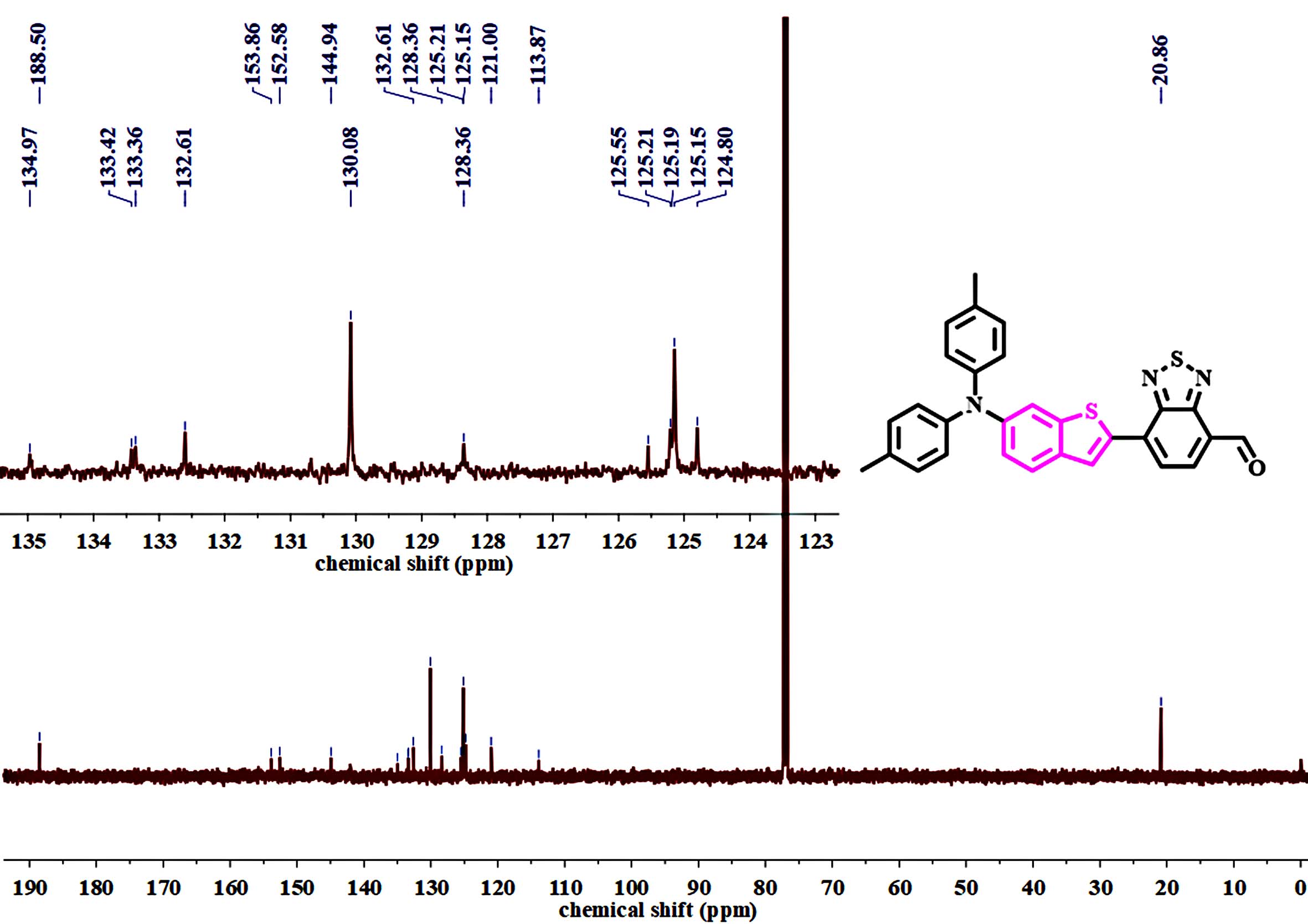


**Figure** **S20.** ^13^C NMR spectrum of **6b**.

**
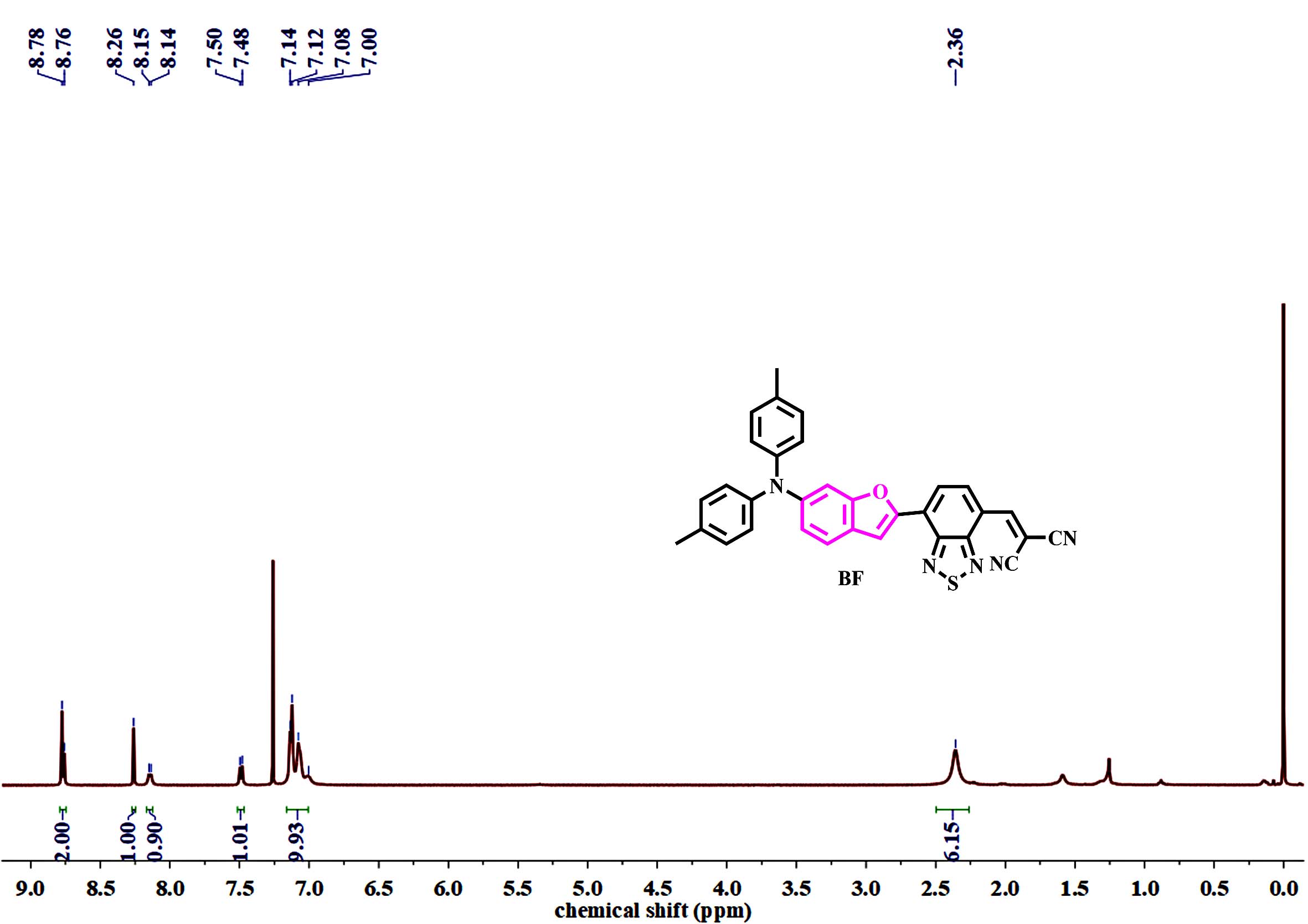
**

**Figure** **S21.** ^1^H NMR spectrum of **BF**.

**
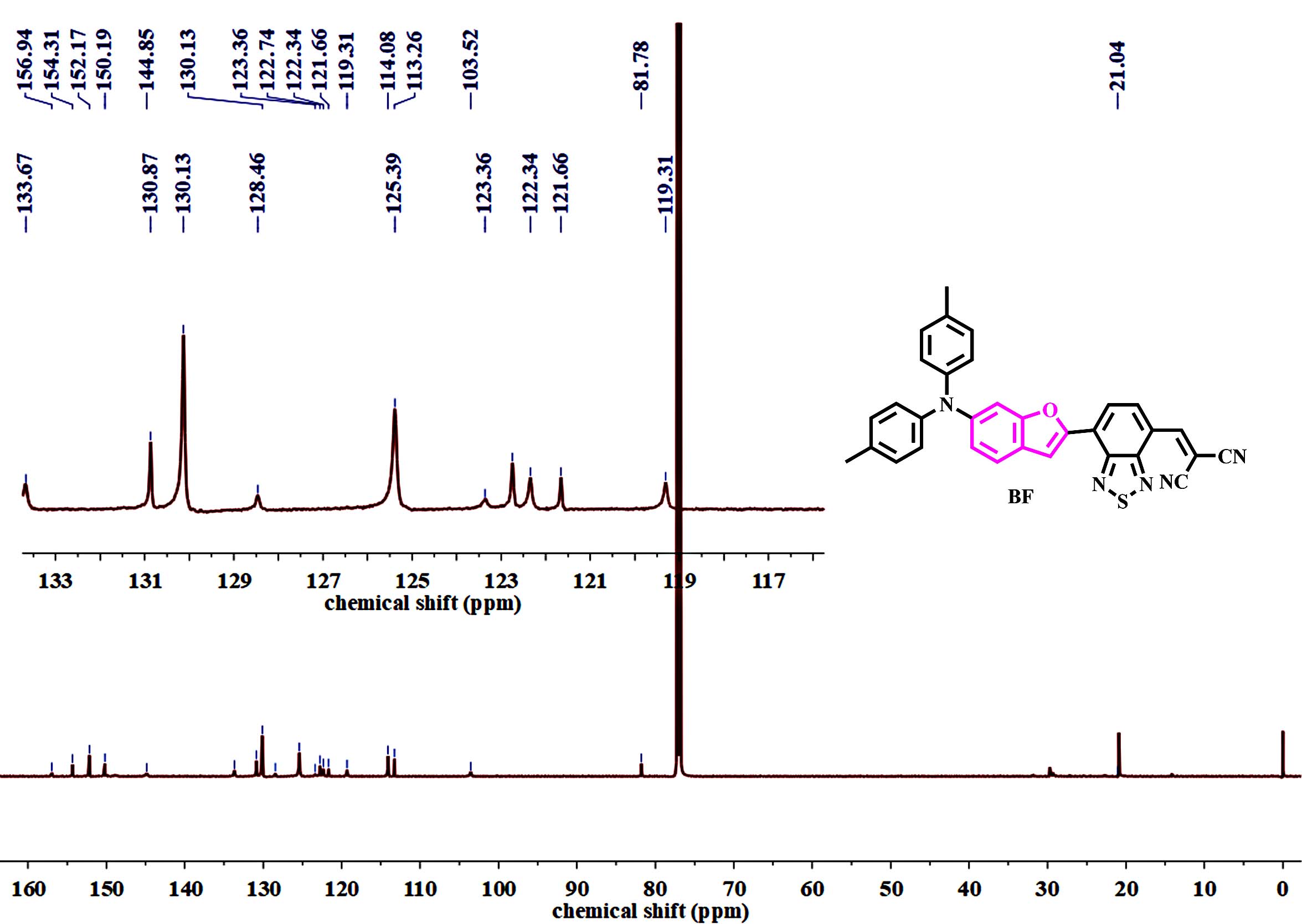
**

**Figure** **S22.** ^13^C NMR spectrum of **BF**.


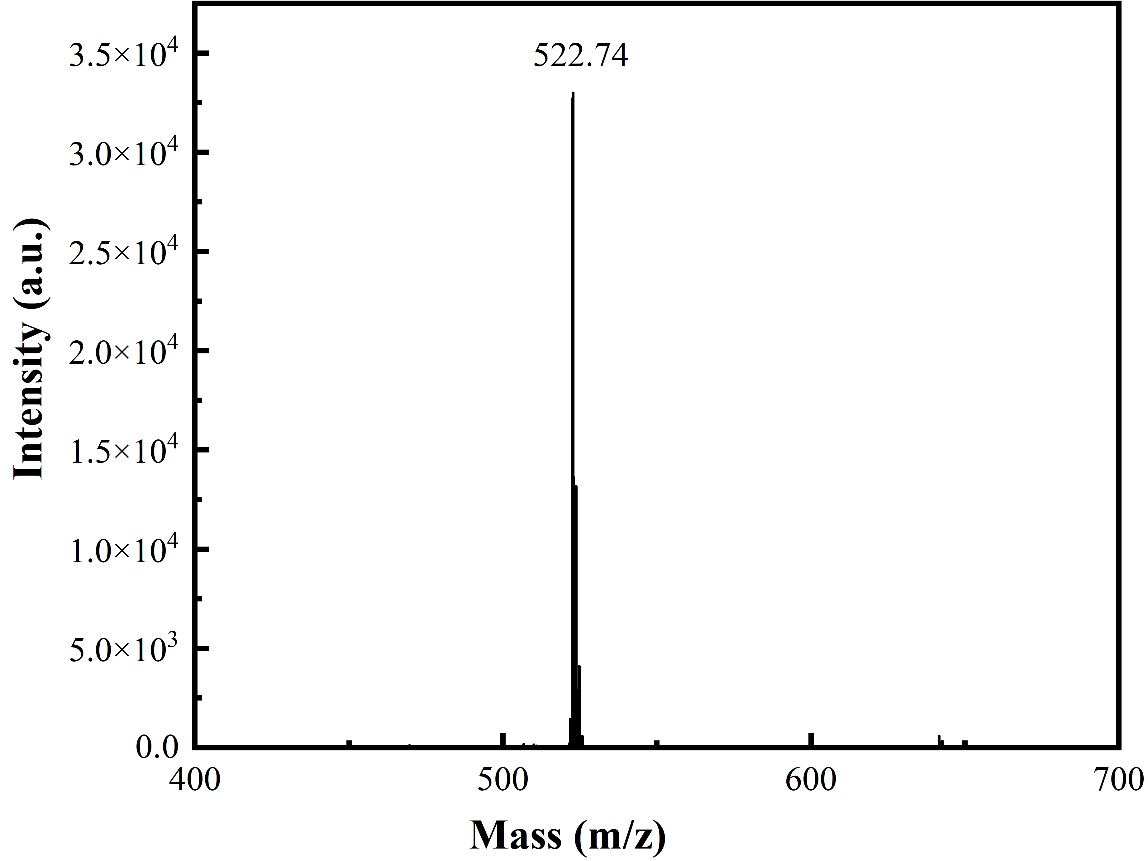


**Figure** **S23.** MALDI-TOF MS spectrum of **BF**.


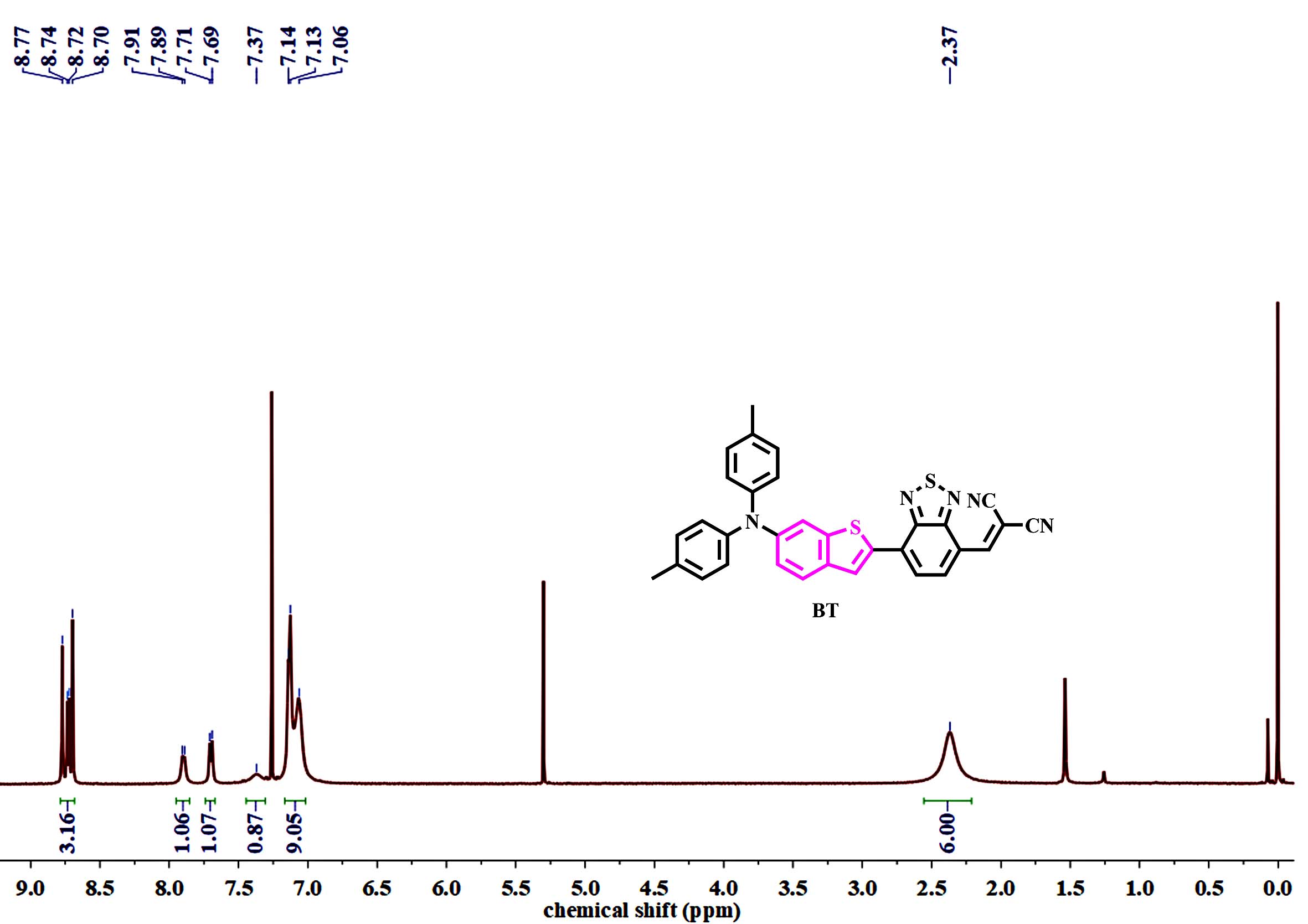


**Figure** **S24.** ^1^H NMR spectrum of **BT**.

**
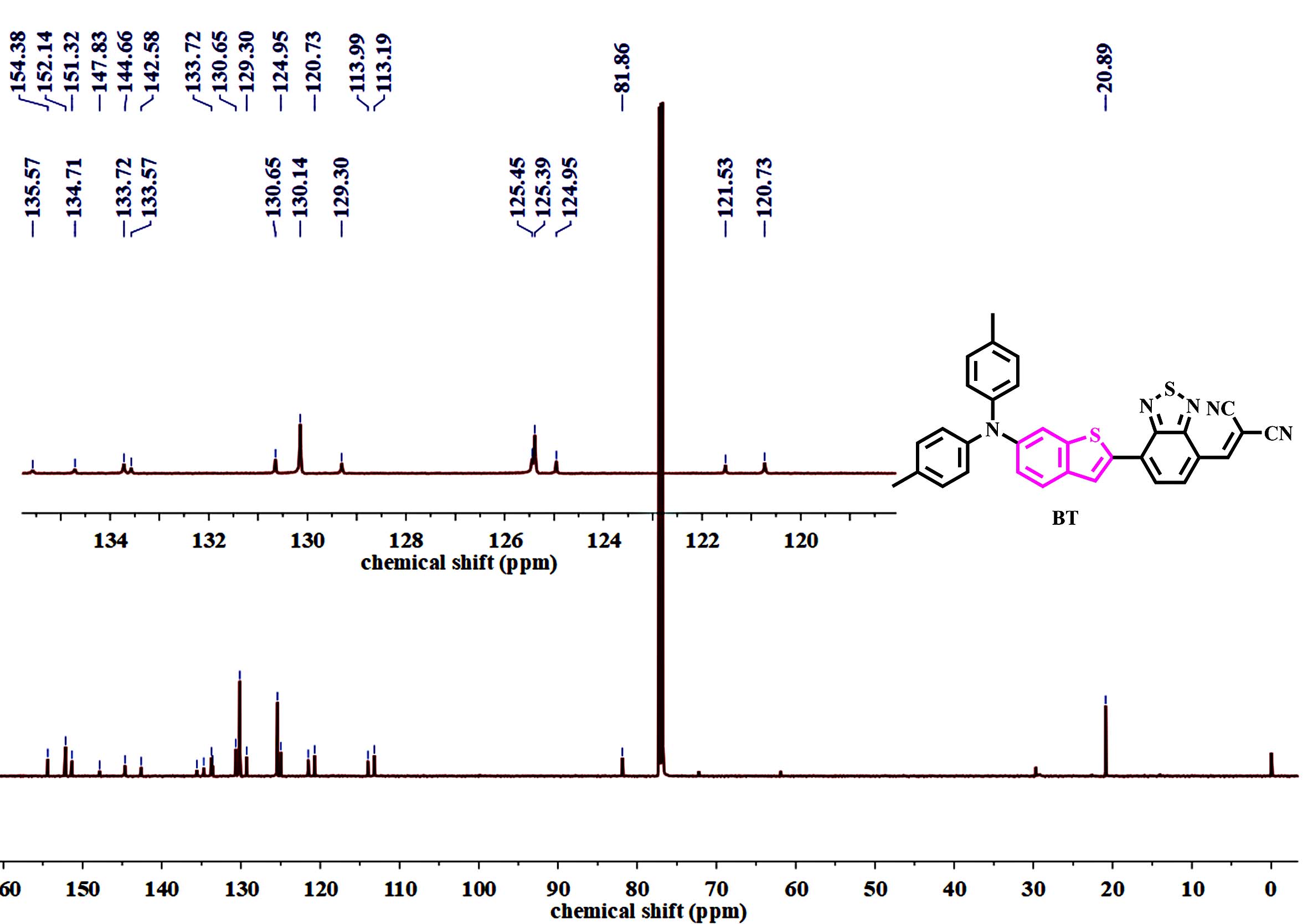
**

**Figure** **S25.** ^13^C NMR spectrum of **BT**.


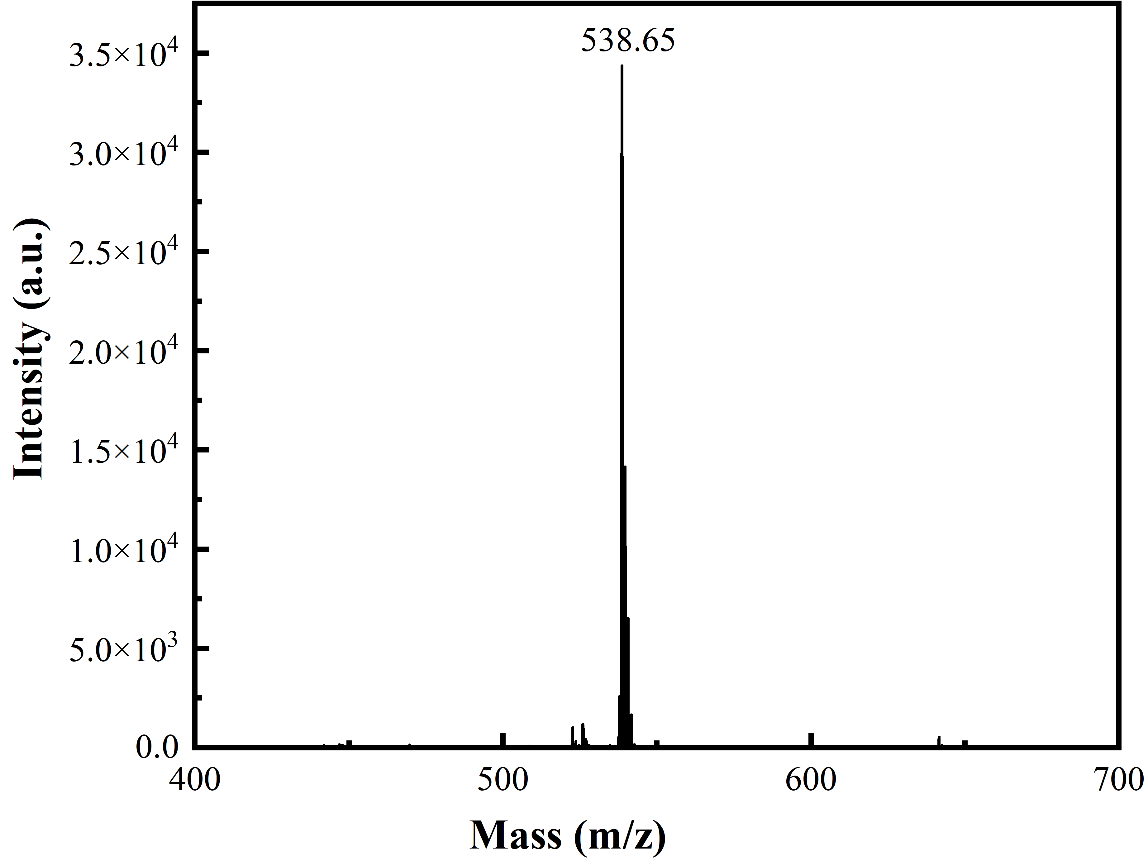


**Figure** **S26.** MALDI-TOF MS spectrum of **BT**.

**Theoretical calculations.**

The electronic and optical properties of PT, PF, BT, and BF were estimated by density functional theory (DFT) and time-dependent DFT (TD-DFT) calculations at the B3LYP/def2TZVP basis set in the Gaussian 16 program^[3]^. Quantitative molecular surface analysis^[4]^, electrostatic potential evaluation^[5]^ and orbital composition analysis^[6]^ were performed by using Multiwfn program (version 3.8)^[7]^ and VMD program (version 1.9.3)^[8]^. The molecular geometry data were extracted from the single crystals. As summarized in Tables S1-S4, the S_0_ to S_1_ transition of PT, PF, BT, and BF is composed of the HOMO to LUMO transition, which is referred to as the intramolecular charge transfer transition for the D-A-A-configured molecules. Apparently, the oscillator strength of the S_0_ to S_1_ transition for BT is stronger than that for PT, and the oscillator strength of the S_0_ to S_1_ transition for BF is stronger than that for PF as well. The larger oscillator strength of the S_0_ to S_1_ transition is attributed to the greater degree of HOMO-LUMO overlap of BT and BF due to the more coplanar molecular structure. The calculated result is consistent with the tendency of the extinction coefficient observed in the ultraviolet-visible absorption spectra.


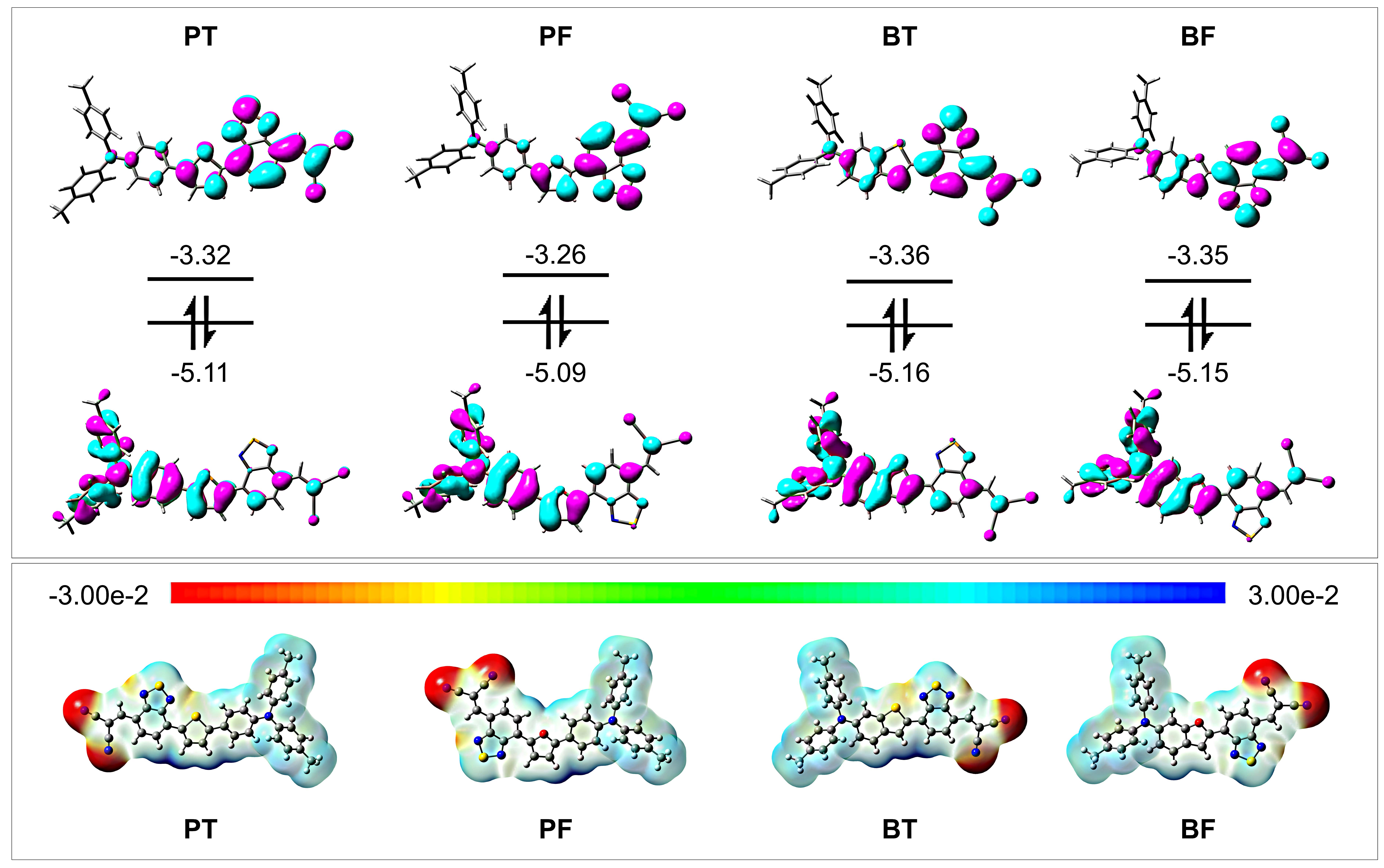


**Figure** **S27.** HOMO/LUMO density distributions and electrostatic potential of PT, PF, BT, and BF.


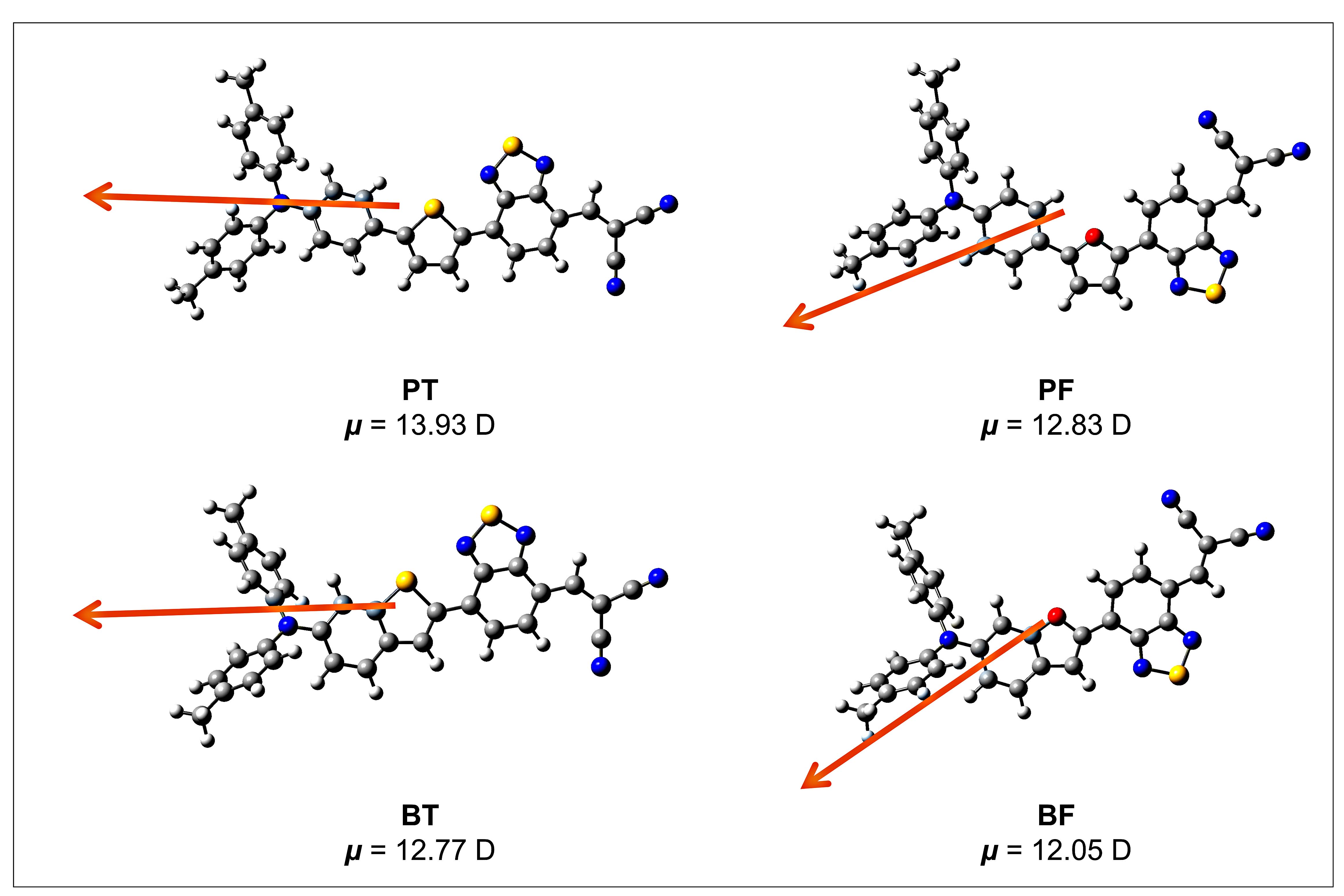


**Figure** **S28.** Dipole moment of PT, PF, BT, and BF.

**Table S1.** Electronic transition character of PT.

| Molecule | HOMO (eV) | | LUMO (eV) | GS dipole (Debye) | | | *f* | Composition of wave function |
| --- | --- | --- | --- | --- | --- | --- | --- | --- |
| PT | | -5.11 | -3.32 | | 13.93 | 0.6850 | | 0.71(H→L) |
|  |  |  |  |  |  | 0.3438 | | 0.68(H-1→L)+0.16(H→L+1) |
|  |  |  |  |  |  | 0.3882 | | -0.15(H-1→L)+0.68(H→L+1) |
|  |  |  |  |  |  | 0.1580 | | 0.10(H-4→L)+0.58(H-2→L)-0.37(H→L+2) |
|  |  |  |  |  |  | 0.2707 | | -0.16(H-3→L)+0.28(H-2→L)-0.33(H-1→L+1)-0.12(H→L+1)+0.50(H→L+2) |

**Table S2.** Electronic transition character of PF.

| Molecule | HOMO (eV) | LUMO (eV) | GS dipole (Debye) | | *f* | Composition of wave function |
| --- | --- | --- | --- | --- | --- | --- |
| PF | -5.09 | -3.26 | 12.83 | 0.6348 | | 0.71(H→L) |
|  |  |  |  | 0.2371 | | 0.67(H-1→L)+0.20(H→L+1) |
|  |  |  |  | 0.3666 | | -0.19(H-1→L)+0.66(H→L+1) |
|  |  |  |  | 0.4118 | | 0.11(H-3→L)-0.18(H-2→L)-0.42(H-1→L+1)-0.12(H→L+1)+0.50(H→L+2) |
|  |  |  |  | 0.2309 | | 0.25(H-5→L)+0.25(H-4→L)+0.20(H-2→L)+0.10(H-1→L)+0.46(H-1→L+1)+0.10(H-1→L+2)+0.28(H→L+2) |

**Table S3.** Electronic transition character of BT.

| Molecule | HOMO (eV) | LUMO (eV) | GS dipole (Debye) | *f* | Composition of wave function |
| --- | --- | --- | --- | --- | --- |
| BT | -5.16 | -3.36 | 12.77 | 0.7117 | 0.71(H→L) |
|  |  |  |  | 0.5082 | -0.19(H-2→L)-0.31(H-1→L)+0.59(H→L+1) |
|  |  |  |  | 0.1257 | -0.12(H-7→L)+0.16(H-6→L)-0.20(H-5→L)+0.57(H-1→L+1)+0.17(H→L+2)-0.20(H→L+3) |

**Table S4.** Electronic transition character of BF.

| Molecule | HOMO (eV) | LUMO (eV) | GS dipole (Debye) | ***f*** | Composition of wave function |
| --- | --- | --- | --- | --- | --- |
| BF | -5.15 | -3.35 | 12.05 | 0.6435 | 0.71(H→L) |
|  |  |  |  | 0.4937 | -0.39(H-1→L)+0.58(H→L+1) |
|  |  |  |  | 0.1717 | -0.16(H-6→L)+0.28(H-5→L)-0.33(H-1→L+1)+0.50(H→L+2) |
|  |  |  |  | 0.1877 | -0.14(H-7→L)-0.17(H-6→L)-0.21(H-5→L)+0.52(H-1→L+1)+0.26(H→L+2)-0.17(H→L+3) |


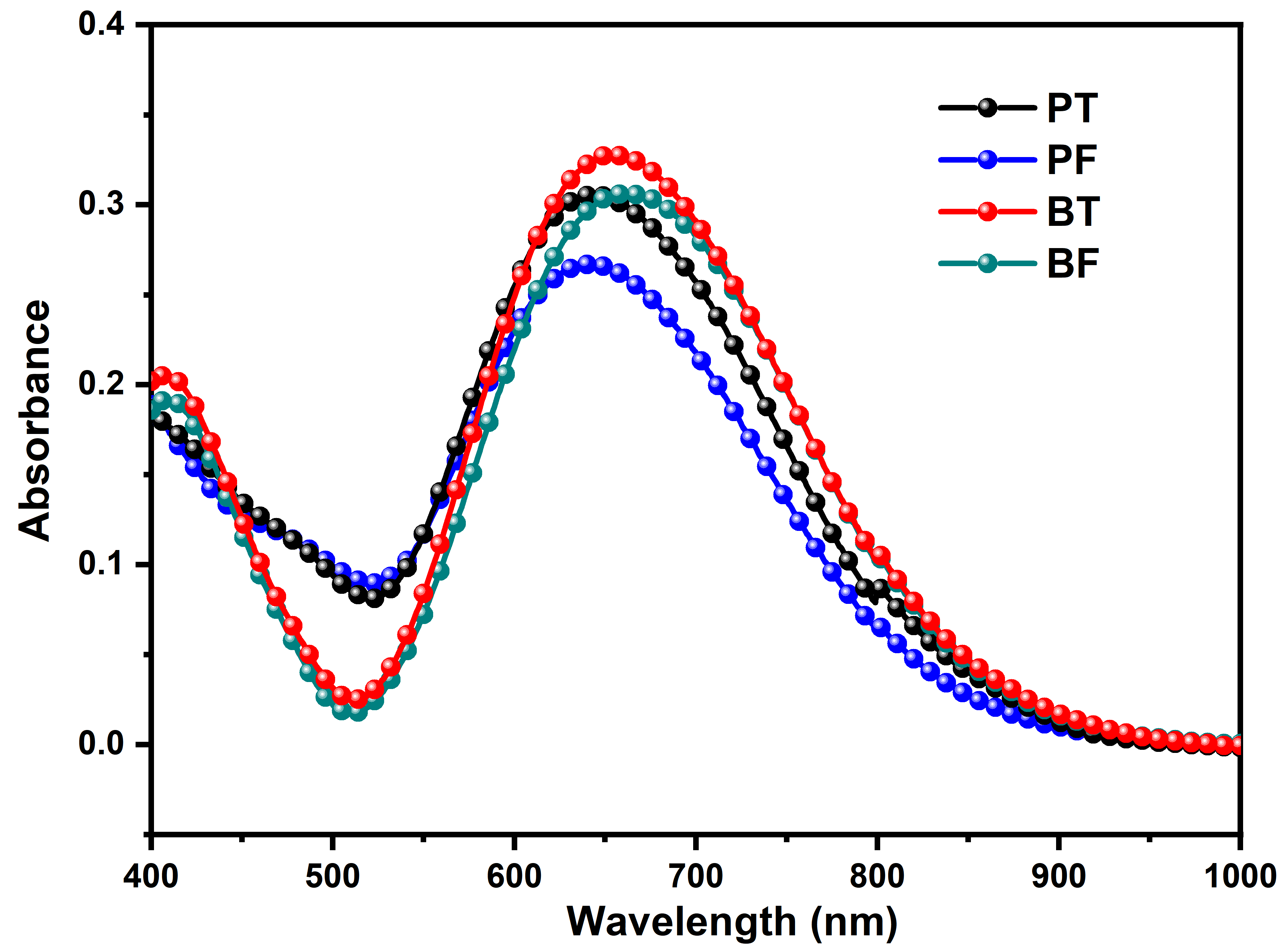


**Figure** **S29.** UV-Vis absorption spectra of PT, PF, BT, and BF in thin films.


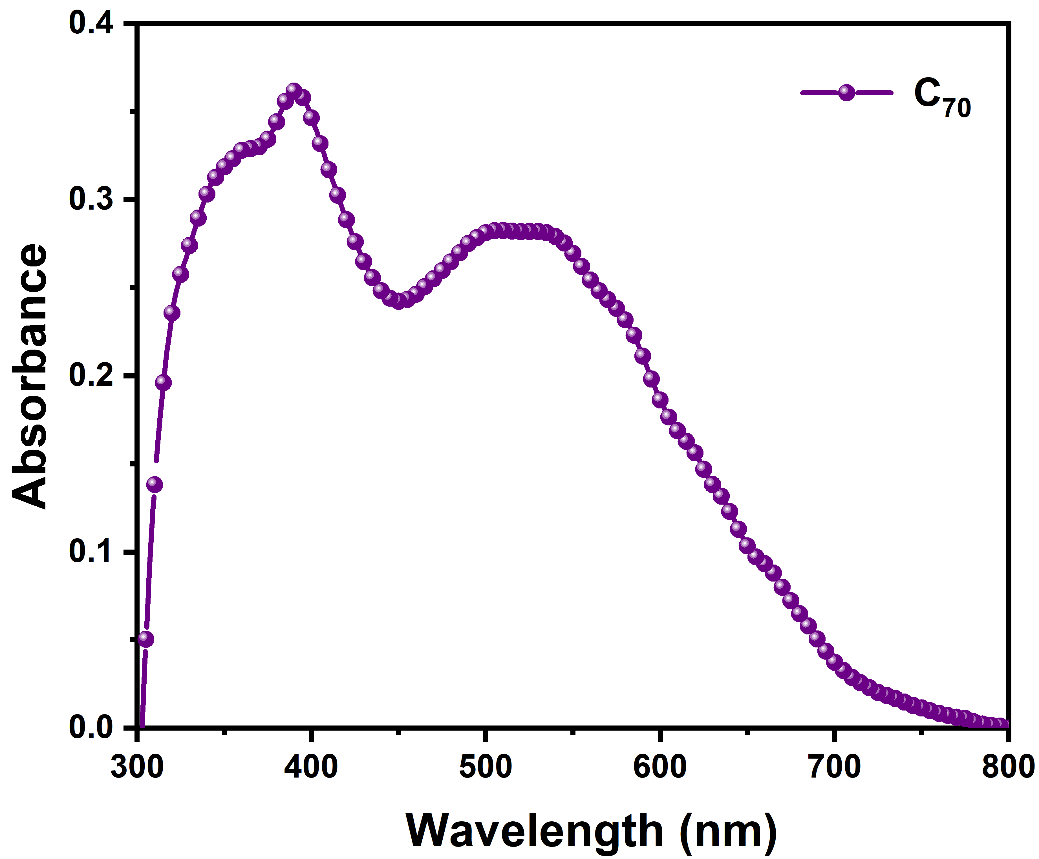


**Figure** **S30.** UV-Vis absorption spectra of C_70_ in thin films.


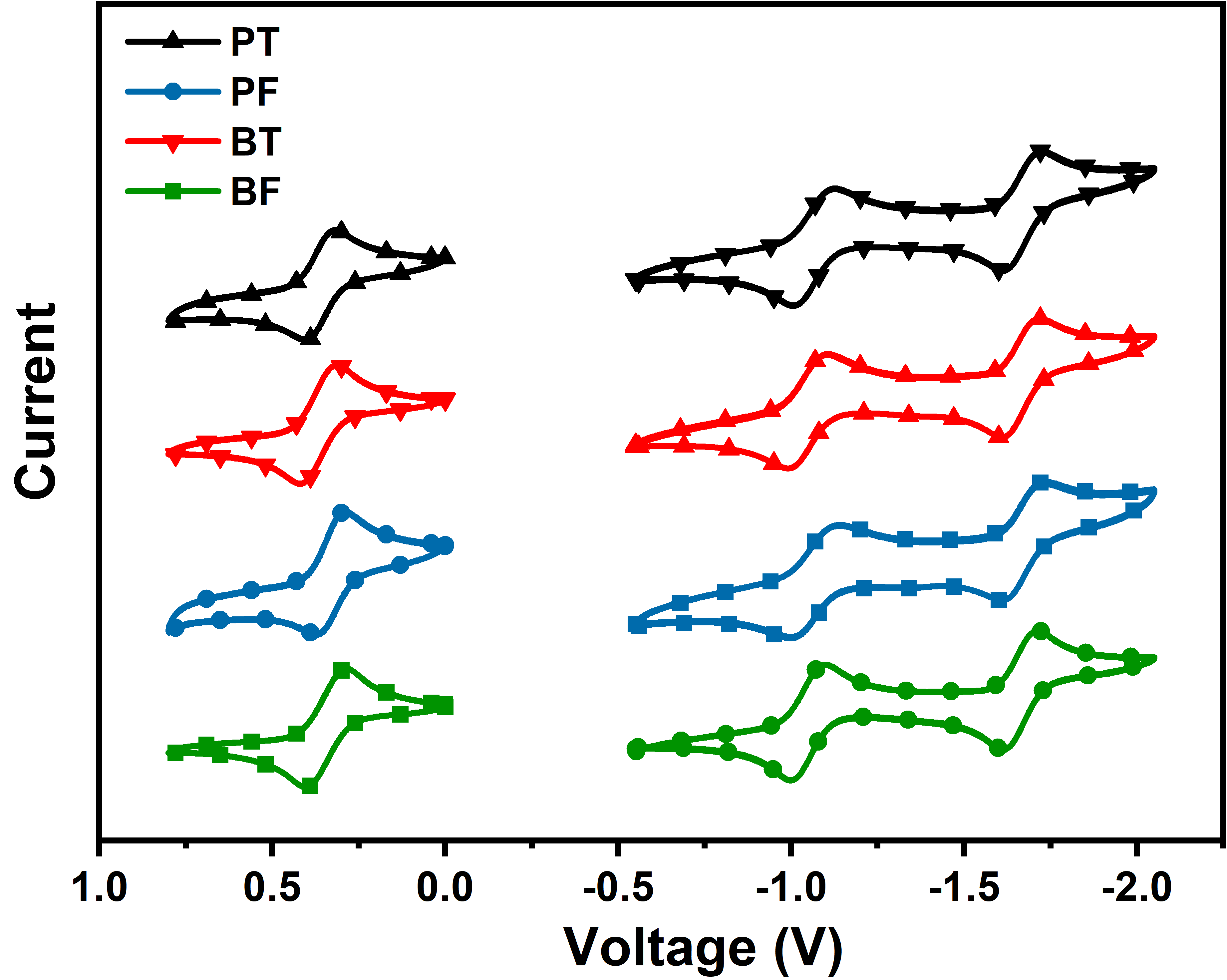


**Figure** **S31.** Cyclic voltammograms of PT, PF, BT, and BF.


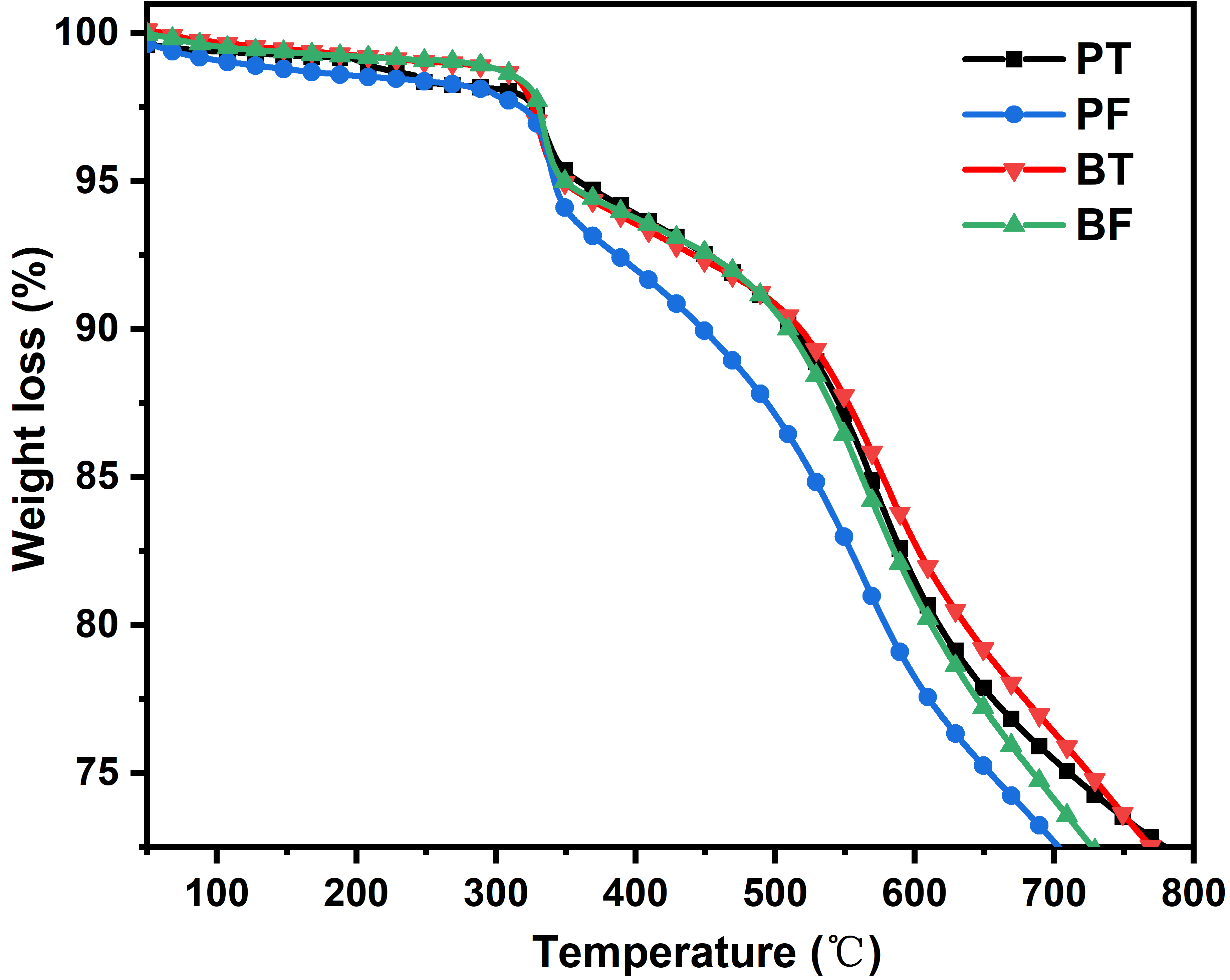


**Figure** **S32.** TGA curves of PT, PF, BT, and BF.

**X-ray single crystal structure characterizations for PT, PF, BT and BF.**

Single crystals of the newly synthesized donor-acceptor-acceptor (D-A-A)-configured molecules, PT, PF, BT, and BF suitable for single X-ray diffraction analyses were obtained via slow diffusion method with orthogonal solvents (chloroform as dissolving solvent and methanol as orthogonal solvent). Crystallographic data were acquired on a Rigaku Oxford SuperNova Diffractometer equipped with Cu X-ray source (λ = 1.54184 Å) radiation at 100 K. The initial structures were solved using the SHELX-XT structure solution program with the direct method, followed by refinement with the XL refinement package using Least Squares minimization, as implemented in OLEX2^[9-10]^. Hydrogen atoms were placed in calculated positions and refined using the riding model. Molecular stacking, π-π interactions, and inter/intramolecular distances were determined using Mercury 3.10.2. The supplementary crystallographic data for PT, PF, BT, and BF are deposited in the Cambridge Crystallographic Data Centre (CCDC) under accession numbers 2480849, 2480850, 2480851 and 2480854, respectively, and are available free of charge from www.ccdc.cam.ac.uk/data_request/cif. The refined CIF files were validated using the online service at ‘http://checkcif.iucr.org/’. Crystallographic data and experimental parameters are provided in Table S5.

**Table S5.** Crystal data for PT, PF, BT, and BF.

|  | | **PT** | | | **PF** | **BT** | | **BF** |
| --- | --- | --- | --- | --- | --- | --- | --- | --- |
| Formula | C_34_H_23_N_5_S_2_ | | | C_70_H_30_N_10_O_2_S_2_ | | | C_64_H_42_N_10_S_4_ | C_33_H_13_N_5_OS |
| Crystal system | | triclinic | | triclinic | | | triclinic | triclinic |
| T, K | | 100 | | 100 | | | 100 | 100 |
| Space group | | *P-1* | | *P-1* | | | *P-1* | *P-1* |
| a, Å | | 7.1964(3) | | 6.83140(10) | | | 9.8288(4) | 7.2150(2) |
| b, Å | | | 12.0515(5) | 18.6714(4) | | | 12.7635(5) | 12.0800(2) |
| c, Å | | | 19.0072(8) | 24.6898(5) | | | 25.2874(9) | 17.2811(3) |
| α, deg | | | 103.587(4) | 89.508(2) | | | 77.889(3) | 86.183(2) |
| β, deg | | | 100.114(5) | 83.840(2) | | | 77.889(3) | 82.677(2) |
| γ, deg | | | 102.134(4) | 79.799(2) | | | 73.791(4) | 86.202(2) |
| Volume, Å^3^ | | | 1521.98(12) | 3081.38(10) | | | 2978.3(2) | 1488.03(6) |
| Z | | | 2 | 2 | | | 2 | 2 |
| Density, gcm^-3^ | | | 1.2344 | 1.4421 | | | 1.2035 | 1.4350 |
| *F* (000) | | | 590.9 | 1385.1 | | | 1125.6 | 665.5 |
| No. of reflns collected | | | 16064 | 39427 | | | 32326 | 16246 |
| No. of indep reflins (*R*_int_) | | | 5668(0.0515) | 11655(0.0362) | | | 11241(0.0809) | 5723(0.0301) |
| R1, wR_2_ ((I>2σ(I) | | | 0.04, 0.09 | 0.03(0.10) | | | 0.06, 0.16 | 0.05, 0.13 |
| R1, wR_2_ (all data) | | | 0.05, 0.10 | 0.04, 0.10 | | | 0.08, 0.17 | 0.05, 0.14 |


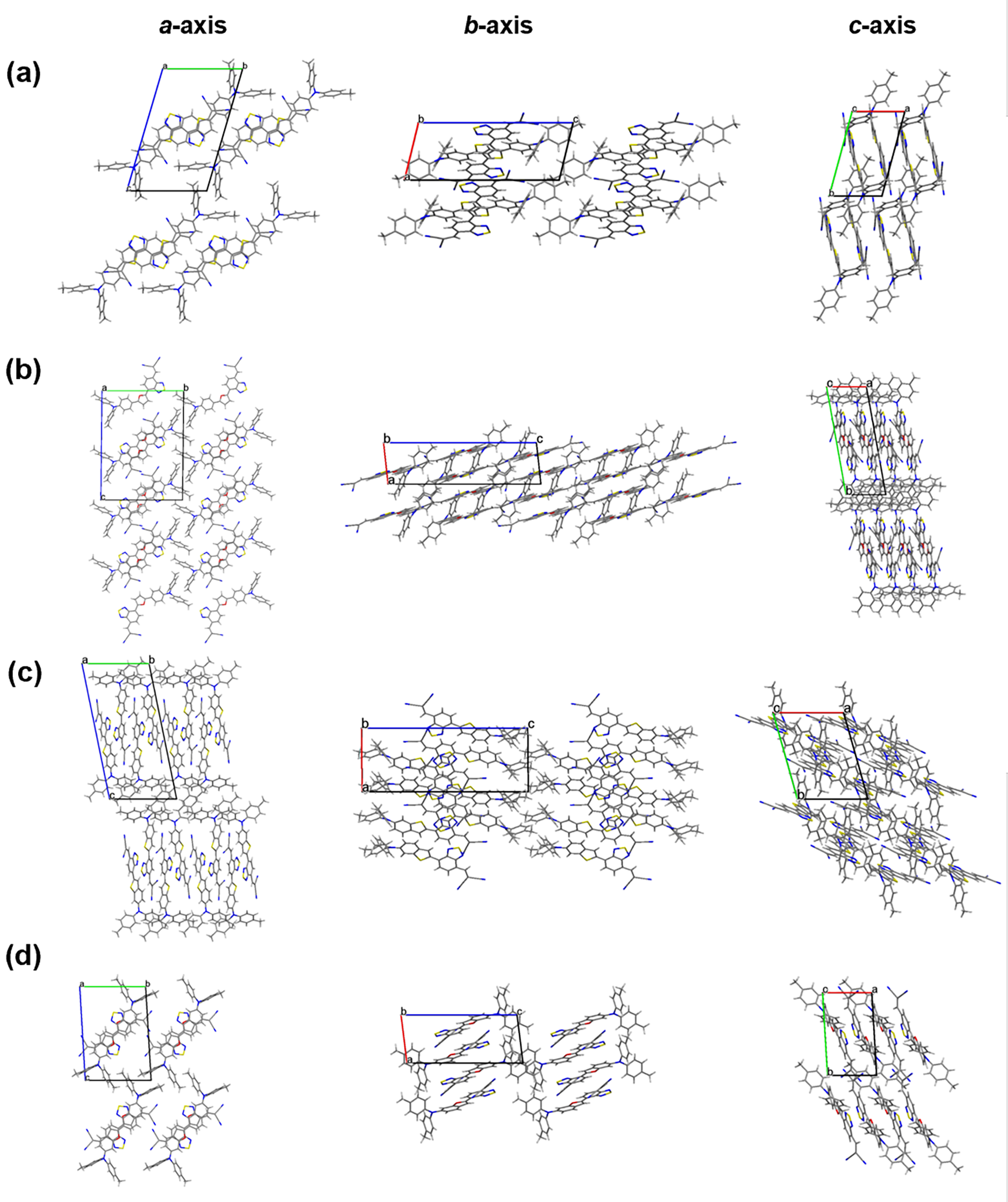


**Figure** **S33.** The crystal stacking structure of PT (a), PF (b), BT (c), and BF (d) viewed along *a*-axis, *b*-axis and *c*-axis.

**Table S6.** Photovoltaic parameters of v-OSCs based on PT: C_70_ with different D:A ratios of the active layer.

| D:A ratio [w/w] | *V*_oc_ [V] | *J*_sc_ [mA cm^-2^] | FF [%] | PCE [%] |
| --- | --- | --- | --- | --- |
| 1: 1 | 0.83±0.001 | 14.02±0.65 | 60.24±0.75 | 7.02±0.33 |
| 1: 2 | 0.83±0.01 | 15.52±0.14 | 65.80±0.25 | 8.46±0.08 |
| 1: 3 | 0.84±0.002 | 15.20±0.36 | 61.07±1.22 | 7.79±0.30 |

The average parameters and standard deviations were obtained from 10 independent cells.

**Table S7.** Photovoltaic parameters of v-OSCs based on BT: C_70_ with different D:A ratios of the active layer.

| D:A ratio [w/w] | *V*_oc_ [V] | *J*_sc_ [mA cm^-2^] | FF [%] | PCE [%] |
| --- | --- | --- | --- | --- |
| 1: 1 | 0.84±0.01 | 16.67±0.17 | 65.65±0.25 | 9.23±0.12 |
| 1: 2 | 0.86±0.01 | 17.83±0.09 | 68.28 ±0.30 | 10.45±0.06 |
| 1: 3 | 0.85±0.01 | 16.83±0.37 | 66.27±0.43 | 9.45±0.26 |

The average parameters and standard deviations were obtained from 10 independent cells.

**Table S8.** Photovoltaic parameters of v-OSCs based on PT: C_70_ with different thicknesses of the active layer.

| Thickness [nm] | *V*_oc_ [V] | *J*_sc_ [mA cm^-2^] | FF [%] | PCE [%] |
| --- | --- | --- | --- | --- |
| 60 | 0.83±0.001 | 15.25±0.35 | 65.12±0.89 | 8.26±0.22 |
| 80 | 0.83±0.01 | 15.52±0.14 | 65.80±0.25 | 8.46±0.08 |
| 100 | 0.84±0.004 | 15.39±0.22 | 64.57±0.76 | 8.37±0.16 |

The average parameters and standard deviations were obtained from 10 independent cells.

**Table S9.** Photovoltaic parameters of v-OSCs based on BT: C_70_ with different thicknesses of the active layer.

| Thickness [nm] | *V*_oc_ [V] | *J*_sc_ [mA cm^-2^] | FF [%] | PCE [%] |
| --- | --- | --- | --- | --- |
| 60 | 0.86±0.01 | 17.56±0.39 | 68.33±0.34 | 10.29±0.21 |
| 80 | 0.86±0.01 | 17.83±0.09 | 68.28 ±0.30 | 10.45±0.06 |
| 100 | 0.86±0.04 | 16.37±0.40 | 64.93±0.42 | 9.10±0.18 |

The average parameters and standard deviations were obtained from 10 independent cells.


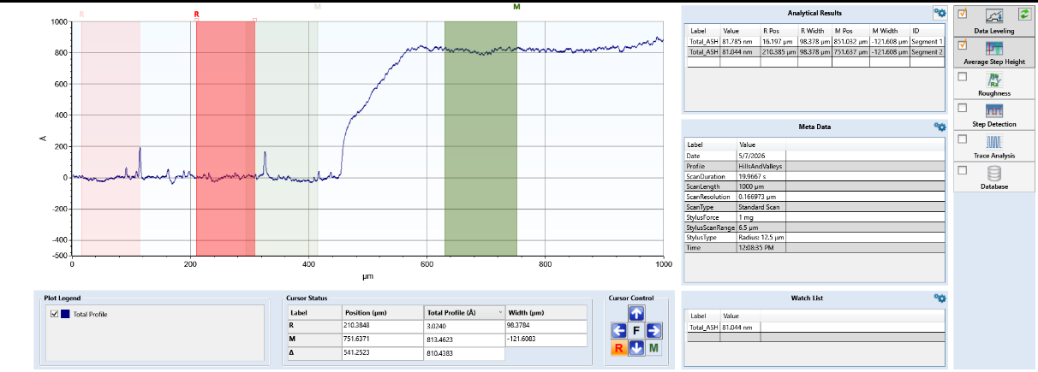


**Figure** **S34.** Stylus profiler image of the active layer.


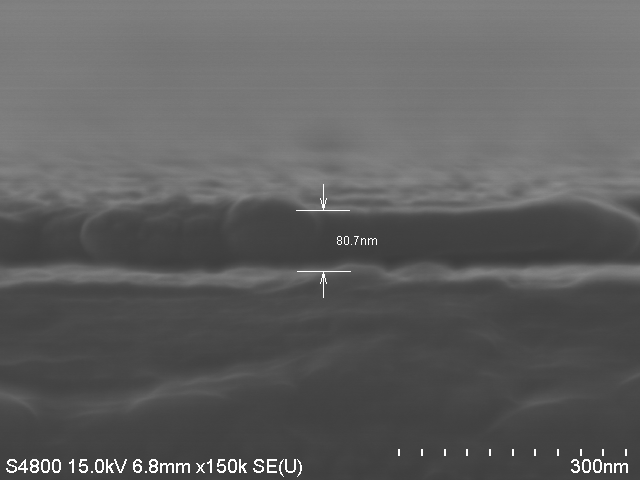


**Figure** **S35.** Cross-sectional SEM image of the active layer.


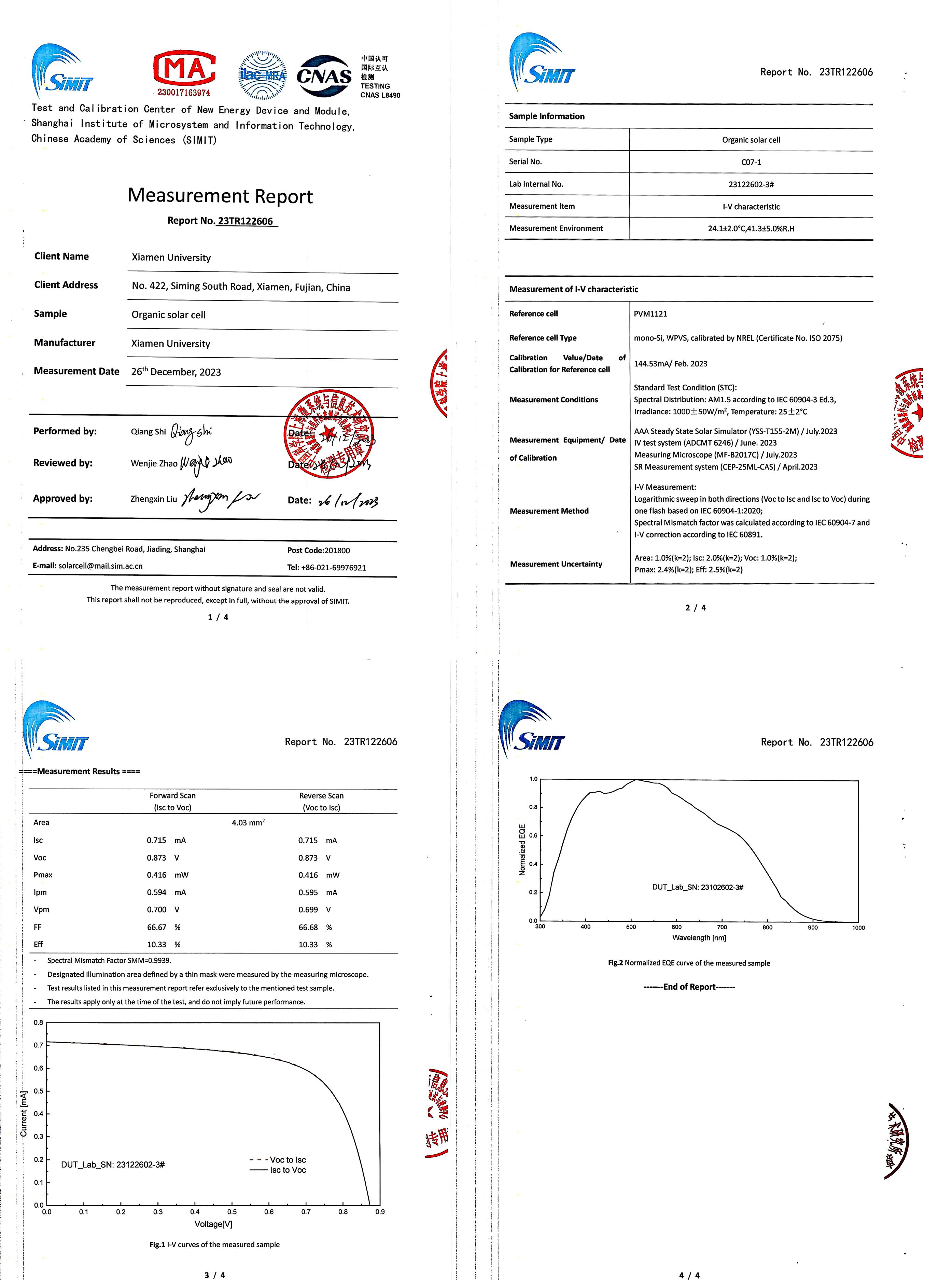


**Figure** **S36.** Certification report for BT: C_70_ device by Shanghai Institute of Microsystem and Information Technology, Chinese Academy of Sciences (SIMIT) in Shanghai, China.

**Table S10.** Summary of photovoltaic parameters of v-OSCs based on small molecule donors reported in the literature.

| Molecules | | *V*_oc_ (V) | *J*_sc_ (mA cm^-2^) | | FF (%) | PCE (%) | | Refs. |
| --- | --- | --- | --- | --- | --- | --- | --- | --- |
| TPTPA | 0.90 | | 3.60 | 0.66 | | 2.20 | ^[11]^ | |
| N-SpiroTPA | 0.94 | | 10.84 | 0.50 | | 5.09 | ^[12]^ | |
| DCPNT | 0.91 | | 12.59 | 0.58 | | 6.70 | ^[13]^ | |
| DPTMM | 0.99 | | 6.29 | 0.64 | | 4.00 | ^[14]^ | |
| DTTz | 0.95 | | 12.01 | 0.54 | | 6.37 | ^[15]^ | |
| DTTh | 0.87 | | 11.04 | 0.57 | | 5.41 | ^[15]^ | |
| HB194 | 0.96 | | 13.00 | 0.49 | | 6.10 | ^[16]^ | |
| Se-DHIn-DCN | 0.93 | | 11.70 | 0.57 | | 6.20 | ^[17]^ | |
| P4-Ph4-DIP | 0.99 | | 2.90 | 0.76 | | 1.92 | ^[18]^ | |
| DCV3T | 0.94 | | 4.06 | 0.56 | | 1.69 | ^[19]^ | |
| DCV5T-1,5Me | 0.91 | | 9.60 | 0.63 | | 4.80 | ^[20]^ | |
| DCV5T-2,3Me | 0.95 | | 9.40 | 0.62 | | 4.80 | ^[20]^ | |
| DCV5T-3Me | 0.95 | | 11.50 | 0.63 | | 6.90 | ^[20]^ | |
| DTPT | 0.98 | | 10.90 | 0.52 | | 5.64 | ^[21]^ | |
| DTPT-Et | 0.89 | | 10.00 | 0.67 | | 5.96 | ^[22]^ | |
| DTPTT-Pent | 0.96 | | 12.20 | 0.61 | | 7.10 | ^[23]^ | |
| CBC | 0.93 | | 10.05 | 0.43 | | 4.22 | ^[24]^ | |
| CBCIF | 0.95 | | 9.97 | 0.50 | | 4.94 | ^[24]^ | |
| DTA-FLDCN | 0.99 | | 7.64 | 0.53 | | 4.04 | ^[25]^ | |
| DIBSQ | 0.87 | | 13.69 | 0.53 | | 6.32 | ^[26]^ | |
| DTP-t-Cz | 1.06 | | 7.78 | 0.37 | | 3.03 | ^[27]^ | |
| DTP-BPOZ | 0.84 | | 10.39 | 0.48 | | 4.23 | ^[27]^ | |
| BDP-H | 0.83 | | 10.90 | 0.60 | | 5.50 | ^[28]^ | |
| BDP-J | 0.90 | | 8.50 | 0.55 | | 4.20 | ^[28]^ | |
| BDP-CF_3_-H | 0.89 | | 6.10 | 0.45 | | 2.50 | ^[29]^ | |
| BDP- CF_3_-Me | 0.85 | | 9.90 | 0.54 | | 4.60 | ^[29]^ | |
| BDP-C_2_F_5_-OMe | 0.76 | | 13.60 | 0.62 | | 6.40 | ^[30]^ | |
| ZnPc | 0.67 | | 5.28 | 0.62 | | 2.23 | ^[31]^ | |
| CuPc | 0.54 | | 15.60 | 0.61 | | 5.30 | ^[32]^ | |
| *a*-6T | 0.94 | | 12.04 | 0.54 | | 6.02 | ^[33]^ | |
| BSubNC | 1.04 | | 10.10 | 0.67 | | 6.86 | ^[31]^ | |
| DTCTB | 0.90 | | 11.15 | 0.65 | | 6.55 | ^[34]^ | |
| DTCTBO | 0.95 | | 10.52 | 0.60 | | 5.96 | ^[34]^ | |
| DTDCPB | 0.93 | | 15.80 | 0.67 | | 9.60 | ^[35]^ | |
| antiBu-BTDC | 0.93 | | 14.40 | 0.56 | | 7.50 | ^[36]^ | |
| nBu-BTDC | 0.92 | | 15.70 | 0.52 | | 7.50 | ^[36]^ | |
| iBu-BTDC | 0.93 | | 16.50 | 0.60 | | 9.30 | ^[36]^ | |
| DTICPF | 0.91 | | 17.49 | 0.59 | | 9.36 | ^[37]^ | |
| DTICPT | 0.88 | | 15.23 | 0.53 | | 7.13 | [37] | |
| BT | 0.86 | | 17.91 | 0.68 | | 10.53 | This work | |


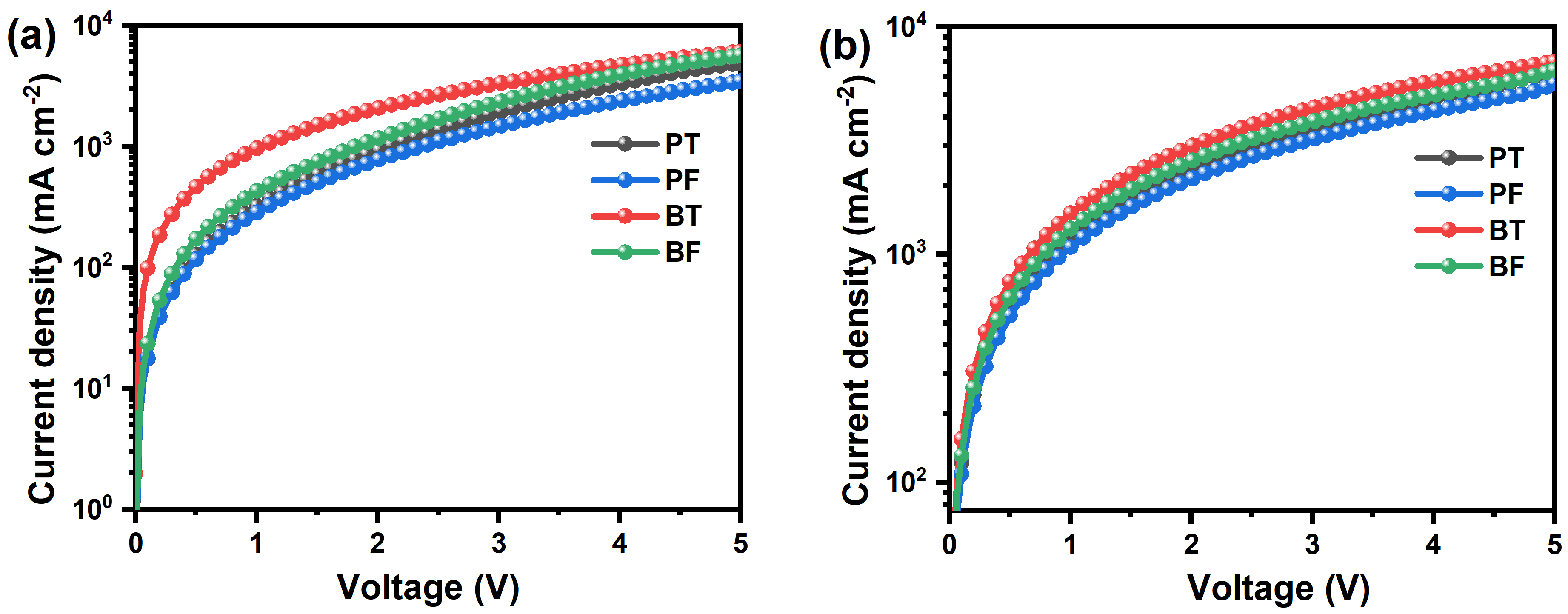


**Figure S37.** (a) Hole mobility and (b) electron mobility for the PT, PF, BT, and BF based devices.


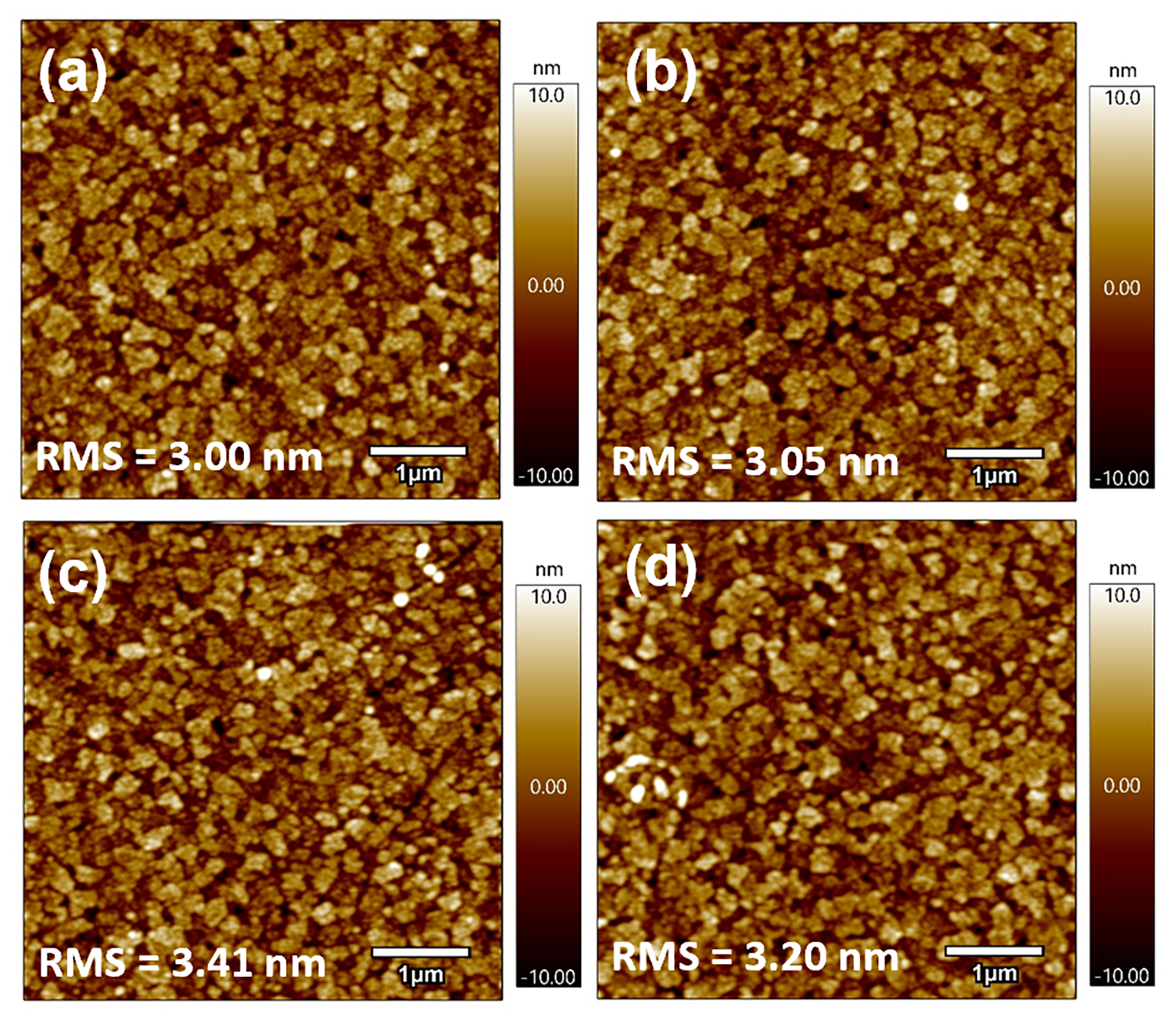


**Figure** **S38.** The AFM images of PT: C_70_ (a), PF: C_70_ (b), BT: C_70_ (c) and BF: C_70_ (d) blend films.


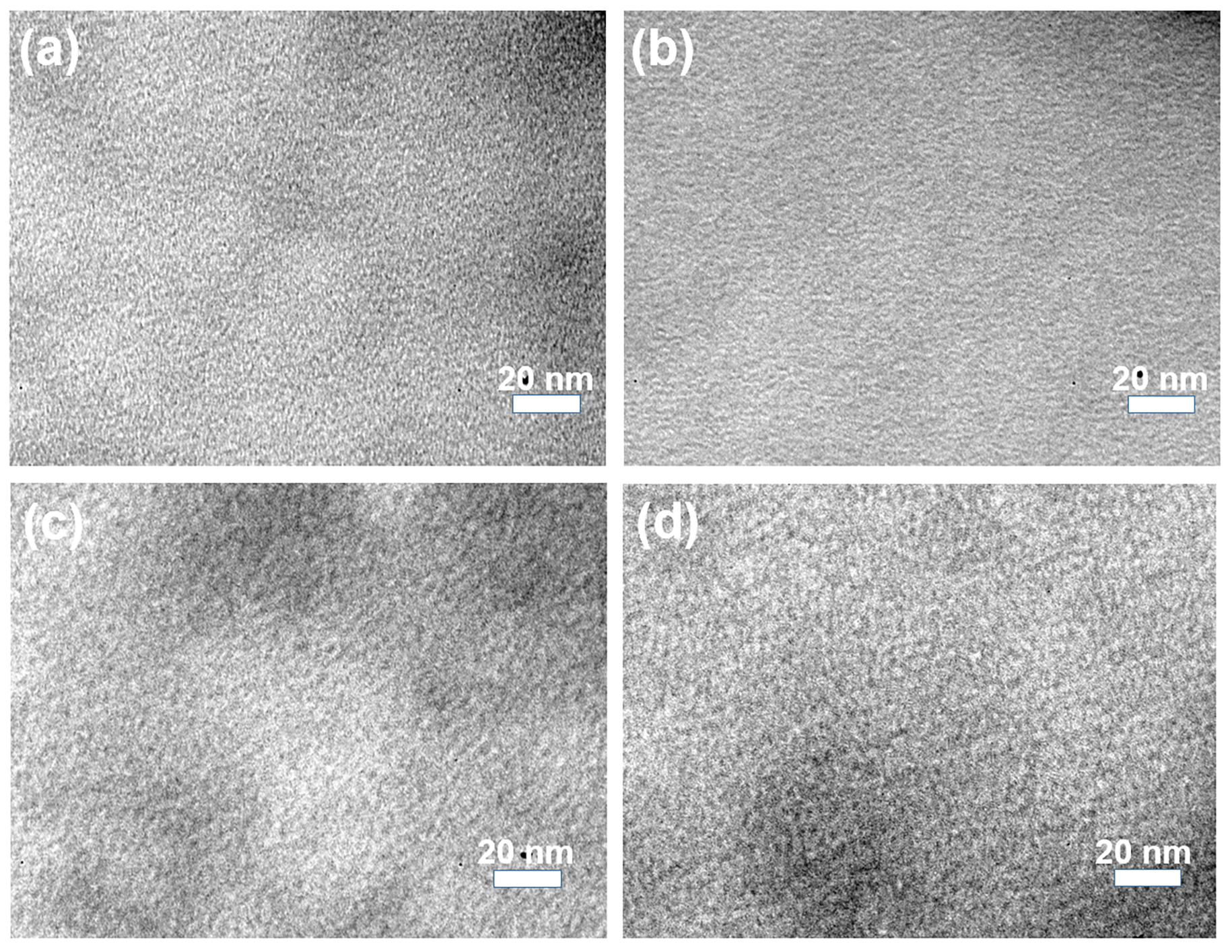


**Figure** **S39.** The TEM images of PT: C_70_ (a), PF: C_70_ (b), BT: C_70_ (c), and BF: C_70_ (d) blend films.


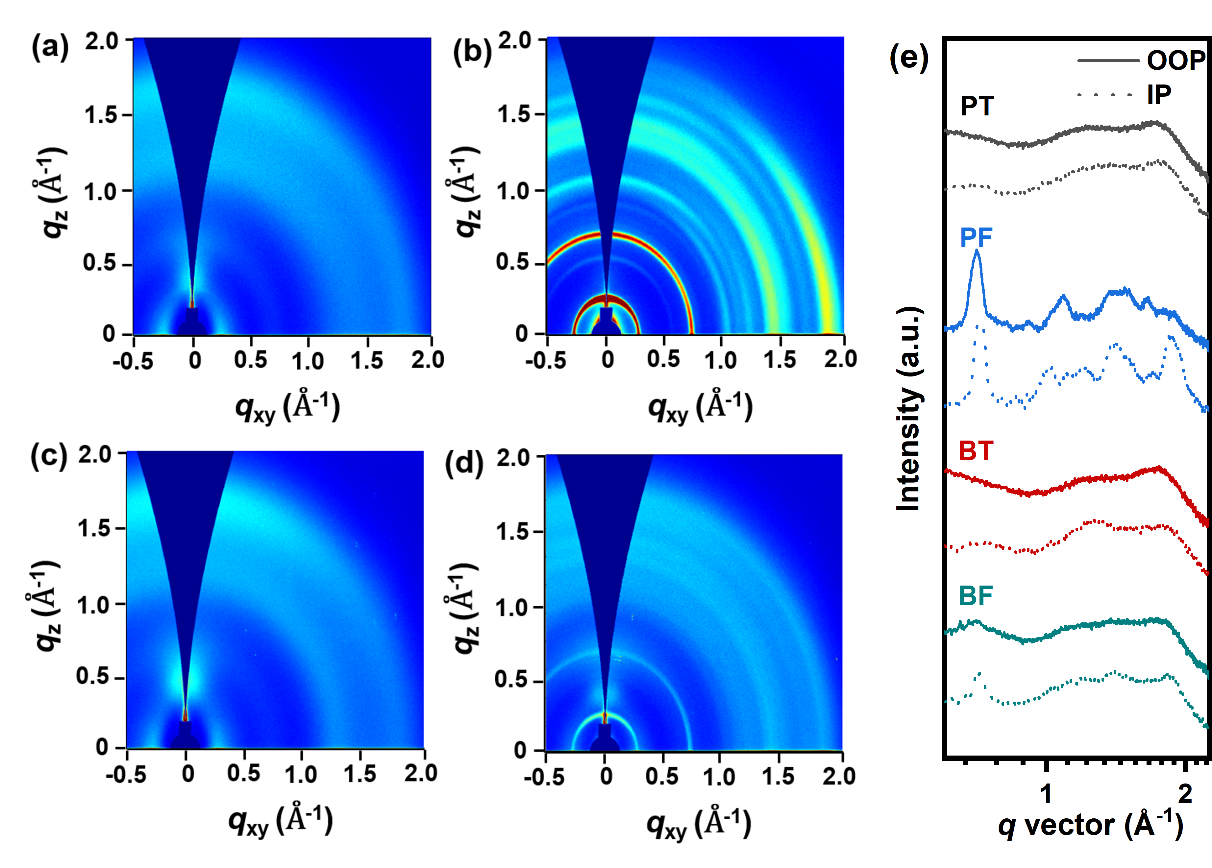


**Figure** **S40.** 2D GIWAXS patterns of PT (a), PF (b), BT (c) and BF (d) neat films. (e) The corresponding line-cut profiles along in-plane and out-of-plane directions.


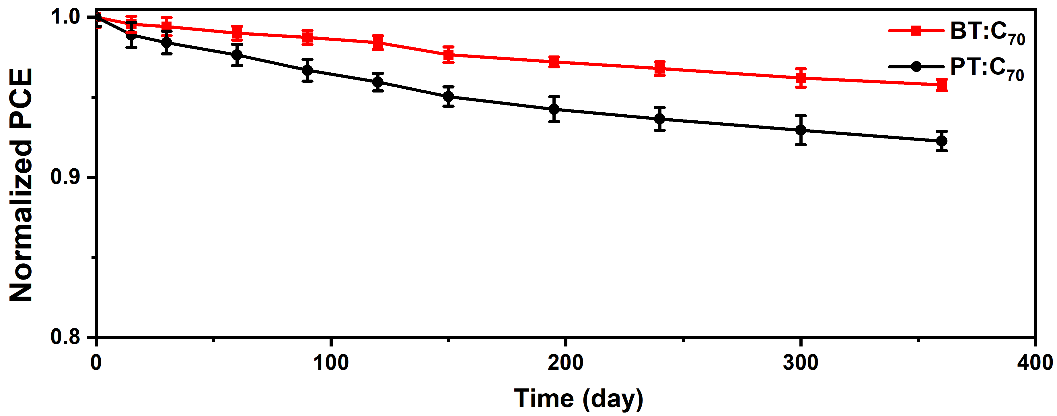


**Figure S41.** Storage stability of BT: C_70_ and PT: C_70_ devices.


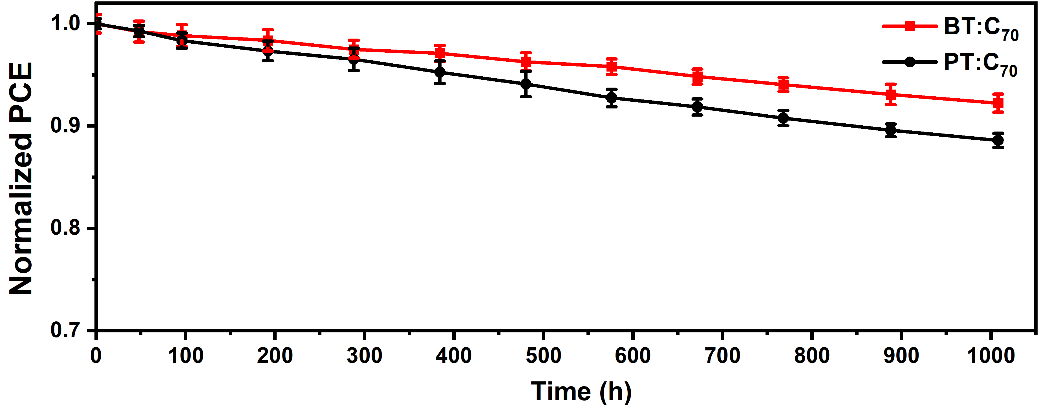


**Figure S42.** Thermal stability of BT: C_70_ and PT: C_70_ devices.

**References**

1. P. Zhang, Y. Zhang, D. Yang, et al., *Journal of Molecular Structure* **2024**, *1304*, 137627.

2. Y. J. Chang, T. J. Chow, *Tetrahedron* **2009**, *65* (46), 9626.

3. M. J. Frisch, G. W. Trucks, H. B. Schlegel, et al., Gaussian 16, Revision A.03, Gaussian, Inc., Wallingford CT, **2016**.

4. T. Lu, F. Chen, *Journal of Molecular Graphics and Modelling* **2012**, *38*, 314.

5. J. Zhang, T. Lu, *Physical Chemistry Chemical Physics* **2021**, *23* (36), 20323.

6. T. Lu, F. Chen, *Acta Chimica Sinica* **2011**, *69*, 2393.

7. T. Lu, F. Chen, *Journal of Computational Chemistry* **2011**, *33* (5), 580.

8. W. Humphrey, A. Dalke, K. Schulten, *Journal of Molecular Graphics* **1996** *14*, 33.

9. G. M. Sheldrick, *Acta Crystallographica Section A Foundations and Advances* **2015**, *71* (1), 3.

10. L. J. Bourhis, O. V. Dolomanov, R. J. Gildea, J. A. K. Howard, H. Puschmann, *Acta Crystallographica Section A Foundations and Advances* **2015**, *71* (1), 59.

11. H. Kageyama, H. Ohishi, M. Tanaka, Y. Ohmori, Y. Shirota, *Advanced Functional Materials* **2009**, *19* (24), 3948.

12. C.-Y. Chan, Y.-C. Wong, M.-Y. Chan, S.-H. Cheung, S.-K. So, V. W.-W. Yam, *ACS Applied Materials & Interfaces* **2016**, *8* (37), 24782.

13. C. K. Wang, X. Che, Y. C. Lo, et al., *Chemistry - An Asian Journal* **2020**, *15* (16), 2520.

14. J. W. Choi, C.-H. Kim, J. Pison, et al., *RSC Advances* **2014**, *4* (10), 5236.

15. J. Kim, H.-S. Shim, H. Lee, M.-S. Choi, J.-J. Kim, Y. Seo, *The Journal of Physical Chemistry C* **2014**, *118* (22), 11559.

16. V. Steinmann, N. M. Kronenberg, M. R. Lenze, et al., *Advanced Energy Materials* **2011**, *1* (5), 888.

17. A. Arjona-Esteban, J. Krumrain, A. Liess, et al., *Journal of the American Chemical Society* **2015**, *137* (42), 13524.

18. J. Meiss, M. Hummert, H. Ziehlke, K. Leo, M. Riede, *physica status solidi (RRL) – Rapid Research Letters* **2010**, *4* (11), 329.

19. C. Uhrich, R. Schueppel, A. Petrich, et al., *Advanced Functional Materials* **2007**, *17* (15), 2991.

20. R. Fitzner, E. Mena-Osteritz, A. Mishra, et al., *Journal of the American Chemical Society* **2012**, *134* (27), 11064.

21. C.-L. Chung, C.-Y. Chen, H.-W. Kang, et al., *Organic Electronics* **2016**, *28*, 229.

22. T. D. Leitner, A. Vogt, D. Popović, et al., *Materials Chemistry Frontiers* **2018**, *2* (5), 959.

23. C. Wetzel, A. Mishra, E. Mena-Osteritz, K. Walzer, M. Pfeiffer, P. Bäuerle, *Journal of Materials Chemistry C* **2016**, *4* (17), 3715.

24. K.-W. Chen, L.-Y. Lin, Y.-H. Li, et al., *Organic Electronics* **2018**, *52*, 342.

25. L.-C. Chi, H.-F. Chen, W.-Y. Hung, et al., *Solar Energy Materials and Solar Cells* **2013**, *109*, 33.

26. G. Chen, Z. Ling, B. Wei, et al., *Frontiers in Chemistry* **2018**, *6*, 412.

27. Y.-H. Cheng, H.-L. Wong, E. Y.-H. Hong, S.-L. Lai, M.-Y. Chan, V. W.-W. Yam, *ACS Applied Energy Materials* **2020**, *3* (3), 3059.

28. T.-y. Li, J. Benduhn, Z. Qiao, et al., *The Journal of Physical Chemistry Letters* **2019**, *10* (11), 2684.

29. T.-y. Li, T. Meyer, Z. Ma, et al., *Journal of the American Chemical Society* **2017**, *139* (39), 13636.

30. T.-y. Li, J. Benduhn, Y. Li, et al., *Journal of Materials Chemistry A* **2018**, *6* (38), 18583.

31. D. Credgington, Y. Kim, J. Labram, T. D. Anthopoulos, J. R. Durrant, *The Journal of Physical Chemistry Letters* **2011**, *2* (21), 2759.

32. J. Xue, B. P. Rand, S. Uchida, S. R. Forrest, *Advanced Materials* **2005**, *17* (1), 66.

33. K. Cnops, B. P. Rand, D. Cheyns, B. Verreet, M. A. Empl, P. Heremans, *Nature Communications* **2014**, *5* (1), 3406.

34. C.-H. Chen, H.-C. Ting, Y.-Z. Li, et al., *ACS Applied Materials & Interfaces* **2019**, *11* (8), 8337.

35. O. L. Griffith, X. Liu, J. A. Amonoo, et al., *Physical Review B* **2015**, *92* (8), 085404.

36. X. Che, C. L. Chung, C. C. Hsu, F. Liu, K. T. Wong, S. R. Forrest, *Advanced Energy Materials* **2018**, *8* (19), 1703603.

37. B. W. Chen, K. Cao, X. Wang, et al., *Small* **2024**, *20* (43), 2403486.
